# A systematic analysis of *Trypanosoma brucei* chromatin factors identifies novel protein interaction networks associated with sites of transcription initiation and termination

**DOI:** 10.1101/gr.275368.121

**Published:** 2021-11

**Authors:** Desislava P. Staneva, Roberta Carloni, Tatsiana Auchynnikava, Pin Tong, Juri Rappsilber, A. Arockia Jeyaprakash, Keith R. Matthews, Robin C. Allshire

**Affiliations:** 1 Wellcome Centre for Cell Biology and Institute of Cell Biology, School of Biological Sciences, University of Edinburgh, Edinburgh EH9 3BF, United Kingdom;; 2 Institute of Immunology and Infection Biology, School of Biological Sciences, University of Edinburgh, Edinburgh EH9 3JT, United Kingdom;; 3 Institute of Biotechnology, Technische Universität, 13355 Berlin, Germany

## Abstract

Nucleosomes composed of histones are the fundamental units around which DNA is wrapped to form chromatin. Transcriptionally active euchromatin or repressive heterochromatin is regulated in part by the addition or removal of histone post-translational modifications (PTMs) by “writer” and “eraser” enzymes, respectively. Nucleosomal PTMs are recognized by a variety of “reader” proteins that alter gene expression accordingly. The histone tails of the evolutionarily divergent eukaryotic parasite *Trypanosoma brucei* have atypical sequences and PTMs distinct from those often considered universally conserved. Here we identify 65 predicted readers, writers, and erasers of histone acetylation and methylation encoded in the *T. brucei* genome and, by epitope tagging, systemically localize 60 of them in the parasite's bloodstream form. ChIP-seq shows that 15 candidate proteins associate with regions of RNAPII transcription initiation. Eight other proteins show a distinct distribution with specific peaks at a subset of RNAPII transcription termination regions marked by RNAPIII-transcribed tRNA and snRNA genes. Proteomic analyses identify distinct protein interaction networks comprising known chromatin regulators and novel trypanosome-specific components. Notably, several SET- and Bromo-domain protein networks suggest parallels to RNAPII promoter–associated complexes in conventional eukaryotes. Further, we identify likely components of TbSWR1 and TbNuA4 complexes whose enrichment coincides with the SWR1-C exchange substrate H2A.Z at RNAPII transcription start regions. The systematic approach used provides details of the composition and organization of the chromatin regulatory machinery in *T. brucei* and establishes a route to explore divergence from eukaryotic norms in an evolutionarily ancient but experimentally accessible eukaryote.

Nucleosomes are composed of eight highly conserved core histone subunits (two each of H2A, H2B, H3, and H4) around which ∼147 bp of DNA is wrapped. Nucleosomes are organized into chromatin fibers that provide the dynamic organizational platform underpinning eukaryotic gene expression regulation. Formation of transcriptionally active and silent chromatin states depends on the presence of DNA methylation (Suzuki and Bird 2008), histone variants (Henikoff and Smith 2015), and histone post-translational modifications (PTMs) (Bannister and Kouzarides 2011). Repressive heterochromatin generally concentrates at the nuclear periphery, whereas active euchromatin localizes to the nuclear interior and can also associate with nuclear pores (Taddei et al. 2010; Lemaître and Bickmore 2015). Molecular understanding of the composition and function of distinct chromatin types in nuclear architecture and gene expression regulation is most advanced in well-studied eukaryotic models (plants, yeasts, animals) (Allshire and Madhani 2018). However, these represent only two eukaryotic supergroups, whereas distinct early-branching lineages have highly divergent histones and chromatin-associated regulators. One particularly tractable model for early branching eukaryotes is *Trypanosoma brucei*, the causative agent of human sleeping sickness and livestock nagana in Africa, which has evolved separately from the main eukaryotic lineage for at least 500 million years. Reflecting their evolutionary divergence, detailed analyses of these parasites have revealed numerous examples of biomolecular novelty, including RNA editing of mitochondrial transcripts (Shapiro and Englund 1995), polycistronic transcription of nuclear genes (Borst 1986; Tschudi and Ullu 1988), and segregation of chromosomes via an unconventional kinetochore apparatus comprising components distinct from other eukaryotic groups (Akiyoshi and Gull 2014).

During its life cycle, *T. brucei* alternates between a mammalian host and the tsetse fly vector, a transition accompanied by extensive changes in gene expression, leading to surface proteome alterations as well as metabolic reprogramming of the parasite (Matthews 2005; Smith et al. 2017). In the mammalian host, bloodstream form (BF) parasites are covered by a dense surface coat made of variant surface glycoprotein (VSG). Only a single VSG gene is expressed from an archive consisting of approximately 2000 VSG genes and gene fragments (Horn 2014). Periodically, *T. brucei* switches to express a new VSG protein to which no host antibodies have been produced, contributing to cyclical parasitaemia. Parasites taken up by the tsetse during blood meals differentiate in the fly midgut to the procyclic form (PF), which replaces all VSGs with procyclin surface proteins (Roditi and Liniger 2002).

The *T. brucei* genome encodes four core histones (H2A, H2B, H3, H4) and a variant for each core histone type (H2A.Z, H2B.V, H3.V, H4.V), but it lacks a centromere-specific CENP-A/cenH3 variant (Akiyoshi and Gull 2014). All trypanosome histones differ significantly in their amino acid sequence from their counterparts in conventional eukaryotes (Thatcher and Gorovsky 1994; Lowell and Cross 2004; Lowell et al. 2005; Mandava et al. 2007). For example, lysine 9 of histone H3, the methylation of which specifies heterochromatin formation in many eukaryotes, is not conserved. Nonetheless, many histone PTMs have been detected in *T. brucei* and its relative *Trypanosoma cruzi* (Janzen et al. 2006a; Mandava et al. 2007; de Jesus et al. 2016; de Lima et al. 2020; Kraus et al. 2020), although only a handful of these have been characterized in some detail.

Unusually for a eukaryote, most trypanosome genes are transcribed in polycistronic units that are resolved by *trans*-splicing of a spliced leader (SL) RNA sequence to the 5′ end of the mRNA and polyadenylation at the 3′ end (Günzl 2010). RNAPII transcription usually initiates from broad (∼10-kb) GT-rich divergent transcription start regions (TSRs; comparable to promoters of other eukaryotes) enriched in nucleosomes containing the H2A.Z and H2B.V histone variants as well as the H3K4me (methylation) and H4K10ac (acetylation) PTMs (Siegel et al. 2009; Wright et al. 2010; Wedel et al. 2017). Conversely, RNAPII transcription typically terminates at regions of convergent transcription known as transcription termination regions (TTRs) that are marked by the presence of the DNA modification base J and the H3.V and H4.V histone variants (Siegel et al. 2009; Schulz et al. 2016). Less frequently, *T. brucei* transcription units are arranged head-to-tail, requiring termination ahead of downstream TSRs. TTRs between such transcription units are often coincident with RNAPI- or RNAPIII-transcribed genes, which may act as boundaries that block the passage of advancing RNAPII into downstream transcription units (Marchetti et al. 1998; Siegel et al. 2009; Maree and Patterton 2014).

The consensus view is that trypanosome gene expression is regulated predominantly post-transcriptionally via control of RNA stability and translation (Clayton 2019). Nonetheless, numerous putative chromatin regulators can be identified as coding sequences in the *T. brucei* genome (Berriman et al. 2005), but their functional contexts are largely unexplored. Here we undertake cellular localization, genome-wide chromatin association, and proteomic analyses of bioinformatically identified putative readers, writers, and erasers of histone acetyl and methyl marks encoded in the trypanosome genome. The results presented provide an entry point for understanding similarities and differences between the transcriptional regulatory machinery of this divergent eukaryote and the eukaryotic norm.

## Results and discussion

### Identification of putative chromatin regulators

We identified 65 putative regulators or interpreters of histone lysine acetylation and methylation by interrogating the trypanosome genome database (TriTrypDB; https://tritrypdb.org/tritrypdb) and using additional homology-based searches with known chromatin reader, writer, and eraser domains (Table 1; Supplemental Fig. S1; Supplemental Table S1). These approaches allowed us to detect 16 potential readers with the following domains: Bromo (Haynes et al. 1992), PHD (Aasland et al. 1995), Tudor (Ponting 1997), Chromo (Paro 1990; Singh et al. 1991), PWWP (Stec et al. 2000), and Znf-CW (Perry and Zhao 2003). With respect to writers of histone modifications, we found six potential histone acetyltransferases (HATs) belonging to the MYST (MOZ/SAS-related) and GNAT (ELP3-related) families (Lee and Workman 2007), as well as 29 SET domain (Dillon et al. 2005) and three DOT domain (Feng et al. 2002) putative histone methyltransferases (HMTs). We also analyzed several predicted acetyltransferases with nonhistone substrates: one lysophospholipid acyltransferase (LPLAT) (Shindou and Shimizu 2009) and two GNAT family (RimI-related) *N*-acetyltransferases (NATs) (Vetting et al. 2008) plus the noncatalytic EAF6 component of the NuA4 HAT complex (Roth et al. 2001; Hishikawa et al. 2008). Our searches for potential erasers of histone PTMs identified seven histone deacetylases (Class I, Class II, and Sir2-related HDACs) (Grozinger and Schreiber 2002) and four JmjC domain demethylases (Klose et al. 2006). The function of some of these putative chromatin regulators has been explored previously (Supplemental Table S2; Figueiredo et al. 2009; Maree and Patterton 2014).

**Table 1.**
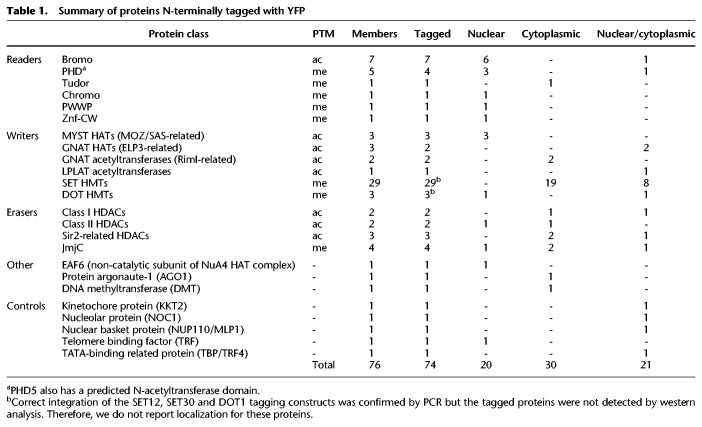
Summary of proteins N-terminally tagged with YFP

To examine these putative writers, readers, and erasers of histone PTMs, each was YFP-tagged and localized in BF parasites. AGO1 (Shi et al. 2009) and the putative DNA methyltransferase DMT (Militello et al. 2008) were also included in our analysis because they are associated with chromatin-based silencing in other eukaryotes (Kloc and Martienssen 2008; Suzuki and Bird 2008; Allshire and Madhani 2018).

Additionally, we examined several control proteins with known distinctive nuclear distributions; these were the trypanosome kinetochore protein KKT2 (Akiyoshi and Gull 2014), the nucleolar protein NOC1 (Alsford and Horn 2012), the nuclear pore basket protein NUP110/MLP1 (DeGrasse et al. 2009), as well as the telomere repeat-binding factor TRF (Li et al. 2005) and the TATA-binding related protein TBP/TRF4 (Ruan et al. 2004). AGO1 served as a control for cytoplasmic localization (Shi et al. 2004).

### Cellular localization of the putative chromatin regulators

Candidate proteins were endogenously tagged with YFP in BF parasites of the *T. brucei* Lister 427 strain. Proteins were tagged N-terminally to avoid interference with 3′ UTR sequences involved in mRNA stability control (Clayton 2019). Although the presence or position of the YFP tag might affect individual protein expression and localization patterns, this approach provided a consistent pipeline for the systematic analysis of the proteins on our candidate list. Of the 76 selected proteins (including controls), 74 were successfully tagged, whereas cells expressing YFP-PHD3 and YFP-ELP3c were not obtained. The tagging constructs for SET12, SET30, and DOT1 were correctly integrated, but YFP-tagged proteins were not detectable by western analysis, suggesting tag failure, low protein abundance, or no expression in BF parasites.

**Figure 1.**
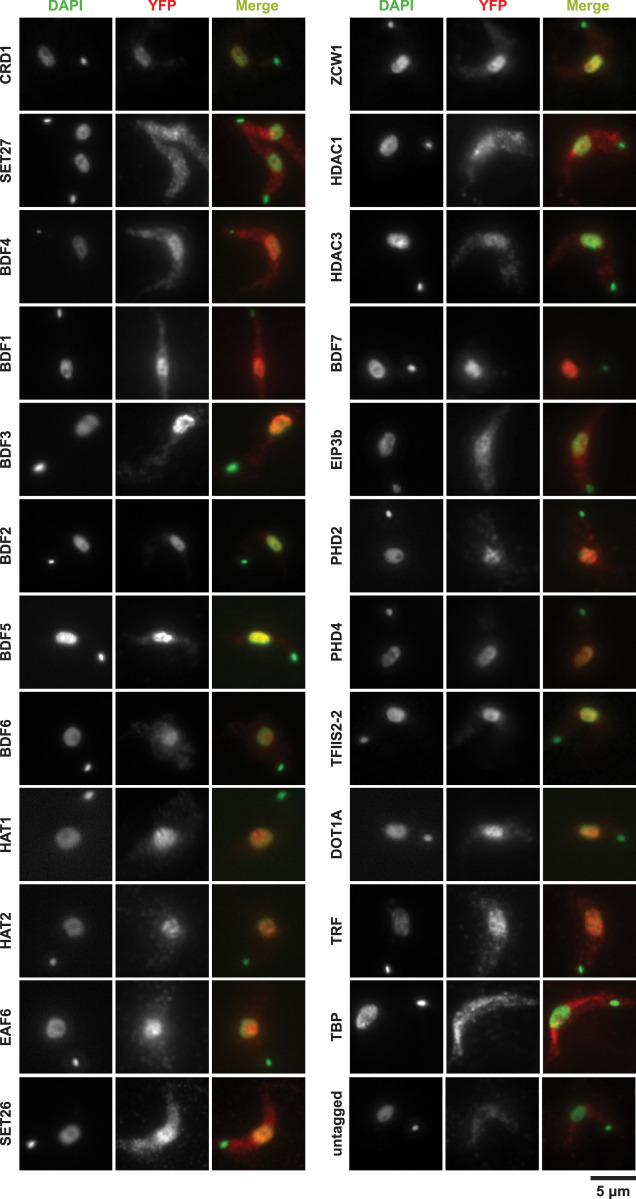
Cellular localization of chromatin-associated *T. brucei* candidate proteins. The indicated YFP-tagged proteins expressed in bloodstream Lister 427 cells from their endogenous genomic loci were detected with an anti-GFP primary antibody and an Alexa Fluor 568–labeled secondary antibody (red). Nuclear and kinetoplast (mitochondrial) DNA were stained with DAPI (green). Staining of untagged 427 parasites serves as a negative control. Representative images are shown for those candidate proteins that gave a specific ChIP-seq signal. The images are ordered according to ChIP-seq patterns shown in Figure 2A and Figure 5A. Images for all other tagged proteins are included in Supplemental Figure S2. Scale bar, 5 µm.

Immunolocalization with anti-GFP antibodies was used to identify proteins residing in the nucleus that might associate with chromatin. The control proteins showed localization patterns expected for telomeres (TRF; nuclear foci), TATA-binding protein (TBP; nuclear foci), kinetochores (KKT2; nuclear foci), nucleolus (NOC1; single nuclear compartment), nuclear pores (NUP110; nuclear rim), and cytoplasm (AGO1; gap in the staining coincident with the nucleus) (Supplemental Fig. S2). Some cytoplasmic signal was also detected for nuclear control proteins, but this may correspond to background staining as evidenced by the signal observed in untagged cells (Fig. 1; Supplemental Fig. S2). Selected proteins were also imaged with and without antibody staining and for different exposure times (Supplemental Fig. S3). From this, we concluded that fluorescence signal enhancement through antibody staining was required for optimal imaging of most proteins and that different exposure times were needed to adjust to the expression level of each protein. Images with multiple cells per field in different cell cycle stages are also provided (Supplemental Fig. S4).

Of the YFP-tagged proteins, 20 were exclusively nuclear, 30 showed only a cytoplasmic localization, and 21 were found in both compartments (Table 1; Supplemental Table S1). Twenty-three candidate and control proteins with exclusive or some nuclear localization were subsequently found to associate with sites of RNAPII transcription initiation and/or with a subset of RNAPII TTRs coincident with RNAPIII-transcribed genes (see below; Fig. 1), whereas the remaining proteins that were not detected on chromatin displayed all three localization patterns (Supplemental Fig. S2). Consistent with previous observations, HAT1–3 decorated nuclear substructures (Kawahara et al. 2008), as did the predicted EAF6 subunit of the NuA4 HAT complex. In contrast, LPLAT1 gave both nuclear and cytoplasmic signal, whereas both NAT2 and NAT3 localized to the cytoplasm (Supplemental Fig. S2). YFP-tagged ELP3a and ELP3b GNAT acetyltransferases showed nuclear and some cytoplasmic signal. GFP-ELP3b was previously reported to be concentrated in the nucleolus (Alsford and Horn 2011); this difference may be a consequence of using an ectopic overexpression system in that study. Moreover, we did not detect enrichment of ELP3b over rDNA transcription units by chromatin immunoprecipitation and sequencing (ChIP-seq). Of the 29 identified putative SET-domain methyltransferases, only eight showed some nuclear localization, whereas most were concentrated in the cytoplasm. Examination of cells expressing YFP-tagged predicted reader proteins revealed that BDF1–3, BDF5–7, PHD1, PHD2, PHD4, CRD1, TFIIS2-2, and ZCW1 were exclusively nuclear, whereas BDF4 and PHD5 displayed both nuclear and cytoplasmic localization, and the sole Tudor domain protein TDR1 was cytoplasmic. In agreement with previous analyses (Wang et al. 2010), HDAC3 was predominantly nuclear, whereas DAC1 (referred to as HDAC1 here) was nuclear/cytoplasmic, and both HDAC2 and HDAC4 resided in the cytoplasm. The Sir2-related proteins SIR2RP1 and Sir2rp2 localized mostly to the cytoplasm, whereas Sir2rp3 was detected in the nucleus as well as in the cytoplasm. Of the four identified putative demethylases, JMJ2 was nuclear, JMJ1 and CLD1 were cytoplasmic, and LCM1 was found in both compartments. Both AGO1 and DMT were cytoplasmic (Supplemental Fig. S2), suggesting that they are unlikely to be involved in directing chromatin or DNA modifications.

Of the 71 proteins we successfully tagged, expressed, and localized in BF cells, 65 have also been also tagged by the TrypTag project in PF cells (Supplemental Table S1; Dean et al. 2017). For most proteins, our results are in agreement with the TrypTag data, and any discrepancies may be indicative of differences in protein localization between the different developmental forms of *T. brucei*.

### Many putative chromatin regulators accumulate at RNAPII TSRs

**Figure 2.**
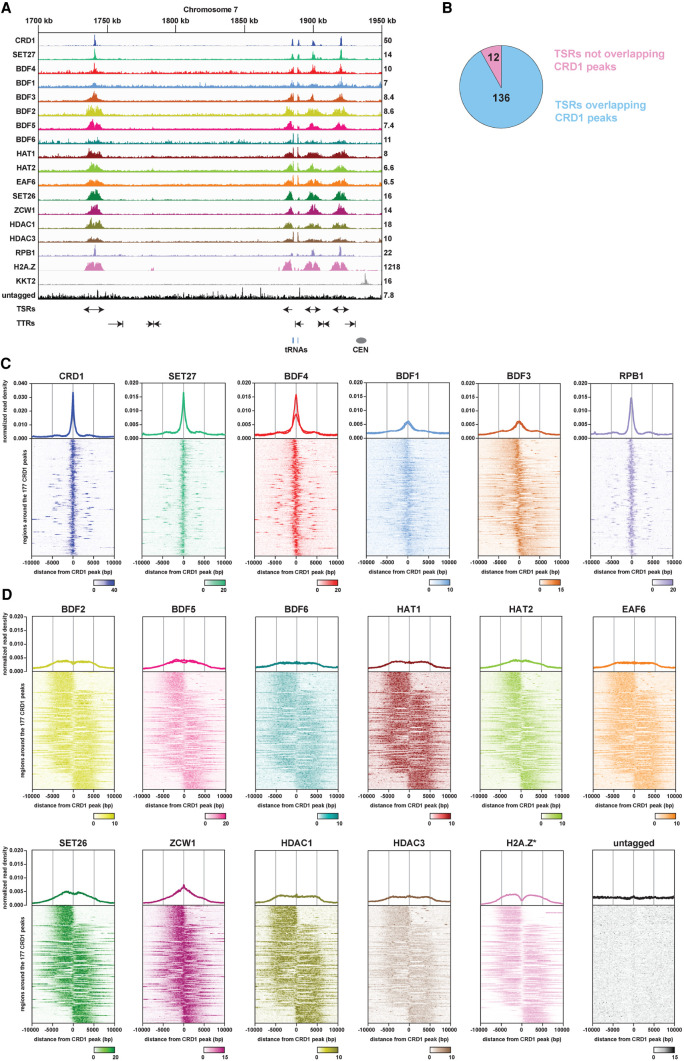
ChIP-seq reveals two classes of proteins at *T. brucei* transcription start regions (TSRs). (*A*) A region of Chromosome 7 (coordinates as indicated; kilo bases) is shown with ChIP-seq reads mapped for the indicated proteins. A single replicate is shown for each protein. Tracks are scaled separately as reads per million (values shown at the end of each track). The kinetochore protein KKT2 is included as a positive control and is enriched at centromeric regions. ChIP-seq performed in Lister 427 cells expressing no tagged protein (untagged) provides a negative control. ChIP-seq profiles for the different proteins are ordered according to their patterns. Previous H2A.Z data (Wedel et al. 2017) and our RPB1 ChIP-seq allowed TSRs to be identified. No input data were available for the normalization of H2A.Z reads, resulting in a higher read scale. The position of bidirectional/divergent and unidirectional/single TSRs is indicated with arrows showing the direction of transcription. The position of convergent and single transcription termination regions (TTRs) is shown with arrows indicating the direction from which transcription is halted. The position of tRNA genes (blue bars) and the Chromosome 7 centromere (CEN; gray oval) are marked. Positions of the various genomic elements (TSRs, TTRs, tRNAs, CEN) were obtained from annotations of the Lister 427 genome (Müller et al. 2018). (*B*) Most TSRs annotated in the Lister 427 genome overlap with YFP-CRD1 ChIP-seq peaks. (*C*) Enrichment profiles of Class I TSR factors. The metagene plots (*top*) show normalized read density around all CRD1 peak summits, with individual replicates for each protein shown separately. Note the different scale for CRD1. The heatmaps (*bottom*) are an average of all replicates for each protein and show protein density around individual CRD1 peaks. Scale bars, reads that were normalized to input and library size. (*D*) As in *C*, for Class II TSR factors.

We performed ChIP-seq for all expressed candidate and control proteins except TDR1 and NOC1 (69 in total) to determine which proteins associate with chromatin and to assess their distribution across the *T. brucei* genome. Our rationale was that ChIP-seq might register chromatin association even if cellular localization analysis reported a protein to be predominantly cytoplasmic. As expected, the kinetochore control protein KKT2 was specifically enriched over centromeric regions (Fig. 2A). Consistent with their cytoplasmic localizations, AGO1 and DMT registered no ChIP-seq signal (Supplemental Fig. S5). Moreover, under our standard fixation and ChIP-seq conditions, no specific enrichment over any genomic region was detected for 45 of the YFP-tagged proteins, including several that showed clear nuclear localization (Supplemental Fig. S5; Supplemental Table S3).

In total, 24 of the YFP-tagged proteins assayed (including the KKT2 control) gave specific enrichment patterns across the *T. brucei* genome. Fifteen of our candidate proteins displayed enrichment that was adjacent to, or overlapping with, the previously reported H2A.Z peaks, indicating that these proteins are enriched at known RNAPII TSRs (Fig. 2A; Supplemental Fig. S6A). Our ChIP-seq data confirmed that the largest RNAPII subunit (RPB1) does indeed show peaks at the same locations. The TSR-associated proteins include BDF1–6, CRD1, EAF6, HAT1, HAT2, DAC1/HDAC1, HDAC3, SET26, SET27, and ZCW1. These 15 proteins were always enriched together at RNAPII TSRs. Some of these proteins also presented peaks that coincide with those of RNAPIII/TTR-enriched factors in a few locations (see below; Supplemental Fig. S6C), but otherwise, they did not display significant association with any other genomic regions. The fact that six Bromo domain proteins, two HATs, and a NuA4 component are included in this set is consistent with these acting together to promote RNAPII-mediated transcription, a hallmark of which is histone acetylation (Roth et al. 2001). Indeed, BDF1, BDF3, and BDF4 have previously been shown to be enriched at sites of *T. brucei* RNAPII transcription initiation where nucleosomes show HAT1-mediated acetylation of H2A.Z and H2B.V and HAT2-mediated acetylation of histone H4, which are important for normal RNAPII transcription from these regions (Siegel et al. 2009; Schulz et al. 2015; Kraus et al. 2020). The remaining 10 proteins we identified as being associated with TSRs have not been previously shown to act at kinetoplastid RNAPII promoters.

### Putative chromatin regulators show two distinct TSR association patterns

In yeast, in which many RNAPII genes are transcriptionally regulated, a group of chromatin regulators are enriched specifically at promoters, where they assist in transcription initiation, whereas others travel with RNAPII into gene bodies, aiding transcription elongation, splicing, and termination (Kaplan et al. 2003; Mason and Struhl 2003; Carrozza et al. 2005; Joshi and Struhl 2005; Keogh et al. 2005; Li et al. 2007; Cheung et al. 2008; Venkatesh and Workman 2013; Jonkers and Lis 2015). We therefore compared the enrichment profiles of the TSR-associated proteins relative to that of the Chromo domain protein CRD1, which displayed the sharpest and highest peak signal. We identified 177 CRD1 peaks across the *T. brucei* Lister 427 genome that overlap with 136 of the 148 annotated RNAPII TSRs (Fig. 2B). For each TSR-associated factor, normalized reads were assigned to 10-kb windows upstream of and downstream from all CRD1 peak summits. The general peak profile for each protein was then displayed as a metagene plot and the read distribution around individual CRD1 peaks represented as a heatmap (Fig. 2C,D; Supplemental Fig. S7). This analysis indicated that CRD1, SET27, BDF4, and, to a lesser extent, BDF1 and BDF3 show sharp peaks at all RNAPII TSRs, similar to RNAPII/RPB1 itself. We refer to these as Class I TSR-associated factors (Fig. 2C). The remaining 10 proteins were more broadly enriched over the same regions with a slight trough evident in the signal for at least five (BDF2, BDF5, HAT1, HAT2, SET26), perhaps indicative of two adjacent peaks as observed for H2A.Z (Fig. 2D). We refer to these as Class II TSR-associated factors. Class II factor profiles are similar to the previously reported H2A.Z enrichment pattern (Siegel et al. 2009), with the signal gradually declining over 5- to 10-kb regions on either side of the CRD1 peak summit. We observed similar peak widths of Class II factors across all TSRs regardless of polycistron length; thus, there is no apparent relationship between TSR size and the length of the downstream polycistronic transcription unit.

**Figure 3.**
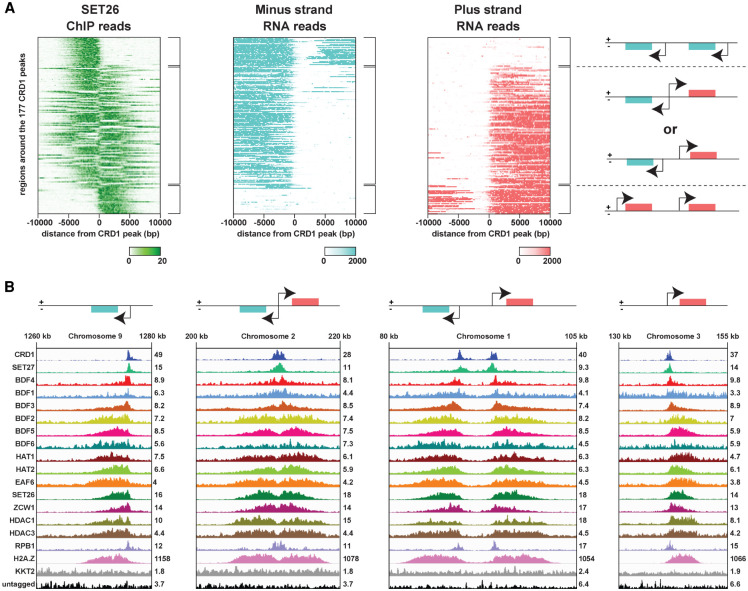
Enrichment of Class II proteins at TSRs follows the direction of RNAPII transcription. (*A*) SET26 is used as a representative protein of Class II TSR-associated factors. Comparison of SET26 ChIP-seq data with strand-specific RNA-seq data (Naguleswaran et al. 2018) shows that SET26 reads are enriched in the same direction as RNAPII transcript reads. Heatmaps show from *top* to *bottom*: minus strand reads from unidirectional TSRs (*top*), plus and minus strand reads from bidirectional TSRs or from two adjacent unidirectional TSRs (*middle*), and plus strand reads from unidirectional TSRs (*bottom*). (*B*) Examples from the different heatmap regions described in *A*. A single replicate is shown for each protein. Tracks are scaled separately as reads per million (values shown at the end of each track).

Of the 148 annotated *T. brucei* RNAPII promoters, 99 are bidirectional, initiating production of stable transcripts in both directions, and 49 are unidirectional, driving transcription in just one direction. To investigate the relationship between transcription directionality and enrichment of our candidate proteins, we sorted the heatmaps of Class II factors by their distribution around CRD1 peaks. We then compared the sorted heatmaps with previously published RNA-seq data from which the direction of RNAPII transcription was derived (Naguleswaran et al. 2018). This analysis showed that Class II TSR-associated factors show specific enrichment in the same direction as RNAPII transcription initiated from all uni- and bidirectional promoters (Fig. 3A,B).

### Proteins associated with TSRs participate in discrete interaction networks

The analyses presented above suggest that, as in yeast, proteins that show either narrow (Class I) or broad (Class II) association patterns across RNAPII promoter regions might play different roles such as defining sites of RNAPII transcription initiation or facilitating RNAPII processivity through their association with chromatin and interactions with RNAPII auxiliary factors. Determining how these various activities are integrated through association networks should provide insight into how the distinct sets of proteins might influence RNAPII transcription. Therefore, we affinity-selected each tagged protein that registered a specific ChIP-seq signal at TSRs and identified their interacting partners by mass spectrometry.

**Figure 4.**
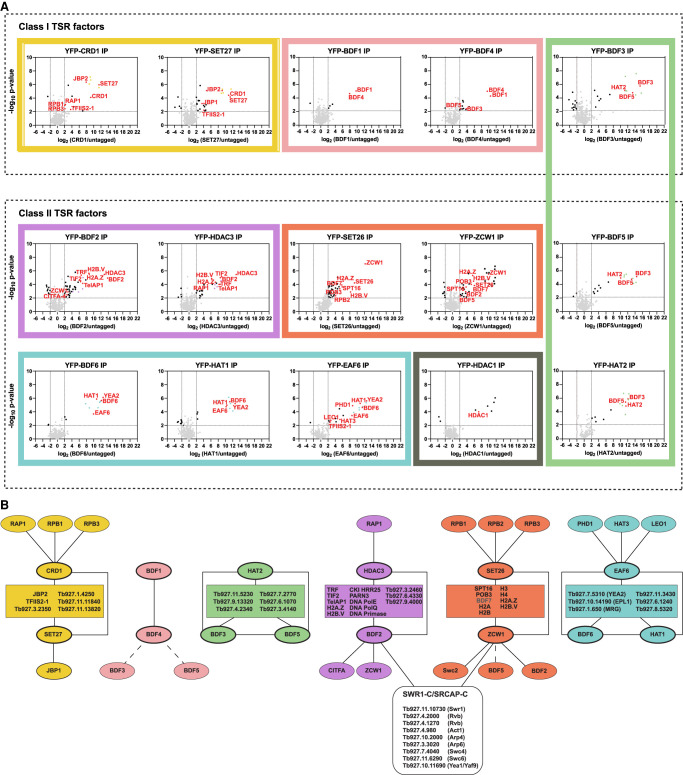
Class I and Class II TSR-associated factors define distinct interaction networks. (*A*) YFP-tagged proteins found to be enriched at TSRs were analyzed by LC-MS/MS to identify their protein interactions. The data for each plot is based on three biological replicates. Cutoffs used for significance: log_2_ (tagged/untagged) >2 or <−2 and *P* < 0.01 (Student's *t*-test). Enrichment scores for proteins identified in each affinity selection are presented in Supplemental Table S4. Plots in the same box show reciprocal interactions. Proteins of interest are indicated by the red font. Uncharacterized proteins common to several affinity selections are depicted in yellow, green, purple, and cyan. Black squares represent members of the SWR1/SRCAP complex found in the BDF2 and ZCW1 affinity selections. (*B*) Key proteins identified as being associated with the indicated YFP-tagged bait proteins (thick oval outlines). Rectangles contain proteins common to several affinity purifications. Lines denote interactions between proteins. The interactions of BDF3 and BDF5 with BDF4 (dashed lines); BDF5 with ZCW1 (dashed lines); and BDF7 (gray) with ZCW1 and SET26 were not confirmed by reciprocal affinity selections.

Below we detail the interaction networks for the TSR-associated factors (Fig. 4A,B; Supplemental Table S4) and the homologies identified in their key interacting partners through HHpred searches (Soding et al. 2005; Supplemental Table S5) and discuss possible functional implications arising from these proteomics data. Interacting partners identified by mass spectrometry analysis are also included for 10 proteins (PHD1, PHD5, HAT3, AGO1, NUP110, SET13, SET15, SET20, SET23, and SET25) for which no specific ChIP-seq signal was obtained (Supplemental Fig. S8).

### Class I: CRD1, SET27, BDF4, BDF1, and BDF3

#### CRD1 and SET27

Affinity selection of YFP-CRD1 and YFP-SET27 (Fig. 4A,B; Supplemental Table S4) revealed that they both associate strongly with each other, with four uncharacterized proteins (Tb927.1.4250, Tb927.3.2350, Tb927.11.11840, Tb927.11.13820) and with JBP2. JBP2 is a TET-related hydroxylase that catalyzes thymidine oxidation on route to the synthesis of the DNA modification base J, which is found at TTRs and telomeres in trypanosomes (Cliffe et al. 2010; Reynolds et al. 2016; Schulz et al. 2016). In addition, SET27 associates with JBP1, another TET-related thymidine hydroxylase involved in base J synthesis (Borst and Sabatini 2008). The Chromo domain of CRD1 shows marginal similarity when aligned with other Chromo domain proteins (Supplemental Fig. S1B); however, its reciprocal association with SET27 suggests that they might function together as a reader–writer pair at TSRs. In yeast and human cells, the Set1/SETD1 methyltransferase installs H3K4 methylation at promoters (Shilatifard 2012), and *T. brucei* SET27 might play a similar role at TSRs. Recently, methylated histone lysine residues were found to be prevalent at trypanosome TSRs (Kraus et al. 2020). SET27 likely catalyzes the methylation of at least one of these lysines, which may then be bound by CRD1, ensuring SET27 recruitment and persistence of the methylation event(s) that it installs on histones within resident TSR nucleosomes. The association of the RPB1 and RPB3 RNAPII subunits with CRD1 underscores its likely involvement in linking such chromatin modifications with transcription.

#### BDF1 and BDF4

Consistent with their colocalization in Class I ChIP-seq peaks, YFP-BDF1 and YFP-BDF4 showed strong reciprocal association with each other (Fig. 4A,B; Supplemental Table S4). BDF4 also showed weak association with BDF3 and the Class II factor BDF5. Bromo domains are known to bind acetylated histones (Zaware and Zhou 2019) and are thus presumably attracted to TSRs owing to the presence of highly acetylated histones, particularly H2A.Z, H2B.V, and H4, in resident nucleosomes (Kraus et al. 2020). The coincidence of the H2A.Z variant with histone modifications associated with active transcription in this evolutionarily distinct eukaryote suggests that they act together to recruit various chromatin remodeling and modification activities to ensure efficient transcription.

#### BDF3 (Class I), BDF5 (Class II), and HAT2 (Class II)

BDF3, BDF5, and HAT2 reciprocally associate with each other and a set of six uncharacterized proteins (Tb927.3.4140, Tb927.4.2340, Tb927.6.1070, Tb927.7.2770, Tb927.9.13320, Tb927.11.5230), suggesting that these nine proteins may act together in a complex (Fig. 4A,B; Supplemental Table S4). BDF3 interacts with both Class I and Class II proteins, suggesting that it may straddle the interface between both classes of factors at RNAPII TSRs. HAT2 mediates acetylation of histone H4 and promotes normal transcription initiation by RNAPII (Kraus et al. 2020). The Bromo domains of BDF3 and BDF5 may guide HAT2 to pre-existing acetylation at TSRs to maintain the required acetylated state for efficient transcription. We also note that Tb927.3.4140 displays similarity to poly ADP ribose polymerase (PARP) (Supplemental Table S5), suggesting that ribosylation might contribute to TSR definition by promoting chromatin decompaction as seen upon *Drosophila* heat shock puff induction (Tulin et al. 2003; Tulin and Spradling 2003; Sawatsubashi et al. 2004) and at some mammalian enhancer–promoter regions (Benabdallah et al. 2019). Tb927.11.5230 contains an EMSY ENT domain whose structure has been determined (Supplemental Table S5; Mi et al. 2018); such domains are present in several chromatin regulators. Tb927.4.2340 shows similarity to the C-terminal region of the vertebrate TFIID TAF1 subunit (Supplemental Table S5). Metazoan TAF1 bears two Bromo domains in its C-terminal region, whereas in yeast, the double Bromo domain component of TFIID is contributed by the separate Bdf1 (or Bdf2) proteins (Matangkasombut et al. 2000; Timmers 2020). *T. brucei* BDF5 contains two Bromo domains and may thus be equivalent to the yeast TFIID Bdf1 subunit.

### Class II: BDF2, BDF5, BDF6, EAF6, HAT1, HAT2, DAC1/HDAC1, HDAC3, SET26, ZCW1

#### BDF2 and HDAC3

We found that both the TSR-enriched histone variants H2A.Z and H2B.V strongly associate with BDF2 and HDAC3, which interact with each other as well as with four uncharacterized proteins (Tb927.3.2460, Tb927.6.4330, Tb927.9.4000, Tb927.9.8520) (Fig. 4A,B; Supplemental Table S4). We note that Tb927.9.8520 shows similarity to DNA polymerase epsilon (DNAPolE) (Supplemental Table S5), and both DNA polymerase theta (DNAPolQ) and DNA Primase were also enriched along with the PARN3 poly(A)–specific ribonuclease and Casein Kinase I isoform 2 (CK1.2). Moreover, Tb927.3.2460 shows similarity to a nuclear pore protein, whereas Tb927.6.4330, Tb927.9.4000, and DNAPolQ were previously shown to associate with the telomere proteins TRF and TelAP1, and DNA primase is known to interact with TelAP1 (Reis et al. 2018). Here we find that the telomere-associated proteins TRF, TIF2, TelAP1, and RAP1 are also enriched in both BDF2 and HDAC3 affinity selections. The significance of this association is unknown; however, BDF2 and HDAC3 have previously been shown to be required for telomeric VSG expression site silencing (Wang et al. 2010; Schulz et al. 2015), and RNAi knockdown of Tb927.6.4330 causes defects in telomere-exclusive VSG gene expression (Glover et al. 2016). It was also unexpected to observe HDAC3 enrichment at RNAPII TSRs given that actively transcribed regions tend to be hyperacetylated (Kouzarides 2007). HDAC3 may be required to reverse acetylation associated with newly deposited histones during S phase (Stewart-Morgan et al. 2020) or to remove acetylation added during expected H2A–H2B:H2A.Z–H2B.V dimer–dimer exchange events at TSR regions (Millar et al. 2006; Bonisch and Hake 2012).

#### SET26 and ZCW1

SET26 and ZCW1 associated with each other as well as with most histones and histone variants (Fig. 4A,B; Supplemental Table S4). In addition, both SET26 and ZCW1 showed strong interaction with the SPT16 and POB3 subunits of the facilitates chromatin transcription (FACT) complex, which is involved in trypanosome VSG silencing through increased histone occupancy at VSG expression sites (Denninger and Rudenko 2014) and is known to aid transcription elongation in other eukaryotes (Belotserkovskaya et al. 2003). SET26 could perform an analogous role to the yeast Set2 H3K36 HMT, which travels with RNAPII and, together with FACT, ensures that chromatin integrity is restored behind advancing RNAPII. These activities are known to prevent promiscuous transcription initiation events from cryptic promoters within open reading frames (Kaplan et al. 2003; Mason and Struhl 2003; Carrozza et al. 2005; Joshi and Struhl 2005; Keogh et al. 2005; Li et al. 2007; Cheung et al. 2008; Venkatesh and Workman 2013). ZCW1 also shows strong association with apparent orthologs of several SWR1/SRCAP/EP400 remodeling complex subunits (Willhoft and Wigley 2020; Scacchetti and Becker 2021), including Swr1/SRCAP (Tb927.11.10730), Swc6/ZNHIT1 (Tb927.11.6290), Swc2/YL1 (Tb927.11.5830), two RuvB-related helicases (Tb927.4.2000; Tb927.4.1270), actin and actin-related proteins (Tb927.4.980, Tb927.10.2000, Tb927.3.3020), and the possible equivalents of the Swc4/DMAP1 (Tb927.7.4040) and Yaf9/GAS41 YEATS domain protein (Tb927.10.11690, designated YEA1) subunits (Supplemental Tables S5, S6). Most of these putative TbSWR1-C subunits were also detected as being associated with BDF2 (Fig. 4A,B; Supplemental Tables S4, S5). Thus, because the yeast Bdf1 and human BRD8 Bromo domain proteins also associate with SWR1-C/SRCAP-C/EP400, it is likely that TbBDF2 performs a similar function in engaging acetylated histones. The SWR/SRCAP remodeling complexes are well known for being required to direct the replacement of H2A with H2A.Z in nucleosomes residing close to transcription start sites (Mizuguchi et al. 2004; Ruhl et al. 2006). The prevalence of both H2A.Z and H2B.V with affinity-selected ZCW1 suggests that it may also play a role in directing the *T. brucei* SWR/SRCAP complex to TSRs to ensure incorporation of H2A.Z-H2B.V in place of H2A-H2B in resident nucleosomes.

#### BDF6, EAF6, and HAT1

Multiple sequence alignments indicate that Tb927.1.3400 contains a Bromo domain (Supplemental Fig. S1C), and this protein was named BDF6. BDF6, EAF6, and HAT1 show robust association with each other; with a second YEATS domain protein, the possible ortholog of acetylated-H2A.Z binding Yaf9/Gas41 (Tb927.7.5310, designated YEA2); and with a set of five other uncharacterized proteins (Tb927.1.650, Tb927.6.1240, Tb927.8.5320, Tb927.10.14190, Tb927.11.3430) (Fig. 4A,B; Supplemental Table S4). Tb927.1.650 is an MRG domain protein with similarity to yeast Eaf3, whereas Tb927.10.14190 appears to be orthologous to yeast Epl1, both of which along with Yaf9 are components of the yeast NuA4 complex (Supplemental Tables S5, S7). The yeast Yaf9 YEATS protein contributes to both the NuA4 HAT and SWR1 complexes and can bind acetylated or crotonylated histone tails (Arrowsmith and Schapira 2019; Timmers 2020). In *T. brucei*, it appears that distinct YEATS domain proteins contribute to putative SWR1 (YEA1) and NuA4 (YEA2) complexes. HAT1 associates with both BDF6 and EAF6, whereas HAT3, EAF6, and PHD1 reciprocally associate with each other and share Tb927.11.7880, a YNG2-related ING domain protein, as a common interactor (Supplemental Fig. S8; Supplemental Tables S5, S7). Thus, HAT3–EAF6–PHD1–YNG2 perhaps represents a *T. brucei* subcomplex analogous to yeast piccolo-NuA4, whereas HAT1–EAF6–BDF6–EAF3–EPL1–YEA2 may form the larger NuA4 complex (Doyon and Côté 2004; Wang et al. 2018). In *T. brucei*, the Esa1/TIP60 catalytic MYST acetyltransferase function may be shared between HAT1 and HAT3. Although we have clearly identified NuA4-like complexes, no association was detected with a *T. brucei* Eaf1/EP400-related Helicase-SANT domain protein that provides the platform for the assembly of distinct modules of the yeast and metazoan NuA4 complexes (Levi et al. 1987; Scacchetti and Becker 2021).

Together, BDF6, EAF6, and HAT1 appear as part of a putative NuA4-related complex that functions at *T. brucei* TSRs. HAT1 has been shown to be required for H2A.Z and H2B.V acetylation and efficient RNAPII engagement and transcription (Kraus et al. 2020). BDF6, YEA2, or both may bind acetylated histones at TSRs to promote stable association of interacting chromatin modification and remodeling activities that enable the required histone dynamics to take place in these highly specialized regions and thereby facilitate efficient transcription.

#### DAC1/HDAC1

We also readily detect DAC1/HDAC1 enriched broadly over RNAPII TSRs, but notably, it does not associate with any of the other factors we found in these regions (Fig. 4A; Supplemental Table S4). However, five uncharacterized proteins (Tb927.3.890, Tb927.4.3730, Tb927.6.3170, Tb927.7.1650, Tb927.9.2070) reproducibly associated with DAC1/HDAC1. HHpred searches detected some similarity of Tb927.3.890, Tb927.4.3730, and Tb927.6.3170 to chromatin-associated proteins (Supplemental Table S5), and Tb927.9.2070 was also enriched with CRD1. DAC1/HDAC1 was previously shown to be an essential nuclear protein whose knockdown increases silencing of telomere-adjacent reporters in BF parasites (Wang et al. 2010). However, a general role for DAC1/HDAC1 at RNAPII TSRs was not anticipated. DAC1/HDAC1 has been reported to be mainly cytoplasmic in procyclic cells (Wang et al. 2010), and it is therefore expected to be absent from RNAPII TSRs in insect form parasites.

### Eight proteins are specifically enriched over RNAPII TTRs coincident with RNAPIII-transcribed genes

**Figure 5.**
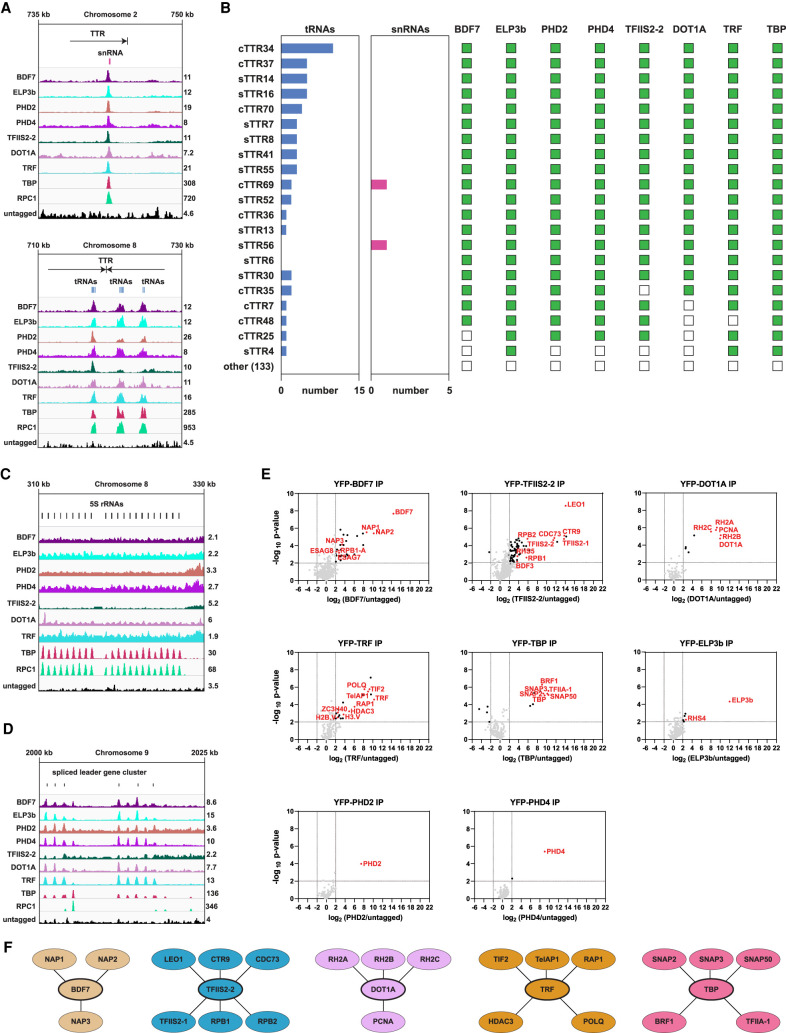
Proteins enriched over RNAPII TTRs coinciding with RNAPIII-transcribed genes define distinct interaction networks. The ChIP-seq tracks show a single replicate for each protein and are scaled separately as reads per million (values shown at the end of each track). (*A*) Examples of protein enrichment over an snRNA (*top* panel) and tRNAs (*bottom* panel). (*B*) Overlap between TTRs, tRNAs, snRNAs, and RNAPIII/TTR-associated factors. The numbers *under* the horizontal bar graphs refer to the number of tRNA or snRNA genes overlapping with each TTR. The presence and absence of overlap with RNAPIII/TTR-associated factors is indicated by green and empty squares, respectively. (cTTRs) Convergent TTRs, (sTTRs) single TTRs. (*C*) Enrichment of the RNAPIII/TTR-associated factors and RPC1 at the 5S rRNA gene cluster. (*D*) Enrichment of the RNAPIII/TTR-associated factors and RPC1 at the spliced leader gene cluster. (*E*) YFP-tagged proteins found to be enriched at a subset of TTRs were analyzed by LC-MS/MS to identify their protein interactions. The data for each plot are based on three biological replicates. Cutoffs used for significance: log_2_ (tagged/untagged) >2 or <−2 and *P* < 0.01 (Student's *t*-test). Enrichment scores for proteins identified in each affinity selection are presented in Supplemental Table S4. Significantly enriched proteins are indicated by black or colored dots. Proteins of interest are indicated by the red font. (*F*) Key proteins identified as being associated with the indicated YFP-tagged bait proteins (thick oval outlines). Lines denote interactions between proteins.

Our analysis of ChIP-seq association patterns also revealed a distinct set of eight proteins which displayed sharp peaks at a subset of RNAPII TTRs that coincide with RNAPIII-transcribed genes (Fig. 5; Supplemental Fig. S6B). These RNAPIII/TTR-associated factors included six of our candidate readers and writers (BDF7, ELP3b, PHD2, PHD4, TFIIS2-2, and DOT1A), as well as two of the selected control proteins (TRF and TBP). A total of 154 RNAPII TTRs have been annotated in the Lister 427 genome (Müller et al. 2018). We observed enrichment of some or all of these eight proteins at the 20 TTRs that overlap with tRNA and/or snRNA genes and also at the single TTR6 (sTTR6), which lacks such genes (Fig. 5A,B). We detected, consistent with *T. brucei* snRNA and tRNA genes being transcribed by RNAPIII (Nakaar et al. 1997; Tschudi and Ullu 2002), enrichment of RPC1 (the largest RNAPIII subunit) at these locations (Fig. 5A). However, although RPC1 associates with sTTR6, it is not enriched at any of the other 133 TTRs lacking annotated RNAPIII-transcribed genes. Our analysis suggests that an unannotated RNAPIII transcript is probably produced within sTTR6 but that the majority of RNAPII TTRs are not associated with RNAPIII transcription or with any of the eight highlighted proteins.

These eight RNAPIII/TTR-associated factors were also clearly enriched at the RNU2 (Tb927.2.5680) and U6 (Tb927.4.1213) snRNA genes and at 53 of the 69 tRNA genes annotated in the Lister 427 genome assembly (Fig. 5A,B; Supplemental Fig. S6B; Supplemental Table S8). We observed enrichment of only some of these proteins at 11 of the other tRNA genes and no association with the remaining five. Moreover, not all tRNA genes bound by these proteins coincide with an RNAPII TTR (Supplemental Fig. S6B; Supplemental Table S8).

RNAPIII/RPC1 was also enriched over the 5S rRNA cluster together with TBP but not the other seven members of this protein group (Fig. 5C). However, all eight proteins gave prominent peaks over the SL gene cluster (Fig. 5D). The 15 RNAPII promoter–associated factors discussed above also show sharp peaks that appear to coincide with those of the RNAPIII/TTR-associated factors at some locations (Supplemental Fig. S6C); the significance of this colocation is unknown.

It was unexpected that the terminal (TTAGGG)_n_ telomere repeat-binding protein TRF was one of the eight proteins enriched at internal chromosomal regions overlapping RNAPIII-associated TTRs. We hypothesized that enrichment of TRF at this subset of TTRs could result from the presence of underlying sequence motifs with similarity to canonical (TTAGGG)_n_ telomere repeats; however, sequence scrutiny revealed no significant matches. *T. brucei* contains approximately 115 linear chromosomes and, consequently, has an abundance of telomeres and telomere binding proteins that cluster at the nuclear periphery (Yang et al. 2009; DuBois et al. 2012; Akiyoshi and Gull 2013; Reis et al. 2018). The tethering of RNAPIII-transcribed genes to the nuclear periphery, as observed in yeast (Iwasaki et al. 2010; Chen and Gartenberg 2014), would bring them in close proximity to telomeres, offering a potential explanation for the association of TRF with these nucleosome depleted regions.

In yeasts, both the cohesin and condensin complexes, which shape chromosome architecture, are enriched or loaded at highly transcribed regions such as tRNA genes (D'Ambrosio et al. 2008; Haeusler et al. 2008; Gard et al. 2009; Iwasaki et al. 2010). Because the *T. brucei* Scc1 cohesin subunit is also enriched over tRNA genes (Müller et al. 2018), it is possible that the plethora of factors associated with RNAPIII-transcribed genes mediate the formation of nucleosome depleted boundary structures that facilitate transcription termination of polycistronic units by obstructing the passage of RNAPII. Because none of the eight proteins identified decorate RNAPII TSRs, they likely contribute to RNAPII transcription termination and/or facilitate RNAPIII transcription of tRNA and snRNA genes.

### Interaction networks of RNAPIII/TTR-associated proteins

To gain more insight into the functional context of the eight proteins found to be enriched at the subset of TTRs coinciding with RNAPIII-transcribed genes, we applied the same approach used to identify interacting partners of the promoter-associated factors. Below we detail the interaction networks of the RNAPIII/TTR-enriched proteins and discuss their potential functional implications (Fig. 5E,F; Supplemental Tables S4, S5).

Affinity selection of the telomere binding protein TRF resulted in enrichment of previously identified TRF- and telomere-associated proteins (Reis et al. 2018). The interaction of HDAC3 with TRF and BDF2 and its enrichment over TSRs suggest that HDAC3 functions at both RNAPII promoters and at subtelomeric regions (Fig. 5E,F). Our proteomic analyses detected no significant interactions of ELP3b, PHD2, and PHD4 with other proteins (Fig. 5E; Supplemental Table S4). Indeed, none of the eight RNAPIII/TTR-associated factors displayed reciprocal interactions with each other. This lack of cross talk suggests that each protein performs distinct functions at these locations.

#### BDF7 and NAP proteins

BDF7 is a Bromo domain protein containing an AAA^+^ ATPase domain, equivalent to Yta7, Abo1, and ATAD2 of budding and fission yeast and of vertebrates, respectively. These proteins have been implicated in altering nucleosome density to facilitate transcription, and Abo1 has recently been shown to mediate H3-H4 deposition onto DNA in vitro (Lombardi et al. 2011; Cho et al. 2019; Murawska and Ladurner 2020). Affinity-selected YFP-BDF7 showed strong association with three Nucleosome Assembly Proteins (Tb927.1.2210, designated NAP1; Tb927.3.4880, designated NAP2; and Tb927.10.15180, designated NAP3) (Fig. 5E,F; Supplemental Table S4), supporting a potential role for BDF7 as a histone chaperone involved in nucleosome formation. H3.V and H4.V tend to be enriched at the end of trypanosome polycistronic units where RNAPII transcription is terminated (Siegel et al. 2009), and we find that BDF7 is associated with a subset of these TTRs (Fig. 5B). Thus, it is possible that BDF7 acts with NAP1–3 to mediate H3.V-H4.V deposition at *T. brucei* RNAPIII-associated TTRs. Distinct nucleosome-depleted regions are formed over tRNA genes, and as discussed above, the termination of some RNAPII transcription occurs in regions coincident with RNAPIII-transcribed genes (Marchetti et al. 1998; Siegel et al. 2009; Maree and Patterton 2014). As suggested previously, it is possible that a subset of genes transcribed by RNAPIII acts as boundaries that block the passage of advancing RNAPII into convergent or downstream transcription units (Siegel et al. 2009). BDF7 may act with associated NAP proteins to promote nucleosome depletion and termination over such regions.

#### TFIIS2-2 and the PAF1 complex

TFIIS2-2 was clearly enriched in the vicinity of RNAPIII-transcribed genes and associated TTRs and, upon affinity selection, showed strong interaction with PAF1 complex components (LEO1/Tb927.9.12900, CTR9/Tb927.3.3220, CDC73/Tb927.11.10230) and with several RNAPII subunits (Fig. 5E,F; Supplemental Table S4). Yeast Paf1C acts with TFIIS to enable transcription elongation through chromatin templates (Van Oss et al. 2017; Schier and Taatjes 2020). The accumulation of PAF1 complex subunits at these TTR regions presumably reflects the role that these proteins are known to play in transcription termination and 3′ end processing of RNAPII transcripts.

#### DOT1A and the RNase H2 complex

The DOT1A and DOT1B HMTs direct trypanosome H3K76 di- and trimethylation, respectively (Janzen et al. 2006b). DOT1A is involved in cell cycle progression, whereas DOT1B is necessary for maintaining the silent state of inactive VSGs and for rapid transcriptional VSG switching (Janzen et al. 2006b; Figueiredo et al. 2008). Our proteomic analysis revealed that DOT1A associates with all three subunits of the RNase H2 complex (RH2A, RH2B, and RH2C) (Fig. 5E,F; Supplemental Table S4). The RH1 and RH2 complexes are necessary for resolving R-loops formed during transcription (Cerritelli and Crouch 2009). Although both RH1 and RH2 complexes are involved in antigenic variation, only RH2 has a role in trypanosome RNAPII transcription (Briggs et al. 2019). A recent study suggests that DOT1B is also required to clear R-loops by suppressing RNA–DNA hybrid formation and resulting DNA damage (Eisenhuth et al. 2020). DOT1A, which we find to be enriched at tRNA and snRNA genes (see above), may act with RH2 to prevent the accumulation of RNA–DNA hybrids at these RNAPIII-transcribed regions.

#### TBP, BRF1, and RNAPIII-transcribed genes

TATA-box related protein (TBP) has largely been studied with respect to its role in RNAPII transcription from SL RNA gene promoters (Das et al. 2005). However, TBP was also previously shown to associate strongly with the TFIIIB component BRF1 (Schimanski et al. 2005), and thus, like BRF1, TBP may also promote RNAPIII transcription (Vélez-Ramírez et al. 2015). Indeed, directed ChIP assays indicate that TBP associates with specific RNAPIII-transcribed genes (Ruan et al. 2004; Vélez-Ramírez et al. 2015). We confirmed that, consistent with a dual role, YFP–TBP interacts with both SNAP complex components involved in SL transcription and with BRF1 (Fig. 5E,F; Supplemental Table S4). Our ChIP-seq analysis shows that TBP is concentrated in sharp peaks that coincide with both RNAPII promoters for SL RNA genes as well as RNAPIII-transcribed tRNA and snRNA genes (Fig. 5A,D). Additionally, TBP was significantly enriched at arrays of RNAPIII-transcribed 5S rRNA genes (Fig. 5C). Thus, apart from SL RNA gene promoters, TBP appears to mark all known sites of RNAPIII-directed transcription.

## Conclusions

**Figure 6.**
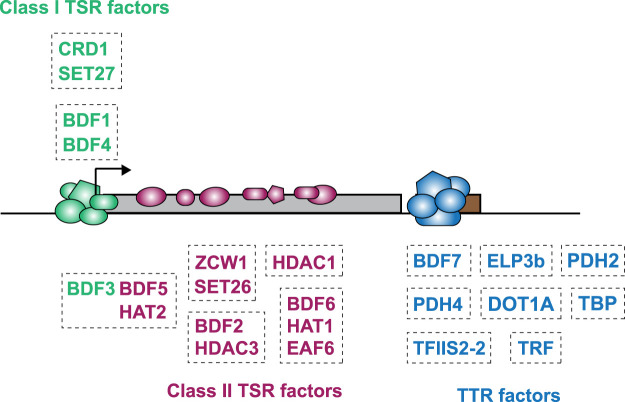
Model depicting distribution of chromatin regulators across a trypanosome polycistronic transcription unit. Diagram shows the five Class I (sharp; green) and 10 Class II (broad; purple) TSR-associated factors at a unidirectional/single RNAPII promoter. The arrow indicates the direction of transcription. The gray rectangle represents a single polycistron. Class II proteins are enriched in the direction of transcription. The eight proteins found at RNAPII TTRs associated with RNAPIII-transcribed genes are shown in blue. tRNA and snRNA genes are represented by the brown rectangle. Proteins within each box were found to interact in the proteomic experiments.

Using protein domain homology searches, we identified a collection of 65 putative chromatin regulators in *T. brucei* that were predicted to act as writers, readers, or erasers of histone PTMs. Many of these proteins showed a discernible nuclear localization and displayed distinct patterns of association across the genome, frequently coinciding with regions responsible for RNAPII transcription initiation and termination or RNAPIII transcription (Fig. 6). Robust proteomic analyses allowed the interaction networks of these proteins to be identified, thereby providing further insight into their possible functions at specific genomic locations by revealing putative complexes likely involved in the distinct phases of transcription: initiation, elongation, and termination. Counterparts of yeast SWR1 H2A-to-H2A.Z exchange complex and NuA4 HAT complex components were identified, some of which were enriched where H2A.Z is prevalent at TSRs. A recent report indicates that both TbSWR1 and TbNuA4 complexes are indeed involved in the deposition of *T. brucei* H2A.Z (Vellmer et al. 2021).

We also show that two predicted SET domain methyltransferases associate with putative histone modification reader proteins with which they occupy RNAPII TSRs. Moreover, our analyses reveal that six of the seven Bromo domain proteins are involved in four interaction networks enriched at TSRs, whereas the BDF7 network alone marks a subset of TTRs that is coincident with sites of RNAPIII transcription. The association of SET and Bromo domain proteins with conserved RNAPII subunits, histone acetyltransferases, chromatin chaperones, and remodeling factors suggests that the networks identified play pivotal roles in defining sites of transcription initiation and termination.

The data presented here provide a comprehensive depiction of the operational context of chromatin writers, readers, and erasers at important genomic regulatory elements in this experimentally tractable but divergent eukaryote. Critically, our analyses identify many novel proteins unrelated to, or divergent from, known chromatin regulators of conventional eukaryotes. This highlights the utility of our approach to reveal novelty in the composition of *T. brucei* chromatin regulatory complexes, which differ from the paradigms established using conventional eukaryotic models.

Trypanosome gene expression is generally considered to be regulated post-transcriptionally with a plethora of factors dedicated to sculpting mature mRNAs from nascent polycistronic transcripts (Clayton 2019). The complexity of chromatin regulatory factors that we have found to be enriched at TSRs and some TTRs may simply represent the core set of proteins required to mediate efficient and constitutive eukaryotic transcription initiation, elongation, and termination in a chromatin context. An alternative possibility is that these proteins, with potentially antagonistic functions, operate in a more complex regulatory landscape where transcriptional control contributes alongside post-transcriptional mechanisms in ensuring optimal trypanosome gene expression.

## Methods

### Cell culture

Lister 427 BF *T. brucei* was used throughout this study. Parasites were grown in HMI-9 medium (Hirumi and Hirumi 1989) at 37°C and 5% CO_2_. Cell lines with YFP-tagged proteins were grown in the presence of 5 µg/mL blasticidin. The density of cell cultures was maintained below 3 × 10^6^ cells/mL.

### Protein tagging

Candidate proteins were tagged endogenously on their N termini with YFP using the pPOTv4 plasmid (Dean et al. 2015). Tagging constructs were produced by fusion PCR of three fragments: a ∼500-bp fragment homologous to the end of the 5′ UTR of each gene, a region of the pPOTv4 plasmid containing a blasticidin-resistance cassette and a YFP tag, and a ∼500-bp fragment homologous to the beginning of the coding sequence of each gene. Supplemental Table S9 lists the primers used for tagging. Fusion constructs were transfected into BF parasites by electroporation as previously described (Burkard et al. 2007). The cell lines obtained after blasticidin selection were tested for correct integration of the tagging constructs by PCR and for expression of the tagged proteins via western blotting analysis.

### Fluorescent immunolocalization

Cells were fixed with 4% paraformaldehyde for 10 min on ice. Fixation was stopped with 0.1 M glycine. Cells were added to polylysine-coated slides and permeabilized with 0.1% Triton X-100. The slides were blocked with 2% BSA. Rabbit anti-GFP primary antibody (Thermo Fisher Scientific A-11122) was used at 1:500 dilution, and secondary Alexa fluor 568 goat antirabbit antibody (Thermo Fisher Scientific A-11036) was used at 1:1000 dilution. Images were taken with a Zeiss Axio Imager microscope.

### Chromatin immunoprecipitation and sequencing

Parasites, 4 × 10^8^, were fixed with 0.8% formaldehyde for 20 min at room temperature. Cells were lysed and sonicated in the presence of 0.2% SDS for 30 cycles (30 sec on, 30 sec off) using the high setting on a Bioruptor sonicator (Diagenode). Cell debris were pelleted by centrifugation, and SDS in the lysate supernatants was diluted to 0.07%. Input samples were taken before incubating the rest of the cell lysates overnight with 10 µg rabbit anti-GFP antibody (Thermo Fisher Scientific A-11122) and Protein G Dynabeads. The beads were washed, and the DNA eluted from them was treated with RNase and Proteinase K. DNA was then purified using a QIAquick PCR purification kit (Qiagen), and libraries were prepared using NEXTflex barcoded adapters (Bioo Scientific). The libraries were sequenced on Illumina HiSeq 4000 (Edinburgh Genomics), Illumina NextSeq (Western General Hospital, Edinburgh), or Illumina MiniSeq (Allshire laboratory). In all cases, 75-bp paired-end sequencing was performed. Our subsequent analyses were based on three replicates for BDF6, CRD1, and DMT; one replicate for ELP3a, NAT2, and SET10; and two replicates for the remaining YFP-tagged proteins and for the untagged control.

### ChIP-seq data analysis

Sequencing reads were deduplicated with pyFastqDuplicateRemover (Webb et al. 2014; https://git.ecdf.ed.ac.uk/sgrannem/pycrac) and subsequently aligned to the Tb427v9.2 genome (Müller et al. 2018) with Bowtie 2 (Langmead and Salzberg 2012). The default mode of Bowtie 2 was used, which searches for multiple alignments and reports the best one or, if several alignments are deemed equally good, reports one of those randomly. The ChIP samples were normalized to their respective inputs (ratio of ChIP to input reads) and to library size (reads per million). TSR and TTR regions were defined based on annotations of the Lister 427 genome (Müller et al. 2018). CRD1 peak summits were called using the narrow peak mode of MACS2 (Feng et al., 2012) followed by manual filtering of false positives, which included peaks not present in all CRD1 replicates, peaks present in the untagged control, and/or peaks with fold enrichment < 6.5. Ten-kilobase regions upstream of and downstream from CRD1 peak summits were divided into 50-bp windows. The metagene plots display individual ChIP-seq replicates separately and were generated by summing normalized reads in each 50-bp window and representing them as density centered around CRD1. The average metagene plots were generated analogously, except that the reads around all CRD1 peaks were averaged before plotting. Heatmaps represent normalized reads around individual CRD1 peaks and were generated as an average of all replicates for each protein.

### Affinity purification and LC-MS/MS proteomic analysis

Cells, 4 × 10^8^, were lysed per IP in the presence of 0.2% NP-40 and 150 mM KCl. Lysates were sonicated briefly (three cycles, 12 sec on, 12 sec off) at a high setting in a Bioruptor (Diagenode) sonicator. The soluble and insoluble fractions were separated by centrifugation, and the soluble fraction was incubated for 1 h at 4°C with beads cross-linked to mouse anti-GFP antibody (Roche 11814460001). The resulting immunoprecipitates were washed three times with lysis buffer, and protein was eluted with RapiGest surfactant (Waters) for 15 min at 55°C. Next, filter-aided sample preparation (FASP) (Wiśniewski et al. 2009) was used to digest the protein samples for mass spectrometric analysis. Briefly, proteins were reduced with DTT and then denatured with 8 M urea in Vivakon spin (filter) column 30K cartridges. Samples were alkylated with 0.05 M IAA and digested with 0.5 μg MS grade Pierce trypsin protease (Thermo Fisher Scientific) overnight, desalted using stage tips (Rappsilber et al. 2007), and resuspended in 0.1% TFA for LC-MS/MS. Peptides were separated using RSLC Ultimate 3000 system (Thermo Fisher Scientific) fitted with an EasySpray column (50 cm; Thermo Fisher Scientific) using 2%–40%–95% nonlinear gradients with solvent A (0.1% formic acid) and solvent B (80% acetonitrile in 0.1% formic acid). The EasySpray column was directly coupled to an Orbitrap Fusion Lumos (Thermo Fisher Scientific) operated in DDA mode. “TopSpeed” mode was used with 3-sec cycles with standard settings to maximize identification rates: MS1 scan range 350–1500 mz, RF lens 30%, AGC target 4.0e^5^ with intensity threshold 5.0e^3^, filling time 50 msec and resolution 60,000, monoisotopic precursor selection, and filter for charge states two through five. HCD (27%) was selected as fragmentation mode. MS2 scans were performed using an ion trap mass analyzer operated in rapid mode with AGC set to 2.0e^4^ and filling time to 50 msec. The resulting shot-gun data were processed using Maxquant 1.6.1.0 (*T. brucei* proteome from May 14, 2019) and visualized using Perseus 1.6.1.3 (Tyanova et al. 2016).

## Data access

All raw and processed sequencing data generated in this study have been submitted to the NCBI Gene Expression Omnibus (GEO; https://www.ncbi.nlm.nih.gov/geo/) under accession number GSE150253. The proteomics data generated in this study have been submitted to the Proteomics Identifications Database (PRIDE; https://www.ebi.ac.uk/pride/) under accession number PXD026743.

## Supplementary Material

Supplemental Material
