# Supplementary material for: A systematic analysis of Trypanosoma brucei chromatin factors identifies novel protein interaction networks associated with sites of transcription initiation and termination
Source: Genome Res. 2021 Nov;31(11):2138–54. doi: 10.1101/gr.275368.121 (PMC8559703; doi:10.1101/gr.275368.121)
Supplement: Supplemental Material [file supp_31_11_2138__DC1.html]

A systematic analysis of Trypanosoma brucei chromatin factors identifies novel protein interaction networks associated with sites of transcription initiation and termination — Supplemental Material 

# A systematic analysis of *Trypanosoma brucei* chromatin factors identifies novel protein interaction networks associated with sites of transcription initiation and termination

## Supplemental Material

- Supplemental\_Tables.pdf
- Supplemental\_Figures.pdf
