# Supplementary material for: A systematic analysis of Trypanosoma brucei chromatin factors identifies novel protein interaction networks associated with sites of transcription initiation and termination
Source: Genome Res. 2021 Nov;31(11):2138–54. doi: 10.1101/gr.275368.121 (PMC8559703; doi:10.1101/gr.275368.121)
Supplement: Supplemental Material [file supp_gr.275368.121_Supplemental_Tables_with_references.pdf]

Supplemental Tables for

A systematic analysis of *Trypanosoma brucei* chromatin factors identifies novel protein interaction networks associated with sites of transcription initiation and termination

Desislava P. Staneva*, Roberta Carloni*, Tatsiana Auchynnikava Pin Tong, Juri Rappsilber, A. Arockia Jeyaprakash, Keith R. Matthews† and Robin C. Allshire†

* These authors contributed equally to this work.

† Co-corresponding authors:

Robin Allshire: robin.allshire@ed.ac.uk

Keith Matthews: keith.matthews@ed.ac.uk

Contents

Page

1 Supplemental Table S1. Protein localization in bloodstream and procyclic form cells

6 Supplemental Table S2. Functions and phenotypes of previously characterized candidate proteins

8 Supplemental Table S3. Candidate proteins with no specific genomic enrichment by ChIP-seq

10 Supplemental Table S4. Significantly enriched interactors detected by proteomic analysis of affinity selected YFP-tagged proteins

11 CRD1 IP significantly enriched interactors (over untagged control)

12 SET27 IP significantly enriched interactors (over untagged control)

13 BDF1 IP significantly enriched interactors (over untagged control)

14 BDF4 IP significantly enriched interactors (over untagged control)

15 BDF3 IP significantly enriched interactors (over untagged control)

16 BDF5 IP significantly enriched interactors (over untagged control)

17 HAT2 IP significantly enriched interactors (over untagged control)

18 BDF2 IP significantly enriched interactors (over untagged control)

20 HDAC3 IP significantly enriched interactors (over untagged control)

21 SET26 IP significantly enriched interactors (over untagged control)

23 ZCW1 IP significantly enriched interactors (over untagged control)

25 BDF6 IP significantly enriched interactors (over untagged control)

26 HAT1 IP significantly enriched interactors (over untagged control)

27 EAF6 IP significantly enriched interactors (over untagged control)

28 HDAC1 IP significantly enriched interactors (over untagged control)

29 BDF7 IP significantly enriched interactors (over untagged control)

30 TFIIS2-2 IP significantly enriched interactors (over untagged control)

DOT1A IP significantly enriched interactors (over untagged control)

TRF IP significantly enriched interactors (over untagged control)

TBP IP significantly enriched interactors (over untagged control)

ELP3b IP significantly enriched interactors (over untagged control)

PHD2 IP significantly enriched interactors (over untagged control)

PHD4 IP significantly enriched interactors (over untagged control)

PHD1 IP significantly enriched interactors (over untagged control)

PHD5 IP significantly enriched interactors (over untagged control)

HAT3 IP significantly enriched interactors (over untagged control)

AGO1 IP significantly enriched interactors (over untagged control)

NUP110 IP significantly enriched interactors (over untagged control)

SET13 IP significantly enriched interactors (over untagged control)

SET15 IP significantly enriched interactors (over untagged control)

SET20 IP significantly enriched interactors (over untagged control)

SET23 IP significantly enriched interactors (over untagged control)

SET25 IP significantly enriched interactors (over untagged control)

Supplemental Table S5. Information about key proteins enriched with putative chromatin regulators in *T. brucei*

CRD1-SET27 network

Enriched in YFP-CRD1 affinity selections

Enriched in YFP-SET27 affinity selections

BDF1-BDF4 network

Enriched in YFP-BDF1 affinity selections

Enriched in YFP-BDF4 affinity selections

BDF3-BDF5-HAT2 network

Enriched in YFP-BDF3 affinity selections

Enriched in YFP-BDF5 affinity selections

Enriched in YFP-HAT2 affinity selections

BDF2-HDAC3 network

Enriched in YFP-BDF2 affinity selections

Enriched in YFP-HDAC3 affinity selections

SET26-ZCW1 network

Enriched in YFP-SET26 affinity selections

Enriched in YFP-ZCW1 affinity selections

BDF6-EAF6-HAT1 network

Enriched in YFP-BDF6 affinity selections

Enriched in YFP-HAT1 affinity selections

Enriched in YFP-EAF6 affinity selections

Enriched in YFP-HDAC1 affinity selections

Enriched in YFP-BDF7 affinity selections (top interactors)

Enriched in YFP-TFIIS2-2 affinity selections (top interactors)

Enriched in YFP-DOT1A affinity selections

Enriched in YFP-TRF affinity selections (top interactors)

Enriched in YFP-TBP affinity selections

Enriched in YFP-ELP3b affinity selections

Enriched in YFP-PHD2 affinity selections

Enriched in YFP-PHD4 affinity selections

Enriched in YFP-PHD1 affinity selections

Enriched in YFP-PHD5 affinity selections

Enriched in YFP-HAT3 affinity selections

Enriched in YFP-AGO1 affinity selections

Enriched in YFP-NUP110 affinity selections

Frequent non-specific contaminants

Supplemental Table S6. *T. brucei* homologs of SWR1-C/SRCAP-C/EP400 subunits

102 Supplemental Table S7. *T. brucei* homologs of NuA4 subunits

104 Supplemental Table S8. Overlap of tRNA and snRNA genes annotated in the assembled Lister 427 genome with TTRs and RNAPIII/TTR-associated factors

108 Supplemental Table S9. Primers used for N-terminal tagging

118 References

Supplemental Table S1

Supplemental Table S1. Protein localization in bloodstream and procyclic form cells

[1] RIT-seq data from Alsford et al (2011). Scoring matrix key: 0-0-0-0 group - significant loss of fitness in all four experiments; 0-0-1-0 group - significant loss of fitness in BF experiments only; 1-1-0-0 group - significant loss of fitness in PF experiments only; 1-1-1-0 group - significant loss of fitness in the differentiation (BF to PF) experiment only; 1-1-1-1 group - no significant loss of fitness in any experiment; none of the above - other groups.

[2] Data from www.tryptag.org was accessed on 12 June 2021.

* PHD3 is a shorter version of PHD1 with a single amino acid change apart from the truncation. PHD3 lacks a UniProt ID and may be a misannotation of PHD1.

** ELP3c is a shorter version of ELP3b with a single amino acid change apart from the truncation. ELP3c lacks a UniProt ID and may be a misannotation of ELP3b.

*** SET7 is a shorter version of SET1. SET7 lacks a UniProt ID and may be a misannotation of SET1.

SET2 (Tb11.v5.0590) and SET19 (Tb927.7.5620) were found to be identical, thus only SET19 is included in this dataset.

Protein name	TriTryp ID	UniProt ID	Protein domain/family	[1]RIT-seq scoring matrix	Localization in this study (BF)	[2]TrypTag localization (PF)
BDF1	Tb927.10.8150	Q38AE9	Bromo	1-1-1-1	predominantly nuclear	N-terminal tag: nuclear lumen;<br>C-terminal tag: nuclear, cytoplasmic
BDF2	Tb927.10.7420	Q38AM1	Bromo	1-1-1-1	nuclear	N-terminal tag: nuclear
BDF3	Tb927.11.10070	Q383S2	Bromo	other	predominantly nuclear	N-terminal tag and C-terminal tag: nucleoplasm
BDF4	Tb927.7.4380	Q57UR8	Bromo	other	nuclear and cytoplasmic	N-terminal tag and C-terminal tag: nuclear
BDF5	Tb927.11.13400	Q382J7	Bromo	0-0-1-0	nuclear	N-terminal tag: nucleoplasm
BDF6	Tb927.1.3400	Q4GYN3	Bromo	other	nuclear	N-terminal tag: nucleoplasm;<br>C-terminal tag: nucleoplasm, cytoplasmic
BDF7	Tb927.11.6350	Q385D4	Bromo	1-1-1-1	nuclear	N-terminal tag: nuclear;<br>C-terminal tag: nucleoplasm
PHD1	Tb927.10.9930	Q389Y3	PHD	1-1-1-1	nuclear	N-terminal tag: nuclear lumen;<br>C-terminal tag: nucleoplasm
PHD2	Tb11.v5.0388	Q387D3	PHD	other	nuclear	N-terminal tag and C-terminal tag: nuclear lumen
PHD3*	Tb11.v5.0794	-	PHD	-	tagging unsuccessful	-
PHD4	Tb927.3.2140	Q57Z97	PHD	1-1-1-0	nuclear	C-terminal tag: nucleoplasm, cytoplasmic
PHD5	Tb927.11.5870	Q385I1	PHD and GNAT	1-1-1-1	nuclear and cytoplasmic	N-terminal tag and C-terminal tag: nuclear and cytoplasmic
TDR1	Tb927.11.14190	Q382B8	Tudor	1-1-1-1	cytoplasmic	N-terminal tag: cytoplasmic, flagellar cytoplasm, nuclear lumen
CRD1	Tb11.v5.0267	Q57X70	Chromo	1-1-1-1	nuclear	N-terminal tag: nuclear and cytoplasmic
TFIIS2-2	Tb927.2.3480	Q586Y0	PWWP	other	nuclear	N-terminal tag and C-terminal tag: nucleoplasm
ZCW1	Tb927.10.11720	Q389G9	Znf-CW	other	nuclear	N-terminal tag and C-terminal tag: nucleoplasm

Protein name	TriTryp ID	UniProt ID	Protein domain/family	[1]RIT-seq scoring matrix	Localization in this study (BF)	[2]TrypTag localization (PF)
EAF6	Tb927.9.2910	Q38FN2	NuA4 complex component	1-1-1-1	predominantly nuclear	N-terminal tag: nucleoplasm;<br>C-terminal tag: nuclear lumen
HAT1	Tb927.7.4560	D6XIY7	MYST	1-1-1-1	nuclear	N-terminal tag and C-terminal tag: nucleoplasm
HAT2	Tb927.11.11530	Q383C5	MYST	1-1-1-1	nuclear	N-terminal tag and C-terminal tag: nucleoplasm
HAT3	Tb927.10.8310	Q38AD3	MYST	0-0-0-0	predominantly nuclear	N-terminal tag: nuclear;<br>C-terminal tag: nuclear, cytoplasmic, flagellar cytoplasm
ELP3a	Tb927.8.5770	Q57X05	GNAT	1-1-1-1	nuclear periphery and cytoplasmic	N-terminal tag: nuclear envelope;<br>C-terminal tag: nuclear, cytoplasmic
ELP3b	Tb927.8.3310	Q57YY2	GNAT	other	nuclear and cytoplasmic	N-terminal tag: nuclear, spindle;<br>C-terminal tag: nuclear lumen
ELP3c**	Tb11.v5.0520	-	GNAT	-	tagging unsuccessful	-
NAT2	Tb927.1.4490	Q4GYC9	GNAT	1-1-1-0	predominantly cytoplasmic	C-terminal tag: cytoplasmic
NAT3	Tb927.10.12830	Q388W3	GNAT	1-1-1-1	predominantly cytoplasmic	N-terminal tag and C-terminal tag: cytoplasmic
LPLAT1	Tb927.5.2280	Q582D7	LPLAT	1-1-0-0	nuclear and cytoplasmic	C-terminal tag: endoplasmic reticulum, pellicular membrane
SET1	Tb11.v5.0422	Q38BE1	SET	1-1-1-1	predominantly cytoplasmic	-
SET3	Tb927.1.4720	Q4GYA6	SET	1-1-1-1	cytoplasmic	C-terminal tag: cytoplasmic
SET4	Tb927.10.11130	Q389M6	SET	1-1-1-1	cytoplasmic	N-terminal tag and C-terminal tag: cytoplasmic
SET5	Tb927.10.12880	Q388V8	SET	1-1-1-1	cytoplasmic	N-terminal tag: cytoplasmic;<br>C-terminal tag: mitochondrion, kinetoplast
SET6	Tb927.10.3730	Q38BM6	SET	other	predominantly cytoplasmic	N-terminal tag: cytoplasmic
SET7***	Tb927.10.4600	-	SET	-	predominantly cytoplasmic	N-terminal tag and C-terminal tag: paraflagellar rod, cytoplasmic
SET8	Tb927.10.8060	Q38AF8	SET	0-0-1-0	cytoplasmic	N-terminal tag: cytoplasmic, flagellar cytoplasm;<br>C-terminal tag: cytoplasmic
SET9	Tb927.10.9680	Q38A07	SET	1-1-1-1	cytoplasmic	N-terminal tag: cytoplasmic, flagellar cytoplasm, nuclear lumen
SET10	Tb927.11.13560	Q382I1	SET	0-0-0-0	nuclear and cytoplasmic	-
SET11	Tb927.11.5120	Q385Q6	SET	1-1-1-1	predominantly cytoplasmic	-
SET12	Tb927.3.750	Q57WV8	SET	other	protein not detected by western analysis	N-terminal tag: endocytic, cytoplasmic
SET13	Tb927.4.2440	Q584E3	SET	other	predominantly cytoplasmic	N-terminal tag: nucleolar, nuclear

Protein name	TriTryp ID	UniProt ID	Protein domain/family	[1]RIT-seq scoring matrix	Localization in this study (BF)	[2]TrypTag localization (PF)
SET14	Tb927.4.3310	Q584A8	SET	other	cytoplasmic	N-terminal tag: cytoplasmic, nucleoplasm; C-terminal tag: cytoplasmic, flagellum, nucleoplasm
SET15	Tb927.5.2770	Q57ZV3	SET	other	predominantly cytoplasmic	N-terminal tag and C-terminal tag: cytoplasmic, flagellar cytoplasm, nuclear, nucleolar
SET16	Tb927.5.3500	Q57UB6	SET	1-1-0-0	nuclear and cytoplasmic	N-terminal tag: cytoplasmic; C-terminal tag: cytoplasmic, flagellar cytoplasm, nuclear lumen
SET17	Tb927.6.3610	Q585V9	SET	other	nuclear and cytoplasmic	-
SET18	Tb927.6.910	Q585F5	SET	1-1-1-1	cytoplasmic	N-terminal tag and C-terminal tag: mitochondrion
SET19	Tb927.7.5620	Q582H7	SET	1-1-1-1	cytoplasmic	N-terminal tag: cytoplasmic; C-terminal tag: mitochondrion, kinetoplast
SET20	Tb927.8.2490	Q57XE0	SET	1-1-1-1	nuclear and cytoplasmic	N-terminal tag: cytoplasmic, flagellar cytoplasm, nuclear lumen; C-terminal tag: mitochondrion
SET21	Tb927.8.2690	Q57XC0	SET	1-1-1-1	cytoplasmic	N-terminal tag: cytoplasmic; C-terminal tag: nucleoplasm, cytoplasmic, flagellar cytoplasm
SET22	Tb927.8.2710	Q57XB8	SET	1-1-1-1	cytoplasmic	C-terminal tag: cytoplasmic
SET23	Tb927.8.6470	Q57YP8	SET	1-1-1-1	nuclear and cytoplasmic	N-terminal tag: cytoplasmic, flagellar cytoplasm, nuclear lumen
SET24	Tb927.8.6530	Q57TW0	SET	1-1-1-1	cytoplasmic	N-terminal tag: cytoplasmic; C-terminal tag: kinetoplast, mitochondrion
SET25	Tb927.9.1510	Q38FZ5	SET	other	nuclear and cytoplasmic	N-terminal tag and C-terminal tag: cytoplasmic
SET26	Tb927.10.8100	Q38AF4	SET	1-1-1-1	nuclear and cytoplasmic	N-terminal tag and C-terminal tag: nucleoplasm, cytoplasmic
SET27	Tb927.9.13470	Q38D80	SET	1-1-1-1	nuclear and cytoplasmic	N-terminal tag and C-terminal tag: nucleoplasm
SET28	Tb927.9.10100	Q38DZ4	SET	1-1-1-1	cytoplasmic	N-terminal tag: cytoplasmic
SET29	Tb927.3.3370	Q57V49	SET	other	cytoplasmic	N-terminal tag: cytoplasmic; C-terminal tag: cytoplasmic, flagellar cytoplasm, nuclear lumen
SET30	Tb927.3.1860	Q57ZC5	SET	1-1-1-0	protein not detected by western analysis	N-terminal tag: cytoplasmic; C-terminal tag: kinetoplast, mitochondrion
DOT1A	Tb927.8.1920	Q581Z0	DOT	1-1-1-1	predominantly nuclear	C-terminal tag: nuclear lumen
DOT1B	Tb927.1.570	Q4GZF2	DOT	1-1-1-1	nuclear and cytoplasmic	N-terminal tag: nuclear lumen; C-terminal tag: nuclear lumen, cytoplasmic
DOT1	Tb927.11.3200	Q386J1	DOT	1-1-1-1	protein not detected by western analysis	-

Protein name	TriTryp ID	UniProt ID	Protein domain/family	[1]RIT-seq scoring matrix	Localization in this study (BF)	[2]TrypTag localization (PF)
HDAC1	Tb927.10.1680	Q38C74	Class I HDAC	other	nuclear and cytoplasmic	C-terminal tag: cytoplasmic, flagellar cytoplasm, nuclear lumen
HDAC2	Tb927.11.15600	Q381M6	Class I HDAC	1-1-1-1	cytoplasmic	N-terminal tag: cytoplasmic, flagellar cytoplasm, nuclear lumen;<br>C-terminal tag: cytoplasmic
HDAC3	Tb927.2.2190	Q586J9	Class II HDAC	0-0-0-0	predominantly nuclear	N-terminal tag: nucleolar, nucleoplasm;<br>C-terminal tag: nucleoplasm
HDAC4	Tb927.5.2900	D6XGM6	Class II HDAC	1-1-1-0	cytoplasmic	-
Sir2rp1	Tb927.7.1690	Q57V41	Sir2 HDAC	1-1-1-1	predominantly cytoplasmic	N-terminal tag: cytoplasmic;<br>C-terminal tag: cytoplasmic, flagellar cytoplasm, nuclear lumen
Sir2rp2	Tb927.8.3140	Q57YZ9	Sir2 HDAC	1-1-1-1	cytoplasmic	-
Sir2rp3	Tb927.4.2520	Q584D5	Sir2 HDAC	0-0-0-0	nuclear and cytoplasmic	N-terminal tag: cytoplasmic, flagellar cytoplasm, nuclear lumen;<br>C-terminal tag: mitochondrion
JMJ1	Tb927.11.1760	Q386X8	JmjC	other	cytoplasmic	N-terminal tag: cytoplasmic;<br>C-terminal tag: cytoplasmic, flagellar cytoplasm, nucleoplasm
JMJ2	Tb927.11.2000	Q386V9	JmjC	0-0-0-0	predominantly nuclear	N-terminal tag and C-terminal tag: nuclear lumen
LCM1	Tb927.9.12780	Q38DD6	JmjC	1-1-1-1	nuclear and cytoplasmic	N-terminal tag: cytoplasmic
CLD1	Tb927.7.660	Q57VR2	JmjC	1-1-1-1	predominantly cytoplasmic	C-terminal tag: cytoplasmic, flagellar cytoplasm
AGO1	Tb927.10.10850	Q389P5	PAZ and PIWI	1-1-1-1	cytoplasmic	C-terminal tag: cytoplasmic
DMT	Tb927.3.1360	Q57XW9	DNMT	1-1-1-1	predominantly cytoplasmic	N-terminal tag: cytoplasmic;<br>C-terminal tag: cytoplasmic, flagellar cytoplasm
KKT2	Tb927.11.10520	Q383M7	Protein kinase	0-0-1-0	nuclear dots and cytoplasmic	C-terminal tag: nucleoplasm, cytoplasmic, kinetochore
NOC1	Tb927.11.2120	Q386U7	CBF/Mak21	0-0-1-0	nucleolar and cytoplasmic	N-terminal tag and C-terminal tag: nucleolar
NUP110/ MLP1	Tb927.11.330	Q387L8	SMC	0-0-1-0	nuclear pores and cytoplasmic	N-terminal tag: nuclear pores;<br>C-terminal tag: cytoplasmic
TRF	Tb927.10.12850	Q388W1	Patched	0-0-0-0	nuclear dots	N-terminal tag and C-terminal tag: nucleoplasm
TBP/TRF4	Tb927.10.15950	Q387R9	TATA binding	other	nuclear and cytoplasmic	N-terminal tag and C-terminal tag: nucleoplasm and cytoplasmic

Supplemental Table S2

Supplemental Table S2. Functions and phenotypes of previously characterized candidate proteins

Protein names	Functions/phenotypes	References
BDFs 1,3 and 4	enriched around RNAPII TSRs	Schulz et al., 2015
BDFs 2 and 3	required for maintenance of monoallelic VSG expression	Schulz et al., 2015
BDF5	essential in BF and PF cells	Alsford and Horn, 2012
TFIIS2-2	binds histone peptides bearing H4K17me3 or H3K32me3	Wang et al., 2019
HAT1	essential in BF cells; modulates telomeric silencing;<br>required for DNA replication, growth and RNA transcription;<br>acetylates histone variants H2A.Z and H2B.V at TSRs	Kawahara et al., 2008;<br>Kraus et al., 2020
HAT2	essential in BF cells; responsible for writing the H4K10ac mark found at RNAPII TSRs;<br>required for H2A.Z deposition; acetylates histone H4 at TSRs	Kawahara et al., 2008;<br>Kraus et al., 2020
HAT3	nonessential in BF cells; acetylates H4K4	ElBashir et al., 2015
ELP3b	negatively regulates rDNA expression	Alsford and Horn, 2011
DOT1A and DOT1B	H3K76 methylation; involved in trypanosome cell cycle regulation and VSG switching	Figueiredo et al., 2008;<br>Janzen et al., 2006b
HDAC1	essential in BF cells; counteracts Sir2rp1 dependent telomeric silencing in BF cells	Ingram and Horn, 2002;<br>Wang et al., 2010
HDAC2	nonessential in BF cells	Ingram and Horn, 2002
HDAC3	essential in BF cells;<br>required for VSG promoter silencing throughout the developmental cycle of *T. brucei*	Ingram and Horn, 2002;<br>Wang et al., 2010
HDAC4	nonessential; knock out results in a cell cycle delay	Ingram and Horn, 2002
Sir2rp1	DNA repair and telomere-adjacent silencing of RNAPI transcription	Alsford et al., 2007

Supplemental Table S3

Supplemental Table S3. Candidate proteins with no specific genomic enrichment by ChIP-seq

Protein name	TriTryp ID	UniProt ID	Protein name	TriTryp ID	UniProt ID
PHD1	Tb927.10.9930	Q389Y3	SET19	Tb927.7.5620	Q582H7
PHD5	Tb927.11.5870	Q385I1	SET20	Tb927.8.2490	Q57XE0
HAT3	Tb927.10.8310	Q38AD3	SET21	Tb927.8.2690	Q57XC0
ELP3a	Tb927.8.5770	Q57X05	SET22	Tb927.8.2710	Q57XB8
NAT2	Tb927.1.4490	Q4GYC9	SET23	Tb927.8.6470	Q57YP8
NAT3	Tb927.10.12830	Q388W3	SET24	Tb927.8.6530	Q57TW0
LPLAT1	Tb927.5.2280	Q582D7	SET25	Tb927.9.1510	Q38FZ5
SET1	Tb11.v5.0422	Q38BE1	SET28	Tb927.9.10100	Q38DZ4
SET3	Tb927.1.4720	Q4GYA6	SET29	Tb927.3.3370	Q57V49
SET4	Tb927.10.11130	Q389M6	DOT1B	Tb927.1.570	Q4GZF2
SET5	Tb927.10.12880	Q388V8	HDAC2	Tb927.11.15600	Q381M6
SET6	Tb927.10.3730	Q38BM6	HDAC4	Tb927.5.2900	D6XGM6
SET7	Tb927.10.4600	-	Sir2rp1	Tb927.7.1690	Q57V41
SET8	Tb927.10.8060	Q38AF8	Sir2rp2	Tb927.8.3140	Q57YZ9
SET9	Tb927.10.9680	Q38A07	Sir2rp3	Tb927.4.2520	Q584D5
SET10	Tb927.11.13560	Q382I1	JMJ1	Tb927.11.1760	Q386X8
SET11	Tb927.11.5120	Q385Q6	JMJ2	Tb927.11.2000	Q386V9
SET13	Tb927.4.2440	Q584E3	LCM1	Tb927.9.12780	Q38DD6
SET14	Tb927.4.3310	Q584A8	CLD1	Tb927.7.660	Q57VR2
SET15	Tb927.5.2770	Q57ZV3	AGO1	Tb927.10.10850	Q389P5
SET16	Tb927.5.3500	Q57UB6	DMT	Tb927.3.1360	Q57XW9
SET17	Tb927.6.3610	Q585V9	NUP110/MLP1	Tb927.11.330	Q387L8
SET18	Tb927.6.910	Q585F5			

Supplemental Table S4

Supplemental Table S4. Significantly enriched interactors detected by proteomic analysis of affinity selected YFP-tagged proteins

CRD1 IP significantly enriched interactors (over untagged control)

UniProt ID	TriTryp ID	Description	$-\log_{10}$ Student's t-test p-value	$\log_2$ (tagged/untagged)
Q4GYF3	Tb927.1.4250	hypothetical protein	5.53089	11.4431
Q38D80	Tb927.9.13470	SET27	5.94395	11.4397
Q57X70	Tb11.v5.0267	CRD1	4.08625	9.18821
Q382F5	Tb927.11.13820	hypothetical protein	6.65416	9.17537
Q582U8	Tb927.3.2350	hypothetical protein	7.10211	9.04339
Q382Z7	Tb927.11.11840	hypothetical protein	6.06473	8.75264
D6XIZ6	Tb927.7.4650	J Biosynthesis Protein 2 (JBP2)	6.33932	7.9307
Q38FV1	Tb927.9.2070	hypothetical protein	2.09326	6.95152
Q38BC1	Tb927.10.4800	hypothetical protein	2.70589	4.47099
Q38BS4	Tb927.10.3220	nucleolar protein	4.25958	4.04605
Q586X9	Tb927.2.3580	transcription elongation factor s-II (TFIIS2-1)	2.30696	3.65927
Q57W25	Tb927.5.800	casein kinase I, isoform 2	5.87744	2.9952
Q387L4	Tb927.11.370	repressor activator protein 1 (RAP1)	3.04907	2.19994
Q580S5	Tb927.3.5500	DNA-directed RNA polymerase II subunit 3 (RPB3)	2.41931	2.06975
Q57YU2	Tb927.8.7400	RNA polymerase IIA largest subunit (RPB1)	2.92416	2.01862

SET27 IP significantly enriched interactors (over untagged control)

UniProt ID	TriTryp ID	Description	$-\log_{10}$ Student's t-test p-value	$\log_2$ (tagged/untagged)
Q4GYF3	Tb927.1.4250	hypothetical protein	5.32451	11.5549
Q38D80	Tb927.9.13470	SET27	4.15023	11.3024
Q57X70	Tb11.v5.0267	CRD1	4.41402	10.8901
Q382F5	Tb927.11.13820	hypothetical protein	4.76568	9.39333
Q382Z7	Tb927.11.11840	hypothetical protein	5.30182	9.15674
D6XIZ6	Tb927.7.4650	J Biosynthesis Protein 2 (JBP2)	5.09272	9.10164
Q582U8	Tb927.3.2350	hypothetical protein	4.70479	8.88102
Q38BT9	Tb927.10.3070	hypothetical protein	3.00745	4.65672
Q57Z72	Tb927.5.3210	small ubiquitin-related modifier	2.2191	4.57882
Q38EP6	Tb927.9.6920	hypothetical protein	2.10149	4.34594
Q38BC1	Tb927.10.4800	hypothetical protein	2.71533	4.25565
Q586X9	Tb927.2.3580	transcription elongation factor s-II (TFIIS2-1)	2.21306	4.05266
Q38BS4	Tb927.10.3220	nucleolar protein	2.90768	4.0165
P86938	Tb927.11.13640	J-binding protein 1 (JBP1)	3.18784	3.5602
Q57W25	Tb927.5.800	casein kinase I, isoform 2	5.83603	3.04821
Q389E6	Tb927.10.11960	hypothetical protein	2.37392	2.98484
Q57W24	Tb927.5.790	casein kinase I isoform 1	3.60939	2.82452
Q57VC7	Tb927.5.1360	nucleoside 2-deoxyribosyltransferase	3.59736	2.56284
Q586H7	Tb927.6.5050	V-type ATPase, C subunit	3.63701	2.53399
Q388E9	Tb927.10.14530	19S proteasome non-atpase subunit 8	2.16045	2.44652
Q57UB5	Tb927.5.3510	structural maintenance of chromosome 3 (SMC3)	3.09014	2.17858
Q38E40	Tb927.9.9550	hypothetical protein	3.18533	2.14501
Q38C86	Tb927.10.1550	proteasome regulatory non-ATP-ase subunit 5	4.10981	2.00096

BDF1 IP significantly enriched interactors (over untagged control)

UniProt ID	TriTryp ID	Description	$-log_{10}$ Student's t-test p-value	log_2 (tagged/untagged)
Q38AE9	Tb927.10.8150	BDF1	4.93793	9.72633
Q57UR8	Tb927.7.4380	BDF4	4.54907	7.93766
Q389A2	Tb927.10.12410	hypothetical protein, conserved	2.98317	3.23467
Q383J7	Tb927.11.10820	Casein kinase II subunit beta	2.73998	2.51666
Q57WL5	Tb927.8.8150	C2 domain containing protein	2.40712	2.18084

BDF4 IP significantly enriched interactors (over untagged control)

UniProt ID	TriTryp ID	Description	$-log_{10}$ Student's t-test p-value	log_2 (tagged/untagged)
Q38AE9	Tb927.10.8150	BDF1	4.188294496	10.46347491
Q57UR8	Tb927.7.4380	BDF4	4.899856567	9.580053965
Q38D21	Tb927.9.14430	Casein kinase II	2.61634521	4.563868841
Q383S2	Tb927.11.10070	BDF3	2.321038534	3.810703278
Q57YY8	Tb927.8.3250	dynein heavy chain	2.187183878	3.700003306
Q57V45	Tb927.3.3410	aspartyl aminopeptidase	2.343303395	3.043106079
Q383J7	Tb927.11.10820	Casein kinase II subunit beta	3.324763121	2.964817047
Q586Q9	Tb927.2.4580	UNC119	3.52554182	2.820368449
Q57VC7	Tb927.5.1360	nucleoside 2-deoxyribosyltransferase	2.708928634	2.726233164
Q38D92	Tb927.9.13320	hypothetical protein	2.267759854	2.656667074
Q382J7	Tb927.11.13400	BDF5	2.3335	2.2561
Q57UC1	Tb927.5.3450	eukaryotic translation initiation factor eIF2A	2.15945	2.01132

BDF3 IP significantly enriched interactors (over untagged control)

UniProt ID	TriTryp ID	Description	$-log_{10}$ Student's t-test p-value	log_2 (tagged/untagged)
Q385P5	Tb927.11.5230	hypothetical protein, conserved	4.60069	15.7219
Q383S2	Tb927.11.10070	BDF3	5.64379	15.3806
Q584F3	Tb927.4.2340	hypothetical protein, conserved	7.46251	14.3231
Q38D92	Tb927.9.13320	hypothetical protein, conserved	4.32584	14.1699
Q382J7	Tb927.11.13400	BDF5	4.65476	13.3481
Q57Y87	Tb927.7.2770	hypothetical protein, conserved	4.90171	11.7022
Q580M8	Tb927.3.4140	hypothetical protein, conserved	4.96735	11.4365
Q585E0	Tb927.6.1070	hypothetical protein, conserved	7.05768	11.3361
Q383C5	Tb927.11.11530	HAT2	4.98438	11.199
Q4FKD8	Tb927.11.18680	dynein light chain LC8	4.38862	7.06557
Q586Q9	Tb927.2.4580	UNC119	3.75556	4.53563
Q57Z72	Tb927.5.3210	small ubiquitin-related modifier	3.50341	4.12536
Q38BR9	Tb927.10.3280	60S ribosomal proteins L38	4.13696	3.68518
Q383E9	Tb927.11.11290	heat shock protein 70	2.54297	3.16894
Q4GYZ8	Tb927.1.2230	small myristoylated protein 1-1	5.00505	2.87762

BDF5 IP significantly enriched interactors (over untagged control)

UniProt ID	TriTryp ID	Description	$-\log_{10}$ Student's t-test p-value	$\log_2$ (tagged/untagged)
Q383S2	Tb927.11.10070	BDF3	5.03137	14.4394
Q385P5	Tb927.11.5230	hypothetical protein, conserved	4.16393	14.2577
Q38D92	Tb927.9.13320	hypothetical protein, conserved	4.03255	13.5657
Q584F3	Tb927.4.2340	hypothetical protein, conserved	4.76342	13.3877
Q382J7	Tb927.11.13400	BDF5	4.64404	13.0329
Q57Y87	Tb927.7.2770	hypothetical protein, conserved	5.38435	11.5875
Q585E0	Tb927.6.1070	hypothetical protein, conserved	5.26236	11.0428
Q580M8	Tb927.3.4140	hypothetical protein, conserved	4.98517	11.001
Q383C5	Tb927.11.11530	HAT2	4.83268	10.0504
Q4FKD8	Tb927.11.18680	dynein light chain LC8	4.21709	6.66445
Q586Q9	Tb927.2.4580	UNC119	3.36202	4.49549
Q38BR9	Tb927.10.3280	60S ribosomal proteins L38	3.73772	4.04956
Q57Z72	Tb927.5.3210	small ubiquitin-related modifier	3.2482	2.9886
Q4GYZ8	Tb927.1.2230	small myristoylated protein 1-1	2.54476	2.80002
Q383E9	Tb927.11.11290	heat shock protein 70	2.77564	2.57324

HAT2 IP significantly enriched interactors (over untagged control)

UniProt ID	TriTryp ID	Description	$-log_{10}$ Student's t-test p-value	log_2 (tagged/untagged)
Q385P5	Tb927.11.5230	hypothetical protein, conserved	6.54066	12.2937
Q38D92	Tb927.9.13320	hypothetical protein, conserved	5.66256	12.1545
Q383S2	Tb927.11.10070	BDF3	5.78366	12.0736
Q383C5	Tb927.11.11530	HAT2	4.7375	11.497
Q382J7	Tb927.11.13400	BDF5	5.32275	11.381
Q584F3	Tb927.4.2340	hypothetical protein, conserved	3.48604	11.3471
Q585E0	Tb927.6.1070	hypothetical protein, conserved	6.59864	10.8439
Q57Y87	Tb927.7.2770	hypothetical protein, conserved	4.81634	10.5086
Q580M8	Tb927.3.4140	hypothetical protein, conserved	5.08953	9.86611
Q4FKD8	Tb927.11.18680	dynein light chain LC8	4.15852	7.369
Q57Z72	Tb927.5.3210	small ubiquitin-related modifier	2.76433	5.35268
Q38BI9	Tb927.10.4110	60S ribosomal protein L30	2.42707	3.23005
Q9N942	Tb927.1.420	retrotransposon hot spot protein 5 (RHS5)	3.10308	3.01725
Q38BE4	Tb927.10.4570	translation elongation factor 2	2.94249	2.17828

BDF2 IP significantly enriched interactors (over untagged control)

UniProt ID	TriTryp ID	Description	$-log_{10}$ Student's t-test p-value	log_2 (tagged/untagged)
Q38AM1	Tb927.10.7420	BDF2	4.94142	13.812
Q586J9	Tb927.2.2190	HDAC3	5.48101	12.925
Q582M3	Tb927.7.6360	Histone H2A.Z	5.5163	8.77685
Q384T0	Tb927.11.7350	Histone H2B.V	5.64211	8.65261
Q38E38	Tb927.9.9580	tubulin tyrosine ligase protein	4.66156	7.7985
Q582V9	Tb927.3.2460	hypothetical protein	4.9989	7.06729
Q383U2	Tb927.11.9870	Telomere-associated protein 1 (TelAP1)	4.1549	6.97577
Q586Z9	Tb927.6.4330	hypothetical protein	3.34214	6.9275
Q388W1	Tb927.10.12850	ttaggg binding factor (TRF)	5.35967	6.55814
Q57XY9	Tb927.3.1560	TRF-Interacting Factor 2 (TIF2)	4.40537	6.3738
Q38FD7	Tb927.9.4000	hypothetical protein	2.89011	5.81918
Q586Q9	Tb927.2.4580	UNC119	4.33193	5.70718
Q57UN4	Tb927.7.4040	hypothetical protein	3.53327	4.9661
Q385L3	Tb927.11.5550	DNA polymerase theta (POLQ)	3.27198	4.95997
Q57ZL0	Tb927.3.3020	actin-like protein	5.7851	4.94816
Q584P9	Tb927.6.2570	SUMO-interacting motif-containing protein	4.62419	4.90393
Q383K6	Tb927.11.10730	SWI/SNF-related helicase	3.65983	4.76426
Q389H2	Tb927.10.11690	YEATS family	3.27706	4.27104
Q38EB9	Tb927.9.8520	hypothetical protein	2.92994	4.2494
Q57YH0	Tb927.8.600	Bucentaur or craniofacial development	2.94405	3.88251
Q389D3	Tb927.10.12100	RNA-binding protein 7B	2.71219	3.49313
Q385T8	Tb927.11.4800	clathrin coat assembly protein	3.80316	3.47689
Q38BZ3	Tb927.10.2520	PrimPol-like protein 2	2.73316	3.47355
Q583J3	Tb927.4.2000	ruvB-like DNA helicase	3.61615	3.41918
Q580C6	Tb927.4.980	actin	3.02507	3.32672
Q38C43	Tb927.10.2000	actin-like protein	2.37492	3.31999
Q57WS6	Tb927.7.1240	Sphingosine kinase	2.11279	3.29644
Q581V4	Tb927.4.1270	ruvB-like DNA helicase	3.29543	3.20032
Q385E0	Tb927.11.6290	HIT zinc finger	2.49815	3.0698

BDF2 IP significantly enriched interactors (over untagged control) - continued

UniProt ID	TriTryp ID	Description	$-\log_{10}$ Student's t-test p-value	$\log_2$ (tagged/untagged)
Q57YW0	Tb927.8.3530	glycerol-3-phosphate dehydrogenase [NAD+], glycosomal	5.18765	2.94186
Q389G9	Tb927.10.11720	ZCW1	2.89967	2.89912
Q57ZS8	Tb927.5.2090	kinesin	3.26358	2.74008
Q57W25	Tb927.5.800	casein kinase I, isoform 2	3.7468	2.69126
Q389D9	Tb927.10.12030	hypothetical protein	3.67541	2.67071
Q384I8	Tb927.11.8310	class I transcription factor A, subunit 4 (CITFA-4)	2.451	2.64382
Q385A8	Tb927.11.6610	hypothetical protein	2.16774	2.44445
Q387B3	Tb927.11.1390	class I transcription factor A, subunit 1 (CITFA-1)	3.77834	2.28532
Q38DS7	Tb927.9.10960	ATP-dependent DEAD/H RNA helicase	4.30734	2.26913
Q57XM9	Tb927.7.2450	SUMO-interacting motif-containing protein	2.87997	2.2086
Q38D76	Tb927.9.13510	Poly(A)-specific ribonuclease PARN-3	2.66067	2.00854

HDAC3 IP significantly enriched interactors (over untagged control)

UniProt ID	TriTryp ID	Description	$-log_{10}$ Student's t-test p-value	log_2 (tagged/untagged)
Q586J9	Tb927.2.2190	HDAC3	5.46467	12.9502
Q57XY9	Tb927.3.1560	TRF-Interacting Factor 2 (TIF2)	5.1545	8.95426
Q586Z9	Tb927.6.4330	hypothetical protein	4.54883	8.81523
Q38AM1	Tb927.10.7420	BDF2	4.99548	8.60155
Q388W1	Tb927.10.12850	ttaggg binding factor (TRF)	4.01868	8.55698
Q383U2	Tb927.11.9870	Telomere-associated protein 1 (TelAP1)	3.9975	8.24039
Q38EB9	Tb927.9.8520	hypothetical protein	3.65687	7.71109
Q385L3	Tb927.11.5550	DNA polymerase theta (POLQ)	4.17948	7.53688
Q582M3	Tb927.7.6360	Histone H2A.Z	4.33192	7.20786
Q582V9	Tb927.3.2460	hypothetical protein	3.9418	7.12454
Q38FD7	Tb927.9.4000	hypothetical protein	3.40403	7.00728
Q38BZ3	Tb927.10.2520	PrimPol-like protein 2	4.02346	6.39412
Q384T0	Tb927.11.7350	Histone H2B.V	4.86712	6.21943
Q387L4	Tb927.11.370	repressor activator protein 1 (RAP1)	3.94259	5.0573
Q586Q9	Tb927.2.4580	UNC119	3.48225	4.21712
Q57UB5	Tb927.5.3510	structural maintenance of chromosome 3 (SMC3)	3.94742	4.09873
Q57W25	Tb927.5.800	casein kinase I, isoform 2	4.09069	4.08785
Q580Q4	Tb927.4.5310	Repressor of differentiation kinase 2	3.04617	3.66189
Q388S9	Tb927.10.13180	Nrap protein	2.48075	3.65437
Q4GZ82	Tb927.1.1370	rRNA biogenesis protein	2.54895	3.52187
Q38D76	Tb927.9.13510	Poly(A)-specific ribonuclease PARN-3	3.07138	3.34021
Q580T1	Tb927.3.5440	SNF2 DNA repair protein	2.29208	3.18214
Q38DK9	Tb927.9.11850	structural maintenance of chromosome 1 (SMC1)	2.34857	2.96831
Q385E2	Tb927.11.6270	inositol-1,4,5-trisphosphate (IP3) 5-phosphatase	2.02254	2.94661
Q57WS6	Tb927.7.1240	Sphingosine kinase	3.01619	2.87586
Q385P5	Tb927.11.5230	hypothetical protein	3.09052	2.2089
Q38DH5	Tb927.9.12300	replication factor C, subunit 3	2.75055	2.00741

SET26 IP significantly enriched interactors (over untagged control)

UniProt ID	TriTryp ID	Description	$-log_{10}$ Student's t-test p-value	log_2 (tagged/untagged)
Q389G9	Tb927.10.11720	ZCW1	7.02945	12.2049
Q38AF4	Tb927.10.8100	SET26	4.22372	9.24843
Q384T0	Tb927.11.7350	Histone H2B.V	2.89069	8.17785
Q582M3	Tb927.7.6360	Histone H2A.Z	4.46897	6.16313
Q580R3	Tb927.3.5620	FACT complex subunit SPT16	3.63648	5.89062
Q385D4	Tb927.11.6350	AAA ATPase (BDF7)	3.91991	5.78368
Q388G3	Tb927.10.14390	FACT complex subunit POB3	3.37037	5.22017
Q387X7	Tb927.10.15350	Histone H3.V	3.6525	5.00264
Q57Z31	Tb927.5.4170	Histone H4	3.36077	4.61553
Q383E9	Tb927.11.11290	heat shock protein 70	5.03661	4.61157
Q384B5	Tb927.11.9130	Replication factor A protein 1	3.37426	4.60043
Q57YA3	Tb927.7.2820	Histone H2A	4.0639	4.55846
Q386Y4	Tb927.11.1700	hypothetical protein	2.88207	4.39468
Q583H5	Tb927.4.3810	DNA-directed RNA polymerase II subunit 2 (RPB2)	2.33135	4.08113
Q386P3	Tb927.11.2670	Nucleoporin NUP59	3.46948	4.04731
Q38D92	Tb927.9.13320	hypothetical protein	2.47669	3.66993
Q389T1	Tb927.10.10590	Histone H2B	4.31282	3.59015
Q583S0	Tb927.6.2640	importin alpha subunit	2.30335	3.56287
Q38AE0	Tb927.10.8240	hypothetical protein	2.78086	3.54509
Q580N2	Tb927.3.4100	Pyruvate transporter	4.27969	3.47669
Q38A77	Tb927.10.8970	kinetoplast-associated protein 4 isoform 2	3.85018	3.44607
Q38E19	Tb927.9.9810	hypothetical protein	3.20778	3.29031
Q57YU2	Tb927.8.7400	RNA polymerase IIA largest subunit (RPB1)	2.15392	3.152
Q385C3	Tb927.11.6460	hypothetical protein	3.31061	3.00282
Q38FW0	Tb927.9.1980	hypothetical protein	3.74352	3.00098
Q38D21	Tb927.9.14430	Casein kinase II	4.09447	2.94132
Q57WL5	Tb927.8.8150	C2 domain containing protein	4.11178	2.93721
Q4GYX7	Tb927.1.2430	Histone H3	2.39318	2.84673
Q57ZS7	Tb927.5.2080	GMP reductase	2.82782	2.811

SET26 IP significantly enriched interactors (over untagged control) - continued

UniProt ID	TriTryp ID	Description	$-\log_{10}$ Student's t-test p-value	$\log_2$ (tagged/untagged)
Q387Q3	Tb927.10.16120	inosine-5'-monophosphate dehydrogenase	3.08141	2.80256
Q388C7	Tb927.10.14770	Associated kinase of Tb14-3-3	2.07295	2.61634
Q388K3	Tb927.10.13960	paralyzed flagella protein 20	2.60087	2.52675
Q389A2	Tb927.10.12410	hypothetical protein	3.01927	2.48542
Q38DT1	Tb927.9.10920	kinetoplastid kinetochore protein 3 (KKT3)	2.78001	2.45683
Q584R4	Tb927.6.2420	p22 protein precursor	3.74551	2.4026
Q386D5	Tb927.11.3770	Dpy-30 motif containing protein	2.77261	2.29603
Q381T5	Tb927.11.15000	survival of motor neuron (SMN)-like protein	2.214	2.23543
Q38B98	Tb927.10.5030	ubiquitin/ribosomal protein S27a	3.55043	2.16014
Q580S5	Tb927.3.5500	DNA-directed RNA polymerase II subunit 3 (RPB3)	3.71295	2.12249
Q582W2	Tb927.3.2490	hypothetical protein	2.03244	2.09296
Q57X47	Tb927.5.4570	Flagellum adhesion protein 3	2.25438	2.09208
Q57YE9	Tb927.8.820	VID27 cytoplasmic protein	2.57063	2.0567

ZCW1 IP significantly enriched interactors (over untagged control)

UniProt ID	TriTryp ID	Description	$-log_{10}$ Student's t-test p-value	log_2 (tagged/untagged)
Q57UN4	Tb927.7.4040	hypothetical protein	5.00494	11.7469
Q383K6	Tb927.11.10730	SWI/SNF-related helicase	6.66968	11.6905
Q580C6	Tb927.4.980	actin	6.24716	11.6278
Q389H2	Tb927.10.11690	YEATS family	4.11583	11.561
Q57ZL0	Tb927.3.3020	actin-like protein	4.47839	11.3822
Q584P9	Tb927.6.2570	SUMO-interacting motif-containing protein	3.71735	10.8057
Q38C43	Tb927.10.2000	actin-like protein	4.04326	10.1429
Q385E0	Tb927.11.6290	HIT zinc finger	5.72496	10.0378
Q389G9	Tb927.10.11720	ZCW1	5.52659	9.89842
Q57YH0	Tb927.8.600	Bucentaur or craniofacial development	4.74135	9.8613
Q583J3	Tb927.4.2000	ruvB-like DNA helicase	5.70316	9.40665
Q38AF4	Tb927.10.8100	SET26	4.38096	8.99975
Q385I5	Tb927.11.5830	YL1 nuclear protein	4.51071	8.95025
Q581V4	Tb927.4.1270	ruvB-like DNA helicase	6.30647	8.89021
Q385D4	Tb927.11.6350	AAA ATPase (BDF7)	3.83483	6.00911
Q384T0	Tb927.11.7350	Histone H2B.V	4.47655	5.79935
Q582M3	Tb927.7.6360	Histone H2A.Z	5.32221	5.55756
Q388G3	Tb927.10.14390	FACT complex subunit POB3	4.00616	5.16272
Q387D1	Tb927.11.1210	Domain of unknown function (DUF4470)	4.0592	4.56921
Q583S0	Tb927.6.2640	importin alpha subunit	2.95249	4.54393
Q383E9	Tb927.11.11290	heat shock protein 70	5.58338	4.41933
Q386P3	Tb927.11.2670	Nucleoporin NUP59	2.14711	3.80024
Q580R3	Tb927.3.5620	FACT complex subunit SPT16	3.61713	3.74787
Q38AM1	Tb927.10.7420	BDF2	2.9484	3.65793
Q38D21	Tb927.9.14430	Casein kinase II	2.69264	3.60069
Q385P5	Tb927.11.5230	hypothetical protein protein	3.28779	3.24957
Q57XG4	Tb927.8.2250	Fungal tRNA ligase phosphodiesterase domain	2.20582	3.13698
Q386Y7	Tb927.11.1670	cysteine desulfurase	3.33443	3.08449
Q57Z31	Tb927.5.4170	Histone H4	3.9249	3.01404

ZCW1 IP significantly enriched interactors (over untagged control) - continued

UniProt ID	TriTryp ID	Description	$-log_{10}$ Student's t-test p-value	log_2 (tagged/untagged)
Q57YA3	Tb927.7.2820	Histone H2A	3.46827	2.92822
Q386Y4	Tb927.11.1700	hypothetical protein	3.49597	2.77325
Q4GYX7	Tb927.1.2430	Histone H3	4.12096	2.61517
Q4GYK7	Tb927.1.3670	expression site-associated gene 8 (ESAG8) protein	2.35331	2.57668
Q389T1	Tb927.10.10590	Histone H2B	3.33103	2.55487
Q586Q9	Tb927.2.4580	UNC119	2.50076	2.23221
Q382J7	Tb927.11.13400	BDF5	2.29002	2.11787
Q387T9	Tb927.10.15750	Tripartite attachment complex protein 197	3.57696	2.07161
Q57ZX9	Tb927.5.3030	Intraflagellar transport protein 121	2.63744	2.74248

BDF6 IP significantly enriched interactors (over untagged control)

UniProt ID	TriTryp ID	Description	$-log_{10}$ Student's t-test p-value	log_2 (tagged/untagged)
Q57ZF8	Tb927.7.5310	YEATS family	6.11969	12.5739
Q4GYN3	Tb927.1.3400	BDF6	5.6584	12.1121
Q388I3	Tb927.10.14190	hypothetical protein, conserved	5.54719	11.8692
Q4GZG2	Tb927.1.650	conserved protein, unknown function	5.39763	11.6205
Q57UK4	Tb927.8.5320	hypothetical protein, conserved	4.60683	11.1868
D6XIY7	Tb927.7.4560	HAT1	5.87644	10.7995
Q38FN2	Tb927.9.2910	EAF6 (NuA4 complex component)	3.68363	9.94123
Q386G8	Tb927.11.3430	hypothetical protein, conserved	4.58029	8.80534
Q585C3	Tb927.6.1240	hypothetical protein, conserved	5.19258	7.78062
Q57UX1	Tb927.8.4810	prohibitin 1	2.86836	2.39806
Q57Z72	Tb927.5.3210	small ubiquitin-related modifier	3.24902	2.05029

HAT1 IP significantly enriched interactors (over untagged control)

UniProt ID	TriTryp ID	Description	$-\log_{10}$ Student's t-test p-value	$\log_2$ (tagged/untagged)
Q57ZF8	Tb927.7.5310	YEATS family	5.21336	12.6562
Q388I3	Tb927.10.14190	hypothetical protein, conserved	4.12823	12.0313
Q57UK4	Tb927.8.5320	hypothetical protein, conserved	5.21298	11.3855
Q4GZG2	Tb927.1.650	conserved protein, unknown function	4.49989	11.2084
Q4GYN3	Tb927.1.3400	BDF6	5.49118	11.1912
Q386G8	Tb927.11.3430	hypothetical protein, conserved	6.13574	10.9019
D6XIY7	Tb927.7.4560	HAT1	4.97472	10.4031
Q38FN2	Tb927.9.2910	EAF6 (NuA4 complex component)	4.74772	10.1789
Q585C3	Tb927.6.1240	hypothetical protein, conserved	5.64965	7.83824
Q383W5	Tb927.11.9640	glycyl-tRNA synthetase	3.96282	2.34455
Q586Q9	Tb927.2.4580	UNC119	2.93824	2.33758
Q389D8	Tb927.10.12040	mitogen-activated protein kinase 11	3.69224	2.23945

EAF6 IP significantly enriched interactors (over untagged control)

UniProt ID	TriTryp ID	Description	$-log_{10}$ Student's t-test p-value	log_2 (tagged/untagged)
Q388I3	Tb927.10.14190	hypothetical protein, conserved	5.6127	12.6188
Q57ZF8	Tb927.7.5310	YEATS family	5.31752	12.5041
D6XIY7	Tb927.7.4560	HAT1	4.77593	11.562
Q4GYN3	Tb927.1.3400	BDF6	4.64662	11.43
Q4GZG2	Tb927.1.650	conserved protein, unknown function	5.23768	10.8304
Q386G8	Tb927.11.3430	hypothetical protein, conserved	4.27064	10.5847
Q57UK4	Tb927.8.5320	hypothetical protein, conserved	4.60791	10.5651
Q585C3	Tb927.6.1240	hypothetical protein, conserved	3.91776	9.07108
Q389Y3	Tb927.10.9930	PHD1	4.89452	8.76258
Q38FN2	Tb927.9.2910	EAF6 (NuA4 complex component)	3.44309	8.58075
Q384N0	Tb927.11.7880	hypothetical protein, conserved	3.45527	6.17389
Q38AD3	Tb927.10.8310	HAT3	2.86916	5.27809
Q38C15	Tb927.10.2290	chaperone protein DnaJ	4.44281	5.13168
Q586Q9	Tb927.2.4580	UNC119	2.53018	4.21007
Q57V04	Tb927.7.2070	heat shock protein DNAJ	3.00472	4.10741
Q38DC5	Tb927.9.12900	RNA polymerase-associated protein LEO1	2.72506	2.77654
Q586X9	Tb927.2.3580	transcription elongation factor s-II (TFIIS2-1)	2.50704	2.56127
D6XHJ3	Tb927.6.1090	proteasome regulatory ATPase subunit 3	2.48845	2.47009
Q383D0	Tb927.11.11480	Trichohyalin	2.66667	2.22829
Q57Y42	Tb927.8.7980	Pyrophosphate-energized vacuolar membrane proton pump 2	2.4109	-2.87184

HDAC1 IP significantly enriched interactors (over untagged control)

UniProt ID	TriTryp ID	Description	$-\log_{10}$ Student's t-test p-value	$\log_2$ (tagged/untagged)
Q38FV1	Tb927.9.2070	hypothetical protein	6.05488	11.7449
Q57TZ5	Tb927.7.1650	hypothetical protein	5.39599	11.4167
Q584W0	Tb927.6.3170	hypothetical protein	4.1002	10.7475
Q57WX2	Tb927.3.890	hypothetical protein	4.84634	9.67638
Q38C74	Tb927.10.1680	HDAC1	4.00622	7.22132
Q585H7	Tb927.4.3730	hypothetical protein	4.26412	6.34073

BDF7 IP significantly enriched interactors (over untagged control)

UniProt ID	TriTryp ID	Description	$-\log_{10}$ Student's t-test p-value	$\log_2$ (tagged/untagged)
Q385D4	Tb927.11.6350	BDF7	7.67914	14.416
Q582P7	Tb927.3.4880	nucleosome assembly protein (NAP2)	5.41527	10.1791
Q4GZ00	Tb927.1.2210	nucleosome assembly protein (NAP1)	5.53343	8.66135
Q38D21	Tb927.9.14430	Casein kinase II	4.07334	7.92549
Q383J7	Tb927.11.10820	Casein kinase II subunit beta	5.39243	7.86722
Q386Y7	Tb927.11.1670	cysteine desulfurase	5.11041	6.62681
Q585P0	Tb927.6.1710	hypothetical protein, conserved	2.99608	6.03265
Q586Q9	Tb927.2.4580	UNC119	2.88511	4.59575
Q385C7	Tb927.11.6420	hypothetical protein, conserved	5.21268	4.53223
Q388S2	Tb927.10.13250	hypothetical protein, conserved	3.00103	4.37745
Q388P7	Tb927.10.13510	zinc metallopeptidase	4.53851	4.27171
Q381W2	Tb927.11.14730	Metalloprotease M41 FtsH	2.77481	4.13688
Q389E2	Tb927.10.12000	iron-sulfur cluster assembly protein	4.06848	3.71724
Q38DH1	Tb927.9.12340	hypothetical protein, conserved	5.25919	3.69929
Q387Z3	Tb927.10.15180	nucleosome assembly protein (NAP3)	4.13692	3.43985
Q383E9	Tb927.11.11290	heat shock protein 70	2.87951	3.26538
Q38BN5	Tb927.10.3640	nuclear transmembrane protein	3.5369	3.14475
Q57WG0	Tb927.7.3450	protein I/6	2.31469	3.07365
Q38BJ3	Tb927.10.4070	hypothetical protein, conserved	2.77233	3.07108
Q386R6	Tb927.11.2440	Tubulin cofactor C domain-containing protein 1	5.83909	3.04755
Q57YB8	Tb927.7.3080	Kinetochore interacting protein 4 (KKIP4)	4.07542	3.00762
Q584Z0	Tb927.6.3500	endosomal trafficking protein RME-8	2.8357	2.94232
B3GVR6	Tb427.BES126.2	Expression site-associated gene 7 (ESAG7) protein	3.175	2.75723
Q584K3	Tb927.4.5020	DNA-directed RNA polymerase II subunit RPB1-A	3.43896	2.68048
Q585L0	Tb927.4.3400	hypothetical protein, conserved	3.55021	2.30728
B3GVQ1	Tb427.BES56.7	Expression site-associated gene 8 (ESAG8) protein	3.32151	2.22846
Q38D92	Tb927.9.13320	hypothetical protein, conserved	2.8659	2.16731
Q384U3	Tb927.11.7220	neurobeachin/beige protein	2.1597	2.06637
Q580Q4	Tb927.4.5310	Repressor of differentiation kinase 2	3.01007	2.05326

TFIIS2-2 IP significantly enriched interactors (over untagged control)

UniProt ID	TriTryp ID	Description	$-log_{10}$ Student's t-test p-value	log_2 (tagged/untagged)
Q582N0	Tb927.3.5070	hypothetical protein	4.99559	14.1957
Q38DC5	Tb927.9.12900	RNA polymerase-associated protein LEO1	8.53225	14.0585
Q57V64	Tb927.3.3220	RNA polymerase-associated protein CTR9	5.09399	13.7842
Q586X9	Tb927.2.3580	transcription elongation factor s-II (TFIIS2-1)	4.71915	13.7123
Q57UN3	Tb927.7.4030	hypothetical protein	4.37899	12.2545
Q383Q6	Tb927.11.10230	RNA polymerase-associated protein CDC73	4.83204	11.654
Q586Y0	Tb927.2.3480	transcription elongation factor s-II (TFIIS2-2)	4.58521	11.4279
Q586C8	Tb927.2.5810	Holliday-junction resolvase-like of SPT6/SH2 domain	3.46556	6.32434
Q583H5	Tb927.4.3810	DNA-directed RNA polymerase II subunit 2 (RPB2)	4.72879	5.87253
Q57YU2	Tb927.8.7400	RNA polymerase IIA largest subunit (RPB1)	2.56766	5.65476
Q383E9	Tb927.11.11290	heat shock protein 70	3.89194	5.60172
Q57Z69	Tb927.5.3240	hypothetical protein	3.53278	5.28492
Q38C15	Tb927.10.2290	chaperone protein DnaJ	3.90992	5.05717
Q38BC1	Tb927.10.4800	hypothetical protein	4.30469	4.65933
Q580N2	Tb927.3.4100	Pyruvate transporter	3.01628	4.3271
Q38DK9	Tb927.9.11850	structural maintenance of chromosome 1 (SMC1)	4.50362	4.13835
Q4GYZ8	Tb927.1.2230	small myristoylated protein 1-1	2.92265	4.08843
Q38DH5	Tb927.9.12300	replication factor C, subunit 3	3.86106	3.9829
Q38AE0	Tb927.10.8240	hypothetical protein	4.76951	3.95355
Q57X47	Tb927.5.4570	Flagellum adhesion protein 3	3.91342	3.95229
Q389A2	Tb927.10.12410	hypothetical protein	3.78775	3.88992
Q57V04	Tb927.7.2070	heat shock protein DNAJ	3.60006	3.81925
Q9N942	Tb927.1.420	retrotransposon hot spot protein 5 (RHS5)	2.72687	3.73281
Q580S5	Tb927.3.5500	DNA-directed RNA polymerase II subunit 3	4.01615	3.69604
Q383S2	Tb927.11.10070	BDF3	2.12292	3.67039
Q389E6	Tb927.10.11960	hypothetical protein	4.15932	3.66012
Q582N5	Tb927.3.5020	Flagellar Member 6	2.3036	3.64086
Q381Q0	Tb927.11.15350	RNA-binding protein	4.57513	3.61854
Q383X4	Tb927.11.9550	replication factor C, subunit 4	3.8174	3.54192

TFIIS2-2 IP significantly enriched interactors (over untagged control) - continued

UniProt ID	TriTryp ID	Description	$-log_{10}$ Student's t-test p-value	log_2 (tagged/untagged)
Q57YB8	Tb927.7.3080	Kinetochore interacting protein 4	4.60916	3.45554
Q382X6	Tb927.11.12050	hypothetical protein	3.11596	3.4261
Q385C3	Tb927.11.6460	hypothetical protein	3.11483	3.40504
Q388C7	Tb927.10.14770	Associated kinase of Tb14-3-3	3.33222	3.40478
Q385K3	Tb927.11.5650	replication factor C, subunit 1	3.48977	3.38828
Q38D92	Tb927.9.13320	hypothetical protein	4.34031	3.1995
Q381Y8	Tb927.11.14490	RNA polymerase subunit (RPB7)	2.88983	3.1697
Q585Y7	Tb927.6.3890	replication factor C, subunit 2	3.38762	3.12531
Q386Y8	Tb927.11.1660	vesicular transport protein (CDC48 homologue)	3.59205	3.10783
Q38AG5	Tb927.10.7990	replication factor C subunit 3	2.73335	3.06639
Q581W7	Tb927.8.2150	hypothetical protein	2.0861	2.96903
Q387X4	Tb927.10.15390	Flagellum attachment zone protein 7	2.13513	2.93651
Q38D56	Tb927.9.13970	hypothetical protein	2.66294	2.87394
Q381J5	Tb927.11.15910	iron superoxide dismutase	2.7698	2.85969
Q582W2	Tb927.3.2490	hypothetical protein	2.22011	2.83506
Q381B2	Tb927.11.16740	chaperone protein DnaJ	3.3618	2.75458
Q57WL5	Tb927.8.8150	C2 domain containing protein	3.36274	2.73377
Q580R3	Tb927.3.5620	FACT complex subunit SPT16	2.30752	2.71646
Q38D21	Tb927.9.14430	Casein kinase II	3.7948	2.66072
Q388V2	Tb927.10.12940	predicted zinc finger protein	2.18054	2.66
Q386Q6	Tb927.11.2540	hypothetical protein	2.50232	2.54926
Q388R5	Tb927.10.13320	DNA-directed RNA polymerase II/III subunit	2.82681	2.48833
Q38DM0	Tb927.9.11740	cyclophilin type peptidyl-prolyl cis-trans isomerase	4.05265	2.44251
Q388X5	Tb927.10.12680	60S ribosomal protein L34	3.33235	2.40742
Q38EX8	Tb927.9.5770	Tryparedoxin peroxidase	3.94584	2.40469
Q583Q4	Tb927.6.2800	hypothetical protein	4.36211	2.31914
Q386D5	Tb927.11.3770	Dpy-30 motif containing protein	2.61587	2.28773
Q387U0	Tb927.10.15740	hypothetical protein	3.34011	2.27737
Q38DT2	Tb927.9.10890	Regulatory subunit of type II PKA R-subunit	2.22984	2.2477

TFIIS2-2 IP significantly enriched interactors (over untagged control) - continued

UniProt ID	TriTryp ID	Description	$-log_{10}$ Student's t-test p-value	log_2 (tagged/untagged)
Q583S0	Tb927.6.2640	importin alpha subunit	2.42317	2.22998
Q38E79	Tb927.9.9100	hypothetical protein	3.45709	2.22126
Q381F0	Tb927.11.16360	hypothetical protein	2.14231	2.15977

DOT1A IP significantly enriched interactors (over untagged control)

UniProt ID	TriTryp ID	Description	$-log_{10}$ Student's t-test p-value	log_2 (tagged/untagged)
Q4GYF6	Tb927.1.4220	Ydr279p protein family (RNase H2 complex component)	5.13483	10.0395
Q581Z0	Tb927.8.1920	DOT1A	4.76344	9.91715
Q38B94	Tb927.10.5070	ribonuclease H	6.02426	9.22535
Q38F34	Tb927.9.5190	proliferating cell nuclear antigen (PCNA)	5.74715	9.04071
Q4GYA5	Tb927.1.4730	Ribonuclease H2 non-catalytic subunit (Ylr154p-like)	5.55902	7.96804
Q583S0	Tb927.6.2640	importin alpha subunit	5.11794	4.35021
Q586Q9	Tb927.2.4580	UNC119	3.18195	3.15221
Q387Q3	Tb927.10.16120	IMPDH1	3.77913	2.54422
Q383E9	Tb927.11.11290	HSP70	3.62208	2.51085

TRF IP significantly enriched interactors (over untagged control)

UniProt ID	TriTryp ID	Description	$-log_{10}$ Student's t-test p-value	log_2 (tagged/untagged)
Q388W1	Tb927.10.12850	ttaggg binding factor (TRF)	4.5821	10.2585
Q586Z9	Tb927.6.4330	telomere-associated protein	5.17342	9.60136
Q38FD7	Tb927.9.4000	hypothetical protein, conserved	7.10736	9.53791
Q57XY9	Tb927.3.1560	TRF-Interacting Factor 2 (TIF2)	5.76737	9.27732
Q383U2	Tb927.11.9870	Telomere-associated protein 1 (TelAP1)	5.50112	8.97668
Q385L3	Tb927.11.5550	DNA polymerase theta (POLQ)	5.76751	8.19689
Q38BZ3	Tb927.10.2520	PrimPol-like protein 2	5.07689	7.84982
Q387L4	Tb927.11.370	repressor activator protein 1 (RAP1)	3.75433	6.0696
Q586J9	Tb927.2.2190	histone deacetylase 3 (HDAC3)	3.22852	4.91487
Q387X7	Tb927.10.15350	Histone H3.V	2.85869	3.715
Q385E2	Tb927.11.6270	inositol-1,4,5-trisphosphate (IP3) 5-phosphatase	4.2341	3.60051
Q38FR6	Tb927.9.2520	microtubule-associated protein (GB4)	2.41828	3.5649
Q57YA3	Tb927.7.2820	Histone H2A	2.40159	3.11452
Q586Q9	Tb927.2.4580	UNC119	2.78003	2.81699
Q583S0	Tb927.6.2640	importin alpha subunit	2.59694	2.64684
Q57VM1	Tb927.8.1330	60S ribosomal protein L7a	3.4478	2.59139
Q388B1	Tb927.10.14950	Zinc finger CCCH domain-containing protein 40 (ZC3H40)	3.0887	2.5017
Q384T0	Tb927.11.7350	Histone H2B.V	2.51354	2.43428
Q38CW9	Tb927.9.15420	60S ribosomal protein L32	2.98648	2.14164
Q38AC1	Tb927.10.8430	40S ribosomal protein S12	2.40777	2.05111
Q38EY6	Tb927.9.5690	60S acidic ribosomal protein	2.4053	2.03389

TBP IP significantly enriched interactors (over untagged control)

UniProt ID	TriTryp ID	Description	$-log_{10}$ Student's t-test p-value	log_2 (tagged/untagged)
Q38E05	Tb927.9.9970	Small nuclear RNA gene activation protein 50 (SNAP50)	5.10704	10.3893
Q599N4	Tb927.10.15570	Transcription factor IIA alpha-beta subunit (TFIIA-1)	5.57909	10.3761
Q599N5	Tb927.10.7070	Small nuclear RNA gene activation protein 3 (SNAP3)	5.25598	10.1384
Q387R9	Tb927.10.15950	TATA-box-binding protein (TBP)	5.05796	9.56953
Q599N6	Tb927.5.3910	Small nuclear RNA activating protein 2 (SNAP2)	5.26631	9.28801
Q387K4	Tb927.11.470	TFIIB-related factor BRF1	6.2795	9.00377
Q599N3	Tb927.10.4840	Transcription factor IIA gamma subunit	5.52352	8.6298
Q387V6	Tb927.10.15570	transcription factor IIA (TFIIA-1)	5.33766	7.33029
Q38DC3	Tb927.9.12950	hypothetical protein, conserved	4.03431	7.05365
Q384L9	Tb927.11.8000	hypothetical protein, conserved	3.84548	6.45067

ELP3b IP significantly enriched interactors (over untagged control)

UniProt ID	TriTryp ID	Description	$-\log_{10}$ Student's t-test p-value	$\log_2$ (tagged/untagged)
Q57YY2	Tb927.8.3310	Elongator-like Protein 3b (ELP3b)	4.31259	12.0095
Q585H2	Tb927.2.1210	retrotransposon hot spot protein 4 (RHS4)	2.23588	2.67507
Q586Q9	Tb927.2.4580	UNC119	2.91717	2.56359
Q57ZH2	Tb927.7.5170	60S ribosomal protein L25	2.66388	2.39845
Q583F5	Tb927.4.4700	hypothetical protein, conserved	2.01684	2.24062
Q387A9	Tb927.11.1430	Component of motile flagella 2	2.18741	2.04843

PHD2 IP significantly enriched interactors (over untagged control)

UniProt ID	TriTryp ID	Description	$-\log_{10}$ Student's t-test p-value	$\log_2$ (tagged/untagged)
Q387D3	Tb11.v5.0388	Ring finger domain/PHD-finger (PHD2)	3.9765	7.4299

PHD4 IP significantly enriched interactors (over untagged control)

UniProt ID	TriTryp ID	Description	$-\log_{10}$ Student's t-test p-value	$\log_2$ (tagged/untagged)
Q57Z97	Tb927.3.2140	transcription activator (PHD4)	5.39723	9.03261
Q587E7	Tb927.2.3370	UDP-Gal or UDP-GlcNAc-dependent glycosyltransferase	2.30539	2.0727

PHD1 IP significantly enriched interactors (over untagged control)

UniProt ID	TriTryp ID	Description	$-log_{10}$ Student's t-test p-value	log_2 (tagged/untagged)
Q389Y3	Tb927.10.9930	PHD1	5.75346	10.6464
Q384N0	Tb927.11.7880	hypothetical protein, conserved	4.14919	10.3396
Q38FN2	Tb927.9.2910	EAF6 (NuA4 complex component)	4.78537	8.70341
Q38AD3	Tb927.10.8310	HAT3	5.36842	7.71911

PHD5 IP significantly enriched interactors (over untagged control)

UniProt ID	TriTryp ID	Description	$-log_{10}$ Student's t-test p-value	log_2 (tagged/untagged)
Q385I1	Tb927.11.5870	PHD5	5.44582	11.5317
Q586Q9	Tb927.2.4580	UNC119	3.37754	3.63062
Q386H2	Tb927.11.3390	Concanavalin A-like lectin/glucanases superfamily/SPRY domain/HECT-domain (ubiquitin-transferase)	2.09534	2.68469

HAT3 IP significantly enriched interactors (over untagged control)

UniProt ID	TriTryp ID	Description	$-log_{10}$ Student's t-test p-value	log_2 (tagged/untagged)
Q389Y3	Tb927.10.9930	PHD1	4.63916	7.69124
Q384N0	Tb927.11.7880	hypothetical protein, conserved	4.3526	6.73332
Q38FN2	Tb927.9.2910	EAF6 (NuA4 complex component)	3.41918	5.63806
Q38AD3	Tb927.10.8310	HAT3	3.25866	5.62241
Q57YG1	Tb927.8.690	peptidyl-prolyl cis-trans isomerase/rotamase, putative	2.94219	2.48432
B3GVR6	Tb427.BES126.2	Expression site-associated gene 7 (ESAG7) protein	2.29797	2.02109

AGO1 IP significantly enriched interactors (over untagged control)

UniProt ID	TriTryp ID	Description	$-\log_{10}$ Student's t-test p-value	$\log_2$ (tagged/untagged)
Q389P5	Tb927.10.10850	AGO1	5.65625	13.9389
Q38A86	Tb927.10.8880	RNA interference factor 4 (RIF4)	4.02946	9.3504
Q586Q9	Tb927.2.4580	UNC119	2.68214	5.73447
Q389B0	Tb927.10.12330	zinc finger protein family member, putative (ZC3H34)	3.65955	3.58715
Q38E63	Tb927.9.9290	polyadenylate-binding protein 1 (PABP1)	2.42994	2.75077
Q585M4	Tb927.6.1870	Eukaryotic translation initiation factor 4E-4 (EIF4E4)	3.06851	2.25288

NUP110 IP significantly enriched interactors (over untagged control)

UniProt ID	TriTryp ID	Description	$-log_{10}$ Student's t-test p-value	log_2 (tagged/untagged)
Q387L8	Tb927.11.330	Myosin-like protein 1 (NUP110/MLP1)	6.08194	11.7416
Q38ET5	Tb927.9.6460	hypothetical protein, conserved	6.79431	11.5223
Q57V68	Tb927.3.3180	Nucleoporin NUP98	4.35025	10.262
Q38G01	Tb927.9.1410	SUMO-interacting motif-containing protein	4.81713	9.78628
Q57Y35	Tb927.4.4310	Nucleoporin NUP64	7.06158	9.52875
Q586V1	Tb927.2.4230	NUP-1 protein	4.74201	9.45264
Q38A10	Tb927.10.9650	Nucleoporin NUP152	5.0676	9.18434
Q583W5	Tb927.4.2880	Nucleoporin NUP225	4.87759	9.14371
Q38FF7	Tb927.9.3760	Nucleoporin GLE2	3.78726	9.03303
Q57Y62	Tb927.7.6670	hypothetical protein, conserved	5.92805	8.99941
Q38AQ7	Tb927.10.7060	Nucleoporin NUP96	5.03288	8.56275
Q581B5	Tb927.8.3950	hypothetical protein, conserved	5.4272	8.53964
Q386L6	Tb927.11.2950	Nucleoporin NUP89	3.95216	8.50077
Q387F2	Tb927.11.980	Nucleoporin NUP158	4.5024	8.43801
Q381I7	Tb927.11.15990	Nucleoporin NUP109	5.77776	8.38197
Q38BL8	Tb927.10.3810	Nucleoporin NUP65	3.99843	8.27312
Q38D32	Tb927.9.14240	Nucleoporin NUP82	5.01823	8.05696
Q57XP4	Tb927.7.2300	Nucleoporin NUP132	3.24449	7.7052
Q585F7	Tb927.6.890	hypothetical protein, conserved	4.70788	7.70289
Q584I6	Tb927.4.5200	Nucleoporin NUP62	5.5861	7.33425
Q582Q9	Tb927.3.4760	dynamin-1-like protein	3.45599	7.25029
Q383V1	Tb927.11.9780	Nucleoporin NUP119	5.49855	7.20329
Q38BN5	Tb927.10.3640	nuclear transmembrane protein	5.32585	7.17
Q38AE7	Tb927.10.8170	Nucleoporin NUP144	5.5177	7.15809
Q38DR0	Tb927.9.11150	hypothetical protein, conserved	5.07784	7.13933
Q385W3	Tb927.11.4540	Nucleoporin NUP48	4.22511	7.11522
Q57XK4	Tb927.3.3540	Nucleoporin NUP53b	5.40541	6.91711
Q57X92	Tb927.7.4760	hypothetical protein, conserved	3.40266	6.81533
Q388I4	Tb927.10.14180	protein transport protein SEC13	4.52415	6.74846

NUP110 IP significantly enriched interactors (over untagged control) - continued

UniProt ID	TriTryp ID	Description	$-log_{10}$ Student's t-test p-value	log_2 (tagged/untagged)
Q38C12	Tb927.10.2320	Nucleoporin NUP41	3.72137	6.73405
Q57XE3	Tb927.8.2460	hypothetical protein, conserved	3.35065	6.70225
Q387F3	Tb927.11.970	hypothetical protein, conserved	2.93832	6.39177
Q38FU0	Tb927.9.2220	SUMO1/Ulp2	5.74657	6.38493
Q383Z7	Tb927.11.9320	hypothetical protein, conserved	4.94793	6.33722
Q385L2	Tb927.11.5560	hypothetical protein, conserved	4.02665	6.2611
Q57U40	Tb927.8.8050	Nucleoporin NUP75	3.16413	6.24056
Q381M9	Tb927.11.15560	Nucleoporin NUP53a	3.87782	6.23161
Q386D3	Tb927.11.3790	hypothetical protein, conserved	3.91642	6.03219
Q382M8	Tb927.11.13080	hypothetical protein, conserved	3.32361	5.66999
Q582J8	Tb927.7.5830	hypothetical protein, conserved	3.66063	5.1738
Q38AE8	Tb927.10.8160	hypothetical protein, conserved	5.94554	5.10643
Q38A83	Tb927.10.8910	Nucleoporin NUP181	3.65194	5.07702
Q582R3	Tb927.3.4720	dynamin-1-like protein	3.51272	5.03322
Q57Z74	Tb927.5.3190	hypothetical protein, conserved	4.60178	4.91148
Q585L9	Tb927.6.1920	hypothetical protein, conserved	3.56403	4.90318
Q57V60	Tb927.3.3260	hypothetical protein, conserved	4.30145	4.77298
Q57UY5	Tb927.8.4660	hypothetical protein, conserved	3.43363	4.69182
Q4FKD8	Tb927.11.18680	dynein light chain LC8	2.70859	4.49924
Q57WZ3	Tb927.3.1120	GTP-binding nuclear protein rtb2	4.82583	4.31706
Q57YP5	Tb927.8.6440	RNA-binding protein (RBP20)	5.12896	3.95232
Q38FR1	Tb927.9.2590	hypothetical protein, conserved	4.57717	3.93099
Q386H3	Tb927.11.3380	Ran-binding protein 1	2.6877	3.86423
Q57ZJ7	Tb927.3.3150	hypothetical protein, conserved	3.92573	3.62835
Q383H0	Tb927.11.11080	Nucleoporin NUP149	4.76231	3.50611
Q383G9	Tb927.11.11090	Nucleoporin NUP140	4.08639	3.39356
Q386S5	Tb927.11.2340	hypothetical protein, conserved	3.82124	3.34146
Q583W2	Tb927.4.2850	hypothetical protein, conserved	2.9211	3.25883
Q57YE9	Tb927.8.820	VID27 cytoplasmic protein	2.89878	3.20937

NUP110 IP significantly enriched interactors (over untagged control) - continued

UniProt ID	TriTryp ID	Description	$-\log_{10}$ Student's t-test p-value	$\log_2$ (tagged/untagged)
Q38AJ6	Tb927.10.7680	GTPase activating protein	3.06235	3.20847
Q57YM6	Tb927.8.6250	Nucleoporin NUP76	2.14152	3.13853
Q384L3	Tb927.11.8060	SNARE associated Golgi protein	2.41413	3.12
Q582S7	Tb927.3.4580	hypothetical protein, conserved	2.85737	3.05497
Q386Q0	Tb927.11.2600	hypothetical protein, conserved	4.03664	2.76767
P35315	Tb927.8.1180	vacuolar-type Ca2+-ATPase 1	3.13313	2.73411
Q583S0	Tb927.6.2640	importin alpha subunit	3.01621	2.72712
Q582J1	Tb927.7.5760	mRNA transport regulator MTR2	3.00139	2.35135
Q38G07	Tb927.9.1340	Myosin-like protein 2 (MLP2)	4.006	2.12899
Q586Q9	Tb927.2.4580	UNC119	2.52185	2.11811

SET13 IP significantly enriched interactors (over untagged control)

UniProt ID	TriTryp ID	Description	$-log_{10}$ Student's t-test p-value	log_2 (tagged/untagged)
Q584E3	Tb927.4.2440	SET13	4.24447	7.11086
Q57VU5	Tb927.7.990	chaperone protein DnaJ, putative	2.06949	4.64322

SET15 IP significantly enriched interactors (over untagged control)

UniProt ID	TriTryp ID	Description	$-log_{10}$ Student's t-test p-value	log_2 (tagged/untagged)
Q57ZV3	Tb927.5.2770	SET15	5.19135	13.137
Q57UH7	Tb927.8.5050	OTU-like cysteine protease	4.49346	12.3099
Q388D4	Tb927.10.14700	hypothetical protein, conserved	3.02213	7.88572
Q585P0	Tb927.6.1710	hypothetical protein, conserved	4.92275	7.63556
Q57ZS7	Tb927.5.2080	GMP reductase	2.90688	7.40885
Q38DN9	Tb927.9.11470	60S ribosomal protein L27a	3.00961	6.90772
Q386Y7	Tb927.11.1670	cysteine desulfurase	3.27227	6.90033
Q386I1	Tb927.11.3300	Spindle assembly abnormal 4	3.63865	6.86669
Q38C20	Tb927.10.2240	RNA binding protein	3.00736	6.50689
Q38EZ6	Tb927.9.5590	DNA topoisomerase ii (TOP2)	3.25468	6.42083
Q389X5	Tb927.10.10010	mRNA turnover protein 4 homolog	3.57797	6.20563
Q38CY6	Tb927.9.15210	ribosomal protein L36	3.98462	6.06961
Q386T4	Tb927.11.2250	conserved protein, unknown function	4.93118	5.83651
Q38A76	Tb927.10.8980	hypothetical protein, conserved	6.21198	5.68091
Q381T9	Tb927.11.14960	pumilio/PUF RNA binding protein 7	3.73724	5.66011
Q57UX0	Tb927.8.4820	eukaryotic translation initiation factor 4 gamma 3	3.43404	5.66001
Q386C9	Tb927.11.3850	AMP deaminase	4.46495	5.64093
D7SG81	Tb927.11.3230	60S ribosomal protein L44	4.29375	5.6298
Q385R2	Tb927.11.5060	Mitochondrial small ribosomal subunit Rsm22	3.92785	5.61396
Q586G9	Tb927.6.4970	serine/arginine-rich protein specific kinase SRPK	3.42122	5.54974
Q582F1	Tb927.5.2140	regulator of nonsense transcripts 1	6.18513	5.51517
Q388D6	Tb927.10.14680	ribosome biogenesis protein	3.13061	5.50463
Q38FW8	Tb927.9.1810	60S ribosomal protein L35	3.4727	5.47661
Q383S6	Tb927.11.10030	60S ribosomal protein L29	4.35516	5.47136
Q38D54	Tb927.9.13990	RNA-binding protein	4.24849	5.4077
Q57XZ3	Tb927.4.3890	ATP-dependent RNA helicase	3.29763	5.34227
Q57YV4	Tb927.8.7260	kinetoplast-associated protein	3.70907	5.30335
Q583S9	Tb927.6.740	ATP-dependent DEAH-box RNA helicase	3.51075	5.28101
Q57VA5	Tb927.5.1580	zinc finger CCCH domain containing protein 13	3.54964	5.26218

SET15 IP significantly enriched interactors (over untagged control) - continued

UniProt ID	TriTryp ID	Description	$-log_{10}$ Student's t-test p-value	log_2 (tagged/untagged)
Q384V7	Tb927.11.7140	cell cycle sequence binding phosphoprotein (RBP33)	2.95492	5.24986
Q382K0	Tb927.11.13360	AAA ATPase	5.10819	5.19323
Q57WS6	Tb927.7.1240	Sphingosine kinase	4.67664	5.18177
Q389Y5	Tb927.10.9910	PSP1 C-terminal conserved region	2.50741	5.09675
Q584Q1	Tb927.6.2550	RNA-binding protein	2.0502	5.07847
Q57U27	Tb927.7.270	Ribosome production factor 2 homolog	3.50337	5.07752
Q389B0	Tb927.10.12330	zinc finger protein family member	3.04493	4.97903
Q388N7	Tb927.10.13610	zinc-finger of a C2HC-type/Double zinc ribbon	3.68603	4.92499
Q38EA4	Tb927.9.8720	Fructose-1,6-bisphosphate, cytosolic	2.81419	4.9025
Q580H2	Tb927.8.1510	ATP-dependent DEAD/H RNA helicase	3.84264	4.89523
Q38B98	Tb927.10.5030	ubiquitin/ribosomal protein S27a	2.61783	4.84472
Q585M4	Tb927.6.1870	Eukaryotic translation initiation factor 4E	3.27292	4.7831
Q38E96	Tb927.9.8820	hypothetical protein	3.76301	4.74228
Q38E63	Tb927.9.9290	Polyadenylate-binding protein (PABP)	3.30433	4.70896
Q38AL4	Tb927.10.7500	Fibrillarin	2.30138	4.7049
Q57W71	Tb927.8.3020	Q_MOTIF domain-containing protein	2.70837	4.67721
Q38CZ4	Tb927.9.15060	rRNA processing protein	4.09063	4.67001
Q384T4	Tb927.11.7310	RNA binding protein	3.08847	4.622
Q583E3	Tb927.4.4580	hypothetical protein	2.77331	4.5719
Q386I3	Tb927.11.3280	Kinesin-like protein	4.65668	4.54025
Q384Z1	Tb927.11.6790	Ribosome biogenesis protein BOP1 homolog	4.49999	4.46505
D6XKD1	Tb927.7.2140	hypothetical protein	3.36301	4.45784
Q57W56	Tb927.11.16280	60S ribosomal protein L2	3.20381	4.43985
Q586X7	Tb927.2.3620	GTP-binding elongation factor Tu family	3.28946	4.4312
Q57Y68	Tb927.7.6610	FHA domain-containing protein	3.46503	4.41503
Q384J9	Tb927.11.8200	40S ribosomal protein S26	2.24998	4.41346
Q38B42	Tb927.10.5620	Fructose-bisphosphate aldolase	3.94919	4.35694
Q38AY0	Tb927.10.6260	ATP-dependent DEAD/H RNA helicase	3.07219	4.31092
Q38C15	Tb927.10.2290	chaperone protein DnaJ	3.22005	4.26772

SET15 IP significantly enriched interactors (over untagged control) - continued

UniProt ID	TriTryp ID	Description	$-log_{10}$ Student's t-test p-value	log_2 (tagged/untagged)
Q57V37	Tb927.7.1730	60S ribosomal protein L7	5.53509	4.26439
Q386D1	Tb927.11.3830	hypothetical protein	3.69379	4.24606
Q586X0	Tb927.2.3880	Heterogeneous nuclear ribonucleoprotein H/F	2.1852	4.24504
Q38DU2	Tb927.9.10770	Polyadenylate-binding protein (PABP)	4.16027	4.23051
Q57YL9	Tb927.8.6180	60S ribosomal protein L26	2.44842	4.20526
Q57XA6	Tb927.7.4900	5'-3' exonuclease XRNA	2.33263	4.20182
Q386J9	Tb927.11.3120	Nucleolar GTP-binding protein 1	3.70431	4.17426
Q580D8	Tb927.4.1100	Ribosomal protein L21E (60S)	2.72672	4.15507
Q38C87	Tb927.10.1540	hypothetical protein	3.57026	4.14729
Q584N0	Tb927.2.380	Retrotransposon hot spot (RHS) protein	3.6059	4.12329
Q38AI3	Tb927.10.7810	hypothetical protein	3.18018	4.08699
Q382C4	Tb927.10.3840	60S ribosomal protein L18a	3.48248	4.07294
Q38E42	Tb927.9.9520	hypothetical protein	3.17523	4.06933
Q386J2	Tb927.11.3190.1	LisH domain-containing protein	2.63572	4.05821
Q57VC9	Tb927.5.1340	hypothetical protein	3.54771	4.05029
Q389E2	Tb927.10.12000	iron-sulfur cluster assembly protein	3.03889	4.03155
Q57VP3	Tb927.10.5610	40S ribosomal protein S9	3.82509	4.01799
Q38CW9	Tb927.9.15440	60S ribosomal protein L32	3.52884	3.99987
Q57ZF2	Tb927.3.1590	hypothetical protein	3.69145	3.98869
Q38D76	Tb927.9.13510	Poly(A)-specific ribonuclease PARN-3	2.34631	3.98201
Q57YH1	Tb927.8.590	Carnitine O-palmitoyltransferase	3.57014	3.97275
Q382D5	Tb927.11.14020	RNA-binding protein	4.00412	3.9513
Q582W0	Tb927.3.2470	Pumilio/PUF RNA binding protein 8	3.3002	3.94942
Q57U76	Tb927.5.3900	DUF4110 domain-containing protein	3.67235	3.94064
Q38AE4	Tb927.10.8200	hypothetical protein	2.19665	3.90181
Q4GYQ5	Tb927.1.3180	40S ribosomal protein S11	3.92091	3.90132
Q388L9	Tb927.10.13800	hypothetical protein	3.26954	3.88722
Q384Y9	Tb927.11.6810	Tubulin-tyrsoine ligase-like protein	4.15935	3.8777
Q57XS3	Tb927.7.7030	hypothetical protein	2.42791	3.87299

SET15 IP significantly enriched interactors (over untagged control) - continued

UniProt ID	TriTryp ID	Description	$-log_{10}$ Student's t-test p-value	log_2 (tagged/untagged)
Q389K1	Tb927.10.11390	60S ribosomal protein L6	4.05386	3.86197
Q57XS5	Tb927.7.7050	hypothetical protein	2.9185	3.8541
Q38EE3	Tb927.9.8200	Pescadillo homolog	3.53039	3.84321
Q38AM9	Tb927.10.7330	40S ribosomal protein S24	2.14687	3.83504
Q388C7	Tb927.10.14770	Associated kinase of Tb14-3-3	3.33821	3.8304
Q38A09	Tb927.10.9660	hypothetical protein	2.81291	3.82221
Q57ZH2	Tb927.7.5170	60S ribosomal protein L23a	4.1092	3.82092
Q57V56	Tb927.3.3300	hypothetical protein	4.00826	3.81542
Q38FD1	Tb927.9.4080	DksA C4-type domain-containing protein	3.4292	3.81003
Q388E0	Tb927.10.14630	Fibrillarin	2.85126	3.79445
Q383J8	Tb927.11.10810	hypothetical protein	3.7876	3.78787
Q583H9	Tb927.4.3850	WD_REPEATS_REGION domain-containing protein	2.31308	3.74813
Q38EQ1	Tb927.9.6870	RNA-binding protein	5.07229	3.73756
Q388S9	Tb927.10.13180	Nrap protein	4.18907	3.72577
Q38FQ5	Tb927.9.2650	hypothetical protein	4.92438	3.72512
Q385B8	Tb927.11.6510	40S ribosomal protein S21	3.36046	3.71635
Q584N3	Tb927.2.280	Retrotransposon hot spot (RHS) protein	2.23889	3.70949
Q381E6	Tb927.11.16400	Kinetoplast DNA-associated protein	2.7305	3.69274
Q382F6	Tb927.11.13810	ATP-dependent DEAD/H RNA helicase	4.08181	3.69083
Q38AU3	Tb927.10.6630	ATP-dependent DEAD/H RNA helicase HEL64	2.5265	3.67671
Q383D5	Tb927.11.11430	Cullin 1	2.98634	3.67634
Q584T5	Tb927.6.2210	hypothetical protein	2.16107	3.6613
Q583R3	Tb927.6.2710	RING-type domain-containing protein	2.38209	3.66077
Q38CD1	Tb927.10.1080	40S ribosomal protein S23	3.49435	3.64875
Q383V8	Tb927.10.13500	Ribosomal protein	3.54214	3.63036
Q387J8	Tb927.11.530	RNA-binding protein	3.52531	3.60473
Q4GYR6	Tb927.1.3070	hypothetical protein	3.35987	3.5893
Q383K0	Tb927.11.10790	40S ribosomal protein SA	3.76721	3.57936
Q38EI9	Tb927.9.7590	60S ribosomal protein L11	3.14285	3.56804

SET15 IP significantly enriched interactors (over untagged control) - continued

UniProt ID	TriTryp ID	Description	$-log_{10}$ Student's t-test p-value	log_2 (tagged/untagged)
Q585W5	Tb927.6.3670	hypothetical protein	3.20473	3.56031
Q389G5	Tb927.10.11760	Pumillio RNA binding protein	2.00216	3.53919
Q585L0	Tb927.4.3400	hypothetical protein, conserved	3.24737	3.53701
Q38FD9	Tb927.9.3990	40S ribosomal protein S7	4.80274	3.53245
Q385Y8	Tb927.11.4290	40S ribosomal protein S12	2.34993	3.48545
Q587G6	Tb927.2.2950	hypothetical protein	2.07158	3.48127
Q386S5	Tb927.11.2340	hypothetical protein	3.9396	3.4788
Q388M6	Tb927.10.13730	60S ribosomal protein L7	4.07673	3.46676
Q386K4	Tb927.11.3070	WD_REPEATS_REGION domain-containing protein	2.98843	3.46486
Q584V1	Tb927.6.2050	Ribosome biogenesis regulatory protein	3.71135	3.44493
Q389M4	Tb927.10.11150	hypothetical protein	4.66972	3.43146
Q581A2	Tb927.8.3820	hypothetical protein	3.77594	3.42405
Q386W1	Tb927.11.1980	hypothetical protein	2.99391	3.40884
Q38D96	Tb927.9.13280	RNA-binding protein	2.583	3.40112
Q387P8	Tb927.10.16170	Potassium voltage-gated channel	2.46185	3.39853
Q585J4	Tb927.4.3560	Serine/threonine-protein phosphatase	2.8735	3.38731
Q381D1	Tb927.11.16550	hypothetical protein	3.26232	3.38087
Q584T3	Tb927.6.2230	hypothetical protein	2.53357	3.36381
Q57W29	Tb927.5.840	Kri1_C domain-containing protein	3.83681	3.35617
Q586Q9	Tb927.2.4580	UNC119	3.06884	3.35043
Q38A77	Tb927.10.8970	kinetoplast-associated protein 4 isoform 2	3.29922	3.31246
Q385T6	Tb927.10.14580	60S ribosomal protein L17	4.30583	3.30292
Q38FU7	Tb927.9.2120	hypothetical protein	3.70276	3.30169
Q38A89	Tb927.10.8850	hypothetical protein	4.57183	3.29137
Q389P5	Tb927.10.10850	Argonaute-like protein	2.60182	3.28613
Q38C06	Tb927.10.2380	hypothetical protein	4.20126	3.28148
Q381W0	Tb927.11.14750	PSP1 C-terminal domain-containing protein	2.18462	3.27147
Q57VM1	Tb927.8.1330	60S ribosomal protein L7a	3.04956	3.26175
Q586I3	Tb927.6.5110	Damage-specific DNA binding protein	3.42713	3.25236

SET15 IP significantly enriched interactors (over untagged control) - continued

UniProt ID	TriTryp ID	Description	$-log_{10}$ Student's t-test p-value	log_2 (tagged/untagged)
Q389I7	Tb927.10.11540	40S ribosomal protein S3	3.3862	3.25215
Q585R0	Tb927.6.1500	Alkyl-dihydroxyacetone phosphate synthase	2.66972	3.24953
Q38B59	Tb927.10.5450	WSD domain-containing protein	2.16	3.24211
Q382Z8	Tb927.11.11820	40S ribosomal protein S17	4.03675	3.23999
Q389Z2	Tb927.10.9840	Chaperone protein DNAJ	4.18974	3.23137
Q57VZ6	Tb927.8.4390	Translation initiation factor eIF-2B beta subunit	2.70249	3.21469
Q38DT1	Tb927.9.10920	kinetoplastid kinetochore protein 3 (KKT3)	2.52493	3.20547
Q38AL9	Tb927.10.7440	ATP-dependent DEAD/H RNA helicase	2.98255	3.20171
Q387E2	Tb927.11.1080	hypothetical protein	3.9101	3.19574
Q57XC9	Tb927.8.2600	WD_REPEATS_REGION domain-containing protein	3.01492	3.17189
Q386F2	Tb927.11.3590	40S ribosomal protein S4	3.41196	3.14524
Q38D68	Tb927.9.13610	Helicase	2.63388	3.14487
Q386Y8	Tb927.11.1660	vesicular transport protein (CDC48 homologue)	2.93632	3.13838
Q57YK4	Tb927.8.6030	60S ribosomal protein L12	3.90584	3.11987
Q57W53	Tb927.5.1080	RRM domain-containing protein	3.11179	3.08933
Q57UC8	Tb927.7.3810	CSD domain-containing protein	2.64629	3.08698
Q384B1	Tb927.11.9170	DNA topoisomerase	5.00839	3.08673
Q580Z5	Tb927.8.3750	Nucleolar protein	4.27428	3.08103
Q389D3	Tb927.10.12100	RNA-binding protein 7B	2.23506	3.06525
Q386V3	Tb927.11.2050	60S acidic ribosomal protein P0	2.89859	3.04556
Q38A41	Tb927.10.9330	hypothetical protein	2.63072	3.03462
Q385C6	Tb927.11.6430	hypothetical protein	2.0276	3.02604
Q57VV0	Tb927.7.1040	40S ribosomal protein S16	3.21614	3.01444
Q388D3	Tb927.10.14600	40S ribosomal protein S2	3.31631	3.01003
Q38B16	Tb927.10.5880	hypothetical protein	2.28198	2.98036
Q38AW9	Tb927.10.220	60S ribosomal protein L37a (60S ribosomal proteins L37)	2.11727	2.97034
Q57UM7	Tb927.7.3970	hypothetical protein	2.33563	2.9664
Q381T5	Tb927.11.15000	survival of motor neuron (SMN)-like protein	2.0564	2.96548
Q57XX0	Tb927.10.2840	40S ribosomal protein S25	2.95092	2.95547

SET15 IP significantly enriched interactors (over untagged control) - continued

UniProt ID	TriTryp ID	Description	$-\log_{10}$ Student's t-test p-value	$\log_2$ (tagged/untagged)
Q580A5	Tb927.4.770	Ubiquitin-protein ligase	2.04869	2.94927
Q384Z8	Tb927.11.6710	TPR_REGION domain-containing protein	2.79763	2.93361
Q57VV2	Tb927.7.1060	hypothetical protein	2.26167	2.92866
Q385E9	Tb927.11.6180	60S ribosomal protein L28	3.40982	2.92177
Q385D9	Tb927.11.6300	40S ribosomal protein S5	3.48865	2.91507
Q585U7	Tb927.2.5240	WD_REPEATS_REGION domain-containing protein	2.80848	2.91294
Q57VG4	Tb927.5.760	PSP1 C-terminal domain-containing protein	2.97773	2.9101
Q57XN5	Tb927.7.2390	hypothetical protein	3.82771	2.90552
Q8IFI8	Tb927.1.120	retrotransposon hot spot protein 4 (RHS4)	3.52319	2.891
Q381S8	Tb927.11.15070	hypothetical protein	2.97025	2.88682
Q38B69	Tb927.10.5330	40S ribosomal protein S18	3.16758	2.8793
Q385Z8	Tb927.11.4180	PSP1 C-terminal domain-containing protein	3.50981	2.83662
Q57XN5	Tb927.7.2390	hypothetical protein	3.82771	2.90552
Q8IFI8	Tb927.1.120	retrotransposon hot spot protein 4 (RHS4)	3.52319	2.891
Q381S8	Tb927.11.15070	hypothetical protein	2.97025	2.88682
Q38B69	Tb927.10.5330	40S ribosomal protein S18	3.16758	2.8793
Q385Z8	Tb927.11.4180	PSP1 C-terminal domain-containing protein	3.50981	2.83662
Q388B1	Tb927.10.14950	RNA binding protein	2.10442	2.83194
Q38FQ8	Tb927.9.2620	DUF4460 domain-containing protein	4.01138	2.80817
Q38D98	Tb927.9.13240	hypothetical protein	2.32729	2.80118
Q57WA3	Tb927.3.3670	RNA-binding protein	2.91958	2.79741
Q4GZ47	Tb927.1.1720	PPIase cyclophilin-type domain-containing protein	3.71482	2.79029
Q38A78	Tb927.10.8960	hypothetical protein	3.22946	2.78949
Q57VM8	Tb927.8.1270	hypothetical protein	3.17046	2.76994
Q38BK6	Tb927.10.3940	40S ribosomal protein S3a	3.43681	2.75714
Q38A85	Tb927.10.8890	Kinetoplast DNA-associated protein	2.37494	2.75642
Q57V04	Tb927.7.2070	heat shock protein DNAJ	4.20609	2.74908
Q38DS7	Tb927.9.10960	ATP-dependent DEAD/H RNA helicase	4.87165	2.74222
Q57ZC0	Tb927.3.1910	SLBP_RNA_bind domain-containing protein	2.27418	2.736

SET15 IP significantly enriched interactors (over untagged control) - continued

UniProt ID	TriTryp ID	Description	$-\log_{10}$ Student's t-test p-value	$\log_2$ (tagged/untagged)
Q385U8	Tb927.11.4690	Mitochondrial DNA polymerase I protein B	3.65971	2.70458
Q387Q3	Tb927.10.16120	inosine-5'-monophosphate dehydrogenase	4.43817	2.704
Q57Z68	Tb927.5.3250.1	WW domain-containing protein	2.58578	2.69314
Q38BJ4	Tb927.10.4060	ANK_REP_REGION domain-containing protein	2.09685	2.68063
Q57ZI6	Tb927.7.5030	hypothetical protein	2.01053	2.67893
Q586R7	Tb927.2.4710	RNA-binding protein	3.08724	2.67873
Q585W7	Tb927.6.3690	Pre-mRNA cleavage complex II Clp1-like, conserved	2.40348	2.65934
Q38C70	Tb927.10.1720	ATP-dependent DEAD/H RNA helicase	2.50131	2.65842
Q38CY8	Tb927.9.15130	60S ribosomal protein L5	3.21633	2.65776
Q388B3	Tb927.10.14930	RNA binding protein	3.65408	2.63675
Q580I2	Tb927.8.1410	hypothetical protein	2.1087	2.61964
Q38BQ9	Tb927.10.3370	60S acidic ribosomal protein P2	3.24002	2.61218
Q585A4	Tb927.6.1430	MATH domain-containing protein	2.69023	2.57222
Q389Z8	Tb927.10.9780	ATP-dependent DEAD/H RNA helicase	3.06332	2.56546
Q583H8	Tb927.4.3840	Nucleolar protein	2.7113	2.53828
Q586C2	Tb927.10.15120	40S ribosomal protein S13	2.65386	2.53534
Q383R3	Tb927.11.10160	60S ribosomal protein L22	4.02774	2.53296
Q580V1	Tb927.3.5240	hypothetical protein	2.70324	2.53034
Q38F23	Tb927.9.5320	Nucleolar RNA binding protein	4.15374	2.51347
Q38EY6	Tb927.9.5690	60S acidic ribosomal protein	2.95999	2.51149
Q581X8	Tb927.8.2040	DUF1767 domain-containing protein	2.13478	2.50631
Q586U9	Tb927.2.4280	Dual specificity protein phosphatase	2.03869	2.50609
Q57VA4	Tb927.5.1590	hypothetical protein	2.9407	2.48611
Q57XW2	Tb927.3.1290	Cullin 4B	2.13664	2.47057
Q57YB5	Tb927.7.3050	hypothetical protein	2.40919	2.4608
Q383P6	Tb927.11.10330	hypothetical protein	2.75613	2.4545
Q38BC7	Tb927.10.4740	Nucleolar RNA-binding protein	3.05298	2.45369
Q4GYF0	Tb927.1.4280	hypothetical protein	2.53048	2.44099
D6XDN4	Tb927.3.3270	ATP-dependent 6-phosphofructokinase (ATP-PFK)	3.33743	2.43232

SET15 IP significantly enriched interactors (over untagged control) - continued

UniProt ID	TriTryp ID	Description	$-\log_{10}$ Student's t-test p-value	$\log_2$ (tagged/untagged)
Q385X2	Tb927.11.4450	Alba domain-containing protein	3.38772	2.40524
Q38AC1	Tb927.10.8430	40S ribosomal protein S12	3.25531	2.39142
Q387J6	Tb927.11.550	hypothetical protein	3.25812	2.38947
Q57VZ0	Tb927.8.4450	RNA-binding protein	2.37816	2.37347
Q388E8	Tb927.10.14550	ATP-dependent DEAD/H RNA helicase	3.58184	2.3714
Q38C52	Tb927.10.1900	DNA topoisomerase IA	3.48388	2.36735
Q57YW0	Tb927.8.3530	glycerol-3-phosphate dehydrogenase [NAD+], glycosomal	4.54295	2.36698
Q385D7	Tb927.11.6320	C3H1-type domain-containing protein	2.67242	2.36186
Q38EH3	Tb927.9.7820	hypothetical protein	3.86725	2.35923
Q38AF8	Tb927.10.8060	SET domain-containing protein (SET8)	2.31375	2.34632
Q383B0	Tb927.11.11700	NTP_transferase domain-containing protein	4.16059	2.3409
Q580W2	Tb927.3.5130	Exonuclease domain-containing protein	2.9251	2.33829
Q383C3	Tb927.11.11570	hypothetical protein	2.7362	2.32769
Q57ZJ9	Tb927.3.3130	SAM domain-containing protein	2.43051	2.31054
Q4FKA6	Tb11.1400.1	Translation initiation factor eIF2B delta subunit	2.3716	2.30415
Q4FKG9	Tb11.0290	40S ribosomal protein S14 (40s ribosomal protein S14)	2.61375	2.2664
Q38B66	Tb927.10.5370	40S ribosomal protein S10	2.71203	2.26611
Q38AD1	Tb927.10.8330	PSP1 C-terminal domain-containing protein	2.0349	2.22758
Q57WI5	Tb927.7.3700	hypothetical protein	2.61439	2.21652
Q38AY4	Tb927.10.6220	5'-3' exoribonuclease XRND	2.03223	2.21397
Q387C7	Tb927.11.1250	hypothetical protein	4.40491	2.17145
Q389R6	Tb927.10.10620	hypothetical protein	2.9099	2.1467
Q386S9	Tb927.11.2300	Eukaryotic peptide chain release factor subunit 1	2.22266	2.11068
Q38B00	Tb927.10.6060	Universal minicircle sequence binding protein (UMSBP)	3.96851	2.08543
Q57XY3	Tb927.3.1500	Variant surface glycoprotein (VSG)-related	3.01641	2.08463
Q584U2	Tb927.6.2140	hypothetical protein	3.74363	2.07661
Q385I3	Tb927.11.5850	RNA-binding protein	2.33252	2.06997
Q586P0	Tb927.2.6070	hypothetical protein	2.23715	2.05721
Q386Q9	Tb927.11.2510	NUC173 domain-containing protein	3.22904	2.05103

SET15 IP significantly enriched interactors (over untagged control) - continued

UniProt ID	TriTryp ID	Description	$-log_{10}$ Student's t-test p-value	log_2 (tagged/untagged)
Q57WY4	Tb927.3.1010	hypothetical protein	3.32166	2.01911

SET20 IP significantly enriched interactors (over untagged control)

UniProt ID	TriTryp ID	Description	$-log_{10}$ Student's t-test p-value	log_2 (tagged/untagged)
Q57XE0	Tb927.8.2490	SET20	5.95491	9.11766
Q38C15	Tb927.10.2290	chaperone protein DnaJ, putative	3.36358	2.56159
Q38DH5	Tb927.9.12300	replication factor C, subunit 3, putative	2.85488	2.17278

SET23 IP significantly enriched interactors (over untagged control)

UniProt ID	TriTryp ID	Description	$-log_{10}$ Student's t-test p-value	log_2 (tagged/untagged)
Q57YP8	Tb927.8.6470	SET23	5.85826	13.5365
Q57UQ9	Tb927.7.4290	Nuclear distribution protein C homolog	5.5979	7.94808
Q382B5	Tb927.11.14220	Regulator of ESAG9-1	3.87279	3.76776
Q38EI4	Tb927.9.7690	SUMO-interacting motif-containing protein	3.8291	3.70809
Q586Q9	Tb927.2.4580	UNC119	3.28072	3.15086
Q57XP8	Tb927.7.2260	SEP domain containing protein, putative	2.7166	2.00686

SET25 IP significantly enriched interactors (over untagged control)

UniProt ID	TriTryp ID	Description	$-log_{10}$ Student's t-test p-value	log_2 (tagged/untagged)
Q38FZ5	Tb927.9.1510	SET25	5.11563	12.386

Supplemental Table S5

Supplemental Table S5. Information about key proteins enriched with putative chromatin regulators in *T. brucei*

Identified interactors shared between two or more proteins are presented as a network followed by those that are uniquely associated with each protein in that network.

[1] RIT-seq data from Alsford et al (2011). Scoring matrix key: 0-0-0-0 group - significant loss of fitness in all four experiments; 0-0-1-0 group - significant loss of fitness in BF experiments only; 1-1-0-0 group - significant loss of fitness in PF experiments only; 1-1-1-0 group - significant loss of fitness in the differentiation (BF to PF) experiment only; 1-1-1-1 group - no significant loss of fitness in any experiment; none of the above - other groups.

[2] The estimated probability of the template to be (at least partly) homologous to a query sequence is the key criterion for deciding whether a template HMM is actually homologous or just a high-scoring chance hit. Scores >95%: homology is nearly certain. Serious consideration should be given to hits whenever (1) it has >50% probability, or (2) it has >30% probability and is among the top three hits (Soding et al., 2005). Detected homologies were inspected manually and, where relevant, hits were checked by specific BLAST searches with the same *T. brucei* protein against all *S. cerevisiae* or *S. pombe* proteins.

Proteins marked as nuclear enriched were analyzed by mass spectrometry in PF cells by Goos et al. (2017).

NRM – no relevant match

**CRD1-SET27 network**							
**UniProt ID**	**TriTryp ID**	**Gene name/description**	**[1]RIT-seq scoring matrix**	**Localization**	**PDB entry**	**HHpred domain**	**[2]HHpred domain probability %**
Q57X70	Tb11.v5.0267	CRD1	1-1-1-1	nuclear (this study)	4O42_A	Chromodomain CHD1	97.38
Q38D80	Tb927.9.13470	SET27	1-1-1-1	nuclear/cytoplasmic (this study);<br>nuclear enriched (mass spectrometry)	5JLB_A	Histone lysine N-methyltransferase	99.06
Q4GYF3	Tb927.1.4250	uncharacterized	1-1-1-1	nuclear enriched (mass spectrometry)	NRM	NRM	NRM
Q382F5	Tb927.11.13820	uncharacterized	other	nuclear enriched (mass spectrometry)	NRM	NRM	NRM
Q582U8	Tb927.3.2350	uncharacterized;<br>similar to Bye1 (*S. cerevisiae*)	other	-	5XFO_A	PHD Finger domain	97.18
Q382Z7	Tb927.11.11840	uncharacterized;<br>similar to Chp2 (*S. pombe*)	1-1-1-1	-	6FTO_B	Chromoshadow domain	94.75
D6XIZ6	Tb927.7.4650	JBP2	1-1-1-1	-	4LT5_A<br>6G7E_B	Tet-like Dioxygenase;<br>ATPase Helicase	100<br>97.97
Q586X9	Tb927.2.3580	TFIIS2-1;<br>also detected with EAF6 & TFIIS2-2	1-1-1-1	nuclear enriched (mass spectrometry)	1PQV_S<br>2M1H_A	TFIIS;<br>PWWP domain	100<br>99.22

Enriched in YFP-CRD1 affinity selections

UniProt ID	TriTryp ID	Gene name/description	1RIT-seq scoring matrix	Localization	PDB entry	HHpred domain	2HHpred domain probability %
Q38FV1	Tb927.9.2070	uncharacterized; also detected with HDAC1	other	-	NRM	NRM	NRM
Q387L4	Tb927.11.370	RAP1; also detected with HDAC3 & TRF	0-0-1-0	nuclear enriched (mass spectrometry); telomeric (Nanavaty et al., 2017)	3AL2_A	BRCT domain	94.26
Q57YU2	Tb927.8.7400	RPB1; also detected with SET26 & TFIIS2-2	0-0-0-0	nuclear enriched (mass spectrometry)	4XPZ_A	Rpb1 RNAPII subunit	96.21
Q580S5	Tb927.3.5500	RPB3; also detcted with SET26 & TFIIS2-2	other	nuclear enriched (mass spectrometry)	1TWF_C	Rpb3 RNAPII subunit	100

Enriched in YFP-SET27 affinity selections

UniProt ID	TriTryp ID	Gene name/description	[1]RIT-seq scoring matrix	Localization	PDB entry	HHpred domain	[2]HHpred domain probability %
Q38BT9	Tb927.10.3070	uncharacterized	other	nuclear enriched (mass spectrometry)	4QQ6_A	Tudor Domain	47.7
Q57Z72	Tb927.5.3210	SUMO; also detected with BDF3, BDF6 & HAT2	other	-	3PGE_A	SUMO (small ubiquitin like protein)	99.03
Q38EP6	Tb927.9.6920	uncharacterized	1-1-1-1	-	NRM	NRM	NRM
P86938	Tb927.11.13640	JPB1	1-1-1-1	-	2XSE_A<br>5DEU_A	Thymine Dioxygenase;<br>Tet-like	100<br>96.4
Q389E6	Tb927.10.11960	PNUTS; also detected with TFIIS2-2	1-1-1-1	-	NRM	NRM	NRM
Q57W24	Tb927.5.790	Casein kinase I isoform 1	other	-	6GZD_A	Casein Kinase 1	100
Q57VC7	Tb927.5.1360	NDRT; also detected with BDF4	1-1-1-1	-	2F62_B	Nucleoside 2-deoxyribosyltransferase	99.87
Q586H7	Tb927.6.5050	V-type ATPase	1-1-1-1	-	6O7U_c	V-type proton ATPase subunit	99.95
Q388E9	Tb927.10.14530	RPN8	other	-	6EPF	26S Proteasome subunit	100
Q57UB5	Tb927.5.3510	SMC3; also detected with HDAC3	other	nuclear enriched (mass spectrometry)	6WG3_B	SMC Cohesin/Condensin	100
Q38E40	Tb927.9.9550	ERBP2	0-0-1-0	-	NRM	NRM	NRM
Q38C86	Tb927.10.1550	RPN5	0-0-1-0	-	3JCK_B	26S Proteasome subunit	100

BDF1-BDF4 network

UniProt ID	TriTryp ID	Gene name/description	[1]RIT-seq scoring matrix	Localization	PDB entry	HHpred domain	[2]HHpred domain probability %
Q38AE9	Tb927.10.8150	BDF1	1-1-1-1	nuclear (this study); nuclear enriched (mass spectrometry)	6DNE_B<br>2NCZ_A	Bromo domain;<br>ET Domain	99.0<br>97.4
Q57UR8	Tb927.7.4380	BDF4	other	nuclear/cytoplasmic (this study)	6DNE_B<br>2E61_A	Bromo domain;<br>Znf-CW PWWP	97.8<br>93.24
Q383J7	Tb927.11.10820	Casein kinase II subunit beta;<br>also detected with BDF7	other	-	4DGL_A	Casein Kinase II subunit beta	100

Enriched in YFP-BDF1 affinity selections

UniProt ID	TriTryp ID	Gene name/description	[1]RIT-seq scoring matrix	Localization	PDB entry	HHpred domain	[2]HHpred domain probability %
Q389A2	Tb927.10.12410	uncharacterized	other	-	1G0S_A	ADP-ribose pyrophosphatase	99.72
Q57WL5	Tb927.8.8150	C2 domain;<br>also detected with SET26	0-0-0-0	-	6FLJ_A	C2 domain (Calcium binding, membrane bound)	99.2

Enriched in YFP-BDF4 affinity selections

UniProt ID	TriTryp ID	Gene name/description	[1]RIT-seq scoring matrix	Localization	PDB entry	HHpred domain	[2]HHpred domain probability %
Q38D21	Tb927.9.14430	Casein kinase II; also detected with BDF7, SET26 & ZCW1	other	-	5XVU_B	Casein Kinase CKII	100
Q57YY8	Tb927.8.3250	Dynein Heavy Chain	0-0-0-0	flagellar (Dean et al., 2016)	6SC2_B	Dynein Heavy Chain	100
Q57V45	Tb927.3.3410	Aspartyl amino peptidase	1-1-1-0	-	4EME_A	Aspartyl Amino peptidase	100
Q57VC7	Tb927.5.1360	NDRT	1-1-1-1	-	2F62_B	Nucleoside 2-deoxyribosyltransferase	99.87
Q57UC1	Tb927.5.3450	eIF2A	1-1-1-0	-	3WJ9_A	Eukaryotic translation initiation factor 2A	100

BDF3-BDF5-HAT2 network

UniProt ID	TriTryp ID	Gene name/description	[1]RIT-seq scoring matrix	Localization	PDB entry	HHpred domain	[2]HHpred domain probability %
Q383S2	Tb927.11.10070	BDF3	other	nuclear (this study); nuclear enriched (mass spectrometry)	4QB3_A<br>2NCZ_A	Bromo domain;<br>ET Domain	99.11<br>98.72
Q382J7	Tb927.11.13400	BDF5;<br>also detected with ZCW1	0-0-1-0	nuclear (this study); nuclear enriched (mass spectrometry)	2R0Y_A<br>2F5J_B	Tandem Bromo Domains;<br>MRG Domain	99.7<br>96.87
Q383C5	Tb927.11.11530	HAT2;<br>similar to KAT8 (*H. sapiens*) and Esa1 (*S. cerevisiae*)	1-1-1-1	nuclear (this study); nuclear enriched (mass spectrometry)	3TO7_A	Esa1 Histone acetyltransferase	100
Q4FKD8	Tb927.11.18680	Dynein Light Chain	other	-	5E0L_A	Dynein Light Chain	99.97
Q580M8	Tb927.3.4140	uncharacterized	other	-	4ZZZ_B	Poly ADP Ribose Polymerase (PARP)	57.11
Q584F3	Tb927.4.2340	uncharacterized	0-0-0-0	nuclear enriched (mass spectrometry)	5FUR_G	Bromo domain (TFIID-TFIIA)	78.45
Q585E0	Tb927.6.1070	uncharacterized	other	nuclear enriched (mass spectrometry)	NRM	NRM	NRM
Q57Y87	Tb927.7.2770	uncharacterized	other	nuclear enriched (mass spectrometry)	NRM	NRM	NRM
Q38D92	Tb927.9.13320	uncharacterized;<br>also detected with SET26	other	nuclear enriched (mass spectrometry);	5XZW_A	FHA domain (Phospho-Threonine-Binding)	96.3
Q385P5	Tb927.11.5230	uncharacterized;<br>also detected with HDAC3 & ZCW1	0-0-0-0	nuclear enriched (mass spectrometry); nuclear (D'Archivio and Wickstead, 2017)	1UZ3_A	EMSY ENT domain (Chromo shadow binding)	95.93

Enriched in YFP-BDF3 affinity selections

UniProt ID	TriTryp ID	Gene name/description	[1]RIT-seq scoring matrix	Localization	PDB entry	HHpred domain	[2]HHpred domain probability %
Q57Z72	Tb927.5.3210	SUMO;<br>also detected with BDF6, HAT2 & SET27	other	-	3PGE_A	SUMO (Small Ubiquitin like protein)	99.03
Q38BR9	Tb927.10.3280	L38;<br>also detected with BDF5	0-0-0-0	-	5UMD_d	60S ribosomal protein	99.94
Q383E9	Tb927.11.11290	HSP70;<br>also detected with BDF5, BDF7, SET26, ZCW1, TFIIS2-2 & DOT1A	1-1-1-0	nuclear enriched (mass spectrometry)	5FPN_A	Heat Shock 70 protein	100
Q4GYZ8	Tb927.1.2230	SMP1-1;<br>also detected with BDF5	1-1-1-1	-	2FE0_A	Small myristoylated protein 1	100

Enriched in YFP-BDF5 affinity selections

UniProt ID	TriTryp ID	Gene name/description	[1]RIT-seq scoring matrix	Localization	PDB entry	HHpred domain	[2]HHpred domain probability %
Q38BR9	Tb927.10.3280	L38;<br>also detected with BDF3	0-0-0-0	-	5UMD_d	60S ribosomal protein	99.94
Q383E9	Tb927.11.11290	HSP70;<br>also detected with BDF3, BDF7, SET26, ZCW1, TFIIS2-2 & DOT1A	1-1-1-0	nuclear enriched (mass spectrometry)	2FE0_A	Heat Shock 70 protein	100
Q4GYZ8	Tb927.1.2230	SMP1-1;<br>also detected with BDF3	1-1-1-1	-	5FPN_A	Small myristoylated protein 1	100

Enriched in YFP-HAT2 affinity selections

UniProt ID	TriTryp ID	Gene name/description	[1]RIT-seq scoring matrix	Localization	PDB entry	HHpred domain	[2]HHpred domain probability %
Q57Z72	Tb927.5.3210	SUMO;<br>also detected with BDF3, BDF6 & SET27	other	-	3PGE_A	SUMO (Small Ubiquitin like protein)	99.03
Q38BI9	Tb927.10.4110	L30	other	-	5UMD_6	60S Ribosomal protein	99.87
Q9N942	Tb927.1.420	RHS5	-	-	6K0R_B	RuvB-like AAA-ATPase	96.11
Q38BE4	Tb927.10.4570	Elongation factor 2	0-0-0-0	-	6ID1_C	Splicing factor	100

BDF2-HDAC3 network

UniProt ID	TriTryp ID	Gene name/description	[1]RIT-seq scoring matrix	Localization	PDB entry	HHpred domain	[2]HHpred domain probability %
Q38AM1	Tb927.10.7420	BDF2;<br>similar to Bdf1 (*S. cerevisiae*);<br>also detected with ZCW1	1-1-1-1	nuclear (this study)	6DNE_B<br>2ZD7_A	Bromo domain;<br>NAP domain (histone chaperone)	99.29<br>83.7
Q586J9	Tb927.2.2190	HDAC3;<br>also detected with TRF & ZCW1	0-0-0-0	nuclear (this study);<br>nuclear in BF and PF (Wang et al., 2010)	2VQM_A	HDAC	100
Q582V9	Tb927.3.2460	uncharacterized	0-0-0-0	-	5IJO_N	Nuclear pore (Nup155)	56.06
Q586Z9	Tb927.6.4330	uncharacterized;<br>also detected with TRF	0-0-0-0	nuclear enriched (mass spectrometry);<br>telomeric<br>(Reis et al., 2018)	NRM	NRM	NRM
Q38FD7	Tb927.9.4000	uncharacterized;<br>also detected with TRF	-	nuclear enriched (mass spectrometry);<br>telomeric<br>(Reis et al., 2018)	NRM	NRM	NRM
Q38EB9	Tb927.9.8520	uncharacterized	other	-	5Y27_A	DNA pol epsilon	91.92
Q388W1	Tb927.10.12850	TRF; telomere-repeat bining protein	0-0-0-0	nuclear (this study);<br>nuclear enriched (mass spectrometry);<br>telomeric<br>(Jehi et al., 2014; Li et al., 2005)	1GV2_A	MYB domain DNA binding	80.11
Q57XY9	Tb927.3.1560	TIF2;<br>also detected with TRF	other	telomeric<br>(Jehi et al., 2014)	NRM	NRM	NRM
Q383U2	Tb927.11.9870	TelAP1;<br>also detected with TRF	1-1-1-1	nuclear enriched (mass spectrometry);<br>telomeric<br>(Reis et al., 2018)	4B0Z_A	Proteasome subunit	56.61
Q385L3	Tb927.11.5550	DNA polymerase theta (POLQ);<br>also detected with TRF	1-1-1-1	nuclear enriched (mass spectrometry);<br>telomeric<br>(Reis et al., 2018)	4X0Q_B	DNA polymerase theta	100
Q38BZ3	Tb927.10.2520	PrimPol-like protein 2;<br>also detected with TRF	0-0-1-0	nuclear enriched (mass spectrometry)	5L2X_B	DNA Primase Polymerase	100
Q57W25	Tb927.5.800	Casein kinase I, isoform 2	-	-	5CYZ_A	Casein kinase I homolog HRR25	100
Q38D76	Tb927.9.13510	PARN3	1-1-1-1	-	3D45_B	Poly(A)-specific ribonuclease PARN	100
Q57WS6	Tb927.7.1240	Sphingosine kinase	other	-	4WER_A	Diacylglycerol/lipid kinase	100

Enriched in YFP-BDF2 affinity selections

UniProt ID	TriTryp ID	Gene name/description	[1]RIT-seq scoring matrix	Localization	PDB entry	HHpred domain	[2]HHpred domain probability %
Q57UN4	Tb927.7.4040	Similar to Swc4 (*S. cerevisiae*); also detected with ZCW1	other	nuclear enriched (mass spectrometry)	4IEJ_A	DMAP1, chromatin regulator; SWR1 complex	97.52
Q57ZL0	Tb927.3.3020	actin-related; similar to Arp6 (*S. cerevisiae*); also detected with ZCW1	other	nuclear enriched (mass spectrometry)	4FO0_A	Actin-related protein Chromatin remodelling	100
Q584P9	Tb927.6.2570	actin-related; also detected with ZCW1	other	nuclear enriched (mass spectrometry)	4I6M_B	Actin-related protein Chromatin remodelling	97.36
Q383K6	Tb927.11.10730	SWI/SNF-related helicase; similar to Swr1 (*S. cerevisiae*); also detected with ZCW1	other	nuclear enriched (mass spectrometry)	6FML_G	ATPase Helicase, SWR1/INO80 complexes	100
Q389H2	Tb927.10.11690	YEATS; similar to YEA1; Yaf9 (*S. cerevisiae*); also detected with ZCW1	other	nuclear enriched (mass spectrometry)	6T1I_A	YEATS domain Acetyl-/crotonyl-lysine histone binding	98.21
Q57YH0	Tb927.8.600	Bucentaur/craniofacial development; also detected with ZCW1	other	nuclear enriched (mass spectrometry)	6PX3_S	Histone/nucleosome binding (possible *S. cerevisiae* equivalent SWR1-C Swc5)	53.46
Q389D3	Tb927.10.12100	RBP7B; stumpy driver (Mony et al., 2014)	other	-	5YVG_Y	RRM RNA binding	99.17
Q385T8	Tb927.11.4800	clathrin coat assembly protein	other	-	5OO7_B	Phospholipid binding	100
Q583J3	Tb927.4.2000	ruvB-like DNA helicase; similar to Rvb1/2 (*S. cerevisiae*); also detected with ZCW1	0-0-1-0	-	6H7X_A	RuvB-like ATPase helicase, SWR1/INO80 complexes	99.96
Q580C6	Tb927.4.980	actin-related; similar to Act1 (*S. cerevisiae*); also detected with ZCW1	1-1-1-1	nuclear enriched (mass spectrometry)	4AM6_A	Actin or Actin-related protein, Chromatin remodelling	100
Q38C43	Tb927.10.2000	actin-related; similar to Arp4 (*S. cerevisiae*); also detected with ZCW1	other	nuclear enriched (mass spectrometry)	6GEN_R	Actin-related protein, Chromatin remodelling	100
Q581V4	Tb927.4.1270	ruvB-like DNA helicase; similar to Rvb1/2 (*S. cerevisiae*); also detected with ZCW1	0-0-1-0	-	2C9O_C	RuvB-like ATPase helicase; SWR1/INO80 complexes	100
Q385E0	Tb927.11.6290	HIT zinc finger; similar to Swc6 (*S. cerevisiae*); also detected with ZCW1	1-1-1-1	nuclear enriched (mass spectrometry)	2N95_A<br>6GEJ_S	HIT Zinc Finger; SWR/INO80 complex linked to RuvB	97.12<br>99.4
Q57YW0	Tb927.8.3530	G3PDH	0-0-0-0	glycosomal (Kohl et al., 1996)	1EVY_A	Glycerol-3-phosphate dehydrogenase	100
Q389G9	Tb927.10.11720	ZCW1	other	nuclear (this study); nuclear enriched (mass spectrometry)	6O5W_A	MORC family CW-type zinc finger	97.53
Q57ZS8	Tb927.5.2090	kinesin	0-0-1-0	-	5MIO_C<br>6YUF_A	Kinesin; Cohesin Rad21	100<br>99.9

Enriched in YFP-BDF2 affinity selections – continued

UniProt ID	TriTryp ID	Gene name/description	[1]RIT-seq scoring matrix	Localization	PDB entry	HHpred domain	[2]HHpred domain probability %
Q389D9	Tb927.10.12030	uncharacterized	1-1-1-1	nuclear enriched (mass spectrometry)	4AN6_A	Protease inhibitor	28.6
Q384I8	Tb927.11.8310	CITFA-4	1-1-1-0	nuclear enriched (mass spectrometry)	1Q1V_A	Winged Helix-related DNA binding domain	65.99
Q385A8	Tb927.11.6610	uncharacterized	1-1-1-1	-	2VOO_B<br>4WKR_A	La NTD RNA binding;<br>La RNA recognition Motif	99.96<br>99.85
Q387B3	Tb927.11.1390	CITFA-1	0-0-1-0	nuclear enriched (mass spectrometry)	NRM	NRM	NRM
Q38DS7	Tb927.9.10960	DEAD/H RNA helicase	other	nuclear enriched (mass spectrometry)	5SUP_B	DEAD-box RNA helicase	100
Q57XM9	Tb927.7.2450	uncharacterized	0-0-1-0	-	3NFQ_B<br>6PWV_A	Spn1/Iws1;<br>Histone binding	100<br>92.74

Enriched in YFP-HDAC3 affinity selections

UniProt ID	TriTryp ID	Gene name/description	[1]RIT-seq scoring matrix	Localization	PDB entry	HHpred domain	[2]HHpred domain probability %
Q387L4	Tb927.11.370	RAP1; also detected with CRD1 & TRF	0-0-1-0	nuclear enriched (mass spectrometry); telomeric (Nanavaty et al., 2017)	3AL2_A	BRCT domain	94.26
Q57UB5	Tb927.5.3510	SMC protein	other	nuclear enriched (mass spectrometry)	6WG3_B	SMC Cohesin/Condensin	100
Q580Q4	Tb927.4.5310	RDK2	other	-	6VPM_A	Serine/ThreonineProtein Kinase	100
Q388S9	Tb927.10.13180	NRAP	other	nuclear enriched (mass spectrometry)	4M5D_A	small nucleolar RNA-associated protein	100
Q4GZ82	Tb927.1.1370	rRNA biogenesis protein	other	nuclear enriched (mass spectrometry)	6LQU_RD	Ribosome assembly protein	100
Q580T1	Tb927.3.5440	SNF2 DNA repair protein	other	-	6IGM_H	SWI/SNF family chromatin remodeler	100
Q38DK9	Tb927.9.11850	SMC protein	other	nuclear enriched (mass spectrometry)	6WG3_A	SMC Cohesin/Condenin	100
Q385E2	Tb927.11.6270	PIP5Pase1; also detected with TRF	other	nuclear enriched (mass spectrometry)	1I9Z_A	Phosphatidyl-Inositol phosphate 5-phosphatase	99.89
Q385P5	Tb927.11.5230	uncharacterized; also detected with BDF3, BDF5, HAT2 & ZCW1	0-0-0-0	nuclear enriched (mass spectrometry); nuclear (D'Archivio and Wickstead, 2017)	1UZ3_A	EMSY ENT domain (Chromo shadow binding	95.93
Q38DH5	Tb927.9.12300	RFC	1-1-1-0	nuclear enriched (mass spectrometry)	6VVO_E	Replication factor C subunit 1	

SET26-ZCW1 network

UniProt ID	TriTryp ID	Gene name/description	[1]RIT-seq scoring matrix	Localization	PDB entry	HHpred domain	[2]HHpred domain probability %
Q38AF4	Tb927.10.8100	SET26	1-1-1-1	nuclear/cytoplasmic (this study)	3RQ4_A<br>1T6S_A	SET SUVAR20H2;<br>Winged-HTH	99.54<br>66.7
Q389G9	Tb927.10.11720	ZCW1;<br>also detected with BDF2	other	nuclear (this study); nuclear enriched (mass spectrometry)	6O5W_A	MORC family CW-type zinc finger	97.53
Q580R3	Tb927.3.5620	SPT16 (FACT)	0-0-0-0	nuclear enriched (mass spectrometry)	6UPL_G	Histone Chaperone FACT Complex	100
Q385D4	Tb927.11.6350	AAA ATPase; BDF7; similar to Yta7 (*S. cerevisiae*), ATAD2 (*H. sapiens*)	1-1-1-1	nuclear enriched (mass spectrometry)	6JQ0_C<br>6DNE_B	AAA+ ATPase Histone Chaperone;<br>Bromo (PFAM)	99.95<br>98.53
Q388G3	Tb927.10.14390	POB3 (FACT)	0-0-0-0	nuclear enriched (mass spectrometry)	6UPL_G	Histone Chaperone FACT Complex	100
Q583S0	Tb927.6.2640	Importin alpha;<br>also detected with TFIIS2-2, DOT1A, NUP110 & TRF	0-0-0-0	nuclear enriched (mass spectrometry)	4TNM_A	Importin Alpha 3	100
Q383E9	Tb927.11.11290	HSP70;<br>also detected with BDF3, BDF5, BDF7, TFIIS2-2 & DOT1A	1-1-1-0	nuclear enriched (mass spectrometry)	5FPN_A	Heast Shock 70 protein	100
Q386P3	Tb927.11.2670	NUP59	0-0-0-0	nuclear enriched (mass spectrometry)	5IJN_G	Nucleopore Complex (Nup155)	99
Q38D21	Tb927.9.14430	Casein Kinase II;<br>also detected with BDF4 & BDF7	other	-	5XVU_B	Caesin Kinase CKII	100
Q386Y4	Tb927.11.1700	uncharacterized	other	-	2GIA_D	RNA binding	97.54

Enriched in YFP-SET26 affinity selections

UniProt ID	TriTryp ID	Gene name/description	[1]RIT-seq scoring matrix	Localization	PDB entry	HHpred domain	[2]HHpred domain probability %
Q384B5	Tb927.11.9130	Replication factor A protein 1; possible SS telomeric DNA binding	0-0-0-0	-	6D6V_D<br>1JB7_A	Telomere SS terminal DNA 3′ extension binding protein	100<br>99.58
Q583H5	Tb927.4.3810	RPB2	0-0-0-0	nuclear enriched (mass spectrometry)	6GMH_B	RNAPII Rpb2	100
Q38D92	Tb927.9.13320	uncharacterized; also detected with BDF3, BDF5, & HAT2	other	nuclear enriched (mass spectrometry)	5XZW_A	FHA domain (binds phosphothreonine and phosphotyrosine peptides)	96.13
Q38AE0	Tb927.10.8240	Cytokinesis initiation factor 4	0-0-1-0	-	6JLB_D	Coiled-coil	96.19
Q580N2	Tb927.3.4100	Pyruvate transporter	other	-	6G9X_B	Major facilitator superfamily transporter	100
Q38A77	Tb927.10.8970	kinetoplast-associated protein 4 isoform 2	-	-	3TQ6_A	DNA binding (mitochondrial)	99.64
Q38E19	Tb927.9.9810	uncharacterized	other	nuclear enriched (mass spectrometry)	NRM	NRM	NRM
Q57YU2	Tb927.8.7400	RPB1; also detected with CRD1 & TFIIS2-2	0-0-0-0	nuclear enriched (mass spectrometry)	4XPZ_A	RNAPII Rpb1	96.21
Q385C3	Tb927.11.6460	uncharacterized	other	-	4P5X_A	Mitochondrial carrier protein	99.77
Q38FW0	Tb927.9.1980	uncharacterized	1-1-1-0	-	NRM	NRM	NRM
Q57WL5	Tb927.8.8150	C2 domain; also detected with BDF1	0-0-0-0	-	6FLJ_A	C2 domain (calcium binding, membrane bound)	99.2
Q57ZS7	Tb927.5.2080	GMP reductase; IMPDH	other	-	6RFU_A	Inosine-5'-monophosphate dehydrogenase	100
Q387Q3	Tb927.10.16120	IMPDH1; also detected with DOT1A & SET15	1-1-1-0	glycosomal (Guther et al., 2014)	6RFU_A	Inosine-5'-monophosphate dehydrogenase	100
Q388C7	Tb927.10.14770	Associated kinase of Tb14-3-3	1-1-1-0	-	6VPM_A	Serine/Threonine protein kinase	100
Q388K3	Tb927.10.13960	paralyzed flagella protein 20	0-0-1-0	flagellar (Branche et al., 2006; Ralston et al., 2006)	5L8E_B	WD40 repeat protein	100
Q389A2	Tb927.10.12410	uncharacterized; also detected with BDF1	other	-	1G0S_A	ADP-ribose pyrophosphatase	99.72
Q38DT1	Tb927.9.10920	kinetochore protein KKT3	0-0-1-0	nuclear enriched (mass spectrometry)	6VPM_A	Serine/Threonine protein kinase	100

Enriched in YFP-SET26 affinity selections - continued

UniProt ID	TriTryp ID	Gene name/description	[1]RIT-seq scoring matrix	Localization	PDB entry	HHpred domain	[2]HHpred domain probability %
Q584R4	Tb927.6.2420	p22 protein precursor	other	-	3JV1_A	P22, Mam33 family	100
Q386D5	Tb927.11.3770	Dpy-30 motif containing protein	0-0-1-0	-	4RT4_C<br>1XIQ_F	DPY-30 interaction domain (histone methyltransferase regulation);<br>Nucleoside diphosphate kinase	98.73<br>99.85
Q381T5	Tb927.11.15000	SMN-like	other	nuclear enriched (mass spectrometry)	5XJL_M	SMN-related snRNA assembly	87.4
Q38B98	Tb927.10.5030	ubiquitin/ribosomal protein S27a	0-0-1-0	-	5OPT_j	Kinetoplastid specific ribosomal protein (KSRP)	100
Q580S5	Tb927.3.5500	RPB3;<br>also detcted with CRD1 & TFIIS2-2	other	nuclear enriched (mass spectrometry)	1TWF_C	Rpb3 RNAPII subunit	100
Q582W2	Tb927.3.2490	Interacts with kinesin	1-1-0-0	-	1V8K_A<br>4QJ3_B<br>2ELJ_A	Kinesin-related;<br>Nucleotide-binding;<br>Transcriptional adapter	100<br>98.06<br>99.04
Q57X47	Tb927.5.4570	Flagellum adhesion protein 3	0-0-1-0	-	6GC1_C	NHL repeat β-propeller	99.21
Q57YE9	Tb927.8.820	VID27 cytoplasmic protein	other	nuclear enriched (mass spectrometry)	5ZWM_K<br>4J87_A	Splicing factor;<br>Vesicle trafficking	99.88<br>99.90

Enriched in YFP-ZCW1 affinity selections

UniProt ID	TriTryp ID	Gene name/description	[1]RIT-seq scoring matrix	Localization	PDB entry	HHpred domain	[2]HHpred domain probability %
Q57UN4	Tb927.7.4040	Similar to Swc4 (*S. cerevisiae*); also detected with BDF2	other	nuclear enriched (mass spectrometry)	4IEJ_A	DMAP1, chromatin regulator, SWR1 complex	97.52
Q383K6	Tb927.11.10730	SWI/SNF-related helicase; similar to Swr1 (*S. cerevisiae*); also detected with BDF2	other	nuclear enriched (mass spectrometry)	6FML_G	ATPase Helicase, SWR1/INO80 complexes	100
Q580C6	Tb927.4.980	actin-related; similar to Act1 (*S. cerevisiae*); also detected with BDF2	1-1-1-1	nuclear enriched (mass spectrometry)	4AM6_A	Actin or Actin-related protein, Chromatin remodelling	100
Q389H2	Tb927.10.11690	YEATS; similar to YEA1; Yaf9 (*S. cerevisiae*); also detected with BDF2	other	nuclear enriched (mass spectrometry)	6T1I_A	YEATS domain acetyl-/crotonyl-lysine binding	98.21
Q57ZL0	Tb927.3.3020	actin-related; similar to Arp6 (*S. cerevisiae*); also detected with BDF2	other	nuclear enriched (mass spectrometry)	4FO0_A	Actin-related protein, Chromatin remodelling	100
Q584P9	Tb927.6.2570	actin-related; also detected with BDF2	other	nuclear enriched (mass spectrometry)	4I6M_B	Actin-related protein, Chromatin remodelling	97.36
Q38C43	Tb927.10.2000	actin-related; similar to Arp4 (*S. cerevisiae*); also detected with BDF2	other	nuclear enriched (mass spectrometry)	6GEN_R	Actin-related protein, Chromatin remodelling	100
Q385E0	Tb927.11.6290	HIT zinc finger; similar to Swc6 (*S. cerevisiae*); also detected with BDF2	1-1-1-1	nuclear enriched (mass spectrometry)	2N95_A<br>6GEJ_S	HIT Zinc Finger; SWR1/INO80 complex linked to RuvB	97.12<br>99.4
Q57YH0	Tb927.8.600	Bucentaur/craniofacial development; also detected with BDF2	other	nuclear enriched (mass spectrometry)	6PX3_S	Histone/nucleosome binding (possible *S. cerevisiae* equivalent SWR1-C Swc5)	53.46
Q583J3	Tb927.4.2000	ruvB-like DNA helicase; similar to Rvb1/2 (*S. cerevisiae*); also detected with BDF2	0-0-1-0	-	6H7X_A	RuvB-like ATPase helicase, SWR1/INO80 complexes	99.96
Q385I5	Tb927.11.5830	Similar to Swc2 (*S. cerevisiae*)	0-0-0-0	-	5FUG_C	H2A.Z Chaperone, Nucleosome assembly, SWR1 complex	99.51
Q581V4	Tb927.4.1270	ruvB-like DNA helicase; similar to Rvb1/2 (*S. cerevisiae*); also detected with BDF2	0-0-1-0	-	2C9O_C	RuvB-like ATPase helicase, SWR1/INO80 complexes	100
Q387D1	Tb927.11.1210	DUF4470; DUF4471	1-1-1-0	-	5H02_A	Domain shared with methyltransferases	83.83
Q38AM1	Tb927.10.7420	BDF2; similar to Bdf1 (*S. cerevisiae*); also detected with HDAC3	1-1-1-1	-	6DNE_B<br>2ZD7_A	Bromo domain; NAP domain (histone chaperone)	99.29<br>83.7
Q385P5	Tb927.11.5230	uncharacterized; BDF3, BDF5, HAT2 & HDAC3	0-0-0-0	nuclear enriched (mass spectrometry); nuclear (D'Archivio and Wickstead, 2017)	1UZ3_A	EMSY ENT domain (Chromo shadow binding)	95.93

Enriched in YFP-ZCW1 affinity selections – continued

UniProt ID	TriTryp ID	Gene name/description	[1]RIT-seq scoring matrix	Localization	PDB entry	HHpred domain	[2]HHpred domain probability %
Q57XG4	Tb927.8.2250	tRNA ligase phosphodiesterase domain containing protein	other	-	6N67_A<br>6TZO_A	tRNA ligase;<br>nucleotide-binding	98.27<br>99.96
Q386Y7	Tb927.11.1670	Cysteine desulfurase; also detected with BDF7	other	nuclear (Kovarova et al., 2014; Smid et al., 2006)	4Q75_A	Cysteine Desulfurase	100
Q4GYK7	Tb927.1.3670	ESAG8 protein	-	-	3RGZ_A	Leucine Rich Repeat Receptor	100
Q382J7	Tb927.11.13400	BDF5; also detected with BDF3 & HAT2	0-0-1-0	nuclear (this study); nuclear enriched (mass spectrometry)	2R0Y_A<br>2F5J_B	Tandem Bromo Domains;<br>MRG Domain	99.7<br>96.87
Q387T9	Tb927.10.15750	TAC protein 197	0-0-0-0	basal bodies (Gheiratmand et al., 2013; Halliday et al., 2019)	6ZP4_A	40S Ribosomal protein	97.27
Q57ZX9	Tb927.5.3030	Intraflagellar transport protein 121	0-0-1-0	-	5N4A_A	Intraflagellar transport WD40, ZnF RBZ	100

BDF6-EAF6-HAT1 network

UniProt ID	TriTryp ID	Gene name/description	[1]RIT-seq scoring matrix	Localization	PDB entry	HHpred domain	[2]HHpred domain probability %
Q4GYN3	Tb927.1.3400	BDF6	other	nuclear (this study); nuclear enriched (mass spectrometry)	2R10_B	Bromo domain	99.14
Q38FN2	Tb927.9.2910	EAF6 NuA4 HAT subunit; similar to Eaf6 (*S. cerevisiae*); also detected with PHD1 & HAT3	1-1-1-1	nuclear (this study); nuclear enriched (mass spectrometry)	5J9T_B	Eaf6 subunit of NuA4 histone acetyltransferase complex	99.85
D6XIY7	Tb927.7.4560	HAT1	1-1-1-1	nuclear (this study)	7CMR_A	MYST Histone acetyltransferase	100
Q57ZF8	Tb927.7.5310	YEATS; similar to YEA2; Yaf9 (*S. cerevisiae*)	other	nuclear enriched (mass spectrometry)	5IQL_A	YEATS domain Acetyl-/Crotonyl-lysine histone binding	99.82
Q388I3	Tb927.10.14190	EPL1 NuA4 HAT subunit; similar to Epl1 (*S. cerevisiae*)	1-1-1-1	nuclear enriched (mass spectrometry)	5J9T_K	Epl1 subunit of NuA4 histone acetyltransferase complex	98.5
Q4GZG2	Tb927.1.650	EAF3 NuA4 HAT subunit; similar to Eaf3 (*S. cerevisiae*)	0-0-0-0	nuclear enriched (mass spectrometry)	2F5J_B	MRG domain protein; MRG15	97.87
Q57UK4	Tb927.8.5320	uncharacterized	1-1-1-1	-	2L9Z_A	Zinc binding	46.06
Q386G8	Tb927.11.3430	uncharacterized	other	-	NRM	NRM	NRM
Q585C3	Tb927.6.1240	uncharacterized	0-0-0-0	-	NRM	NRM	NRM

Enriched in YFP-BDF6 affinity selections

UniProt ID	TriTryp ID	Gene name/description	[1]RIT-seq scoring matrix	Localization	PDB entry	HHpred domain	[2]HHpred domain probability %
Q57UX1	Tb927.8.4810	Prohibitin1	other	-	4FVG_A	SPFH (Stomatin, Prohibitin, Flotillin, HflK/C) domain	99.71
Q57Z72	Tb927.5.3210	SUMO; also detected with BDF3, HAT2 & SET27	other	-	3PGE_A	SUMO (Small Ubiquitin-like protein)	99.03

Enriched in YFP-HAT1 affinity selections

UniProt ID	TriTryp ID	Gene name/description	[1]RIT-seq scoring matrix	Localization	PDB entry	HHpred domain	[2]HHpred domain probability %
Q383W5	Tb927.11.9640	glycyl-tRNA synthetase	0-0-1-0	-	2ZT5_A	Glycyl-tRNA synthetase	100
Q389D8	Tb927.10.12040	MAPK11	other	-	4IC7_A	Mitogen-activated protein kinase	100

Enriched in YFP-EAF6 affinity selections

UniProt ID	TriTryp ID	Gene name/description	[1]RIT-seq scoring matrix	Localization	PDB entry	HHpred domain	[2]HHpred domain probability %
Q389Y3	Tb927.10.9930	PHD1	1-1-1-1	nuclear (this study)	4NN2_A<br>5J9T_K	PHD Zinc Finger domain (Nto1/NuA3);<br>Region similar to Epl1	99.45<br>97.0
Q384N0	Tb927.11.7880	ING/NUA4 HAT; similar to Yng2 (*S. cerevisiae*); also detected with PHD1 & HAT3	0-0-1-0	-	5J9T_D	ING family (methyl-lysine histone), Esa1 NuA4 HAT complex	96.76
Q38AD3	Tb927.10.8310	HAT3	0-0-0-0	nuclear (this study)	3TO7_A	Esa1 MYST histone acetyltransferase	100
Q38C15	Tb927.10.2290	DnaJ3	1-1-0-0	-	3LZ8_B	DNAJ Chaperone	100
Q57V04	Tb927.7.2070	DNAJ	1-1-1-0	nuclear enriched (mass spectrometry)	4J80_C	DNAJ Chaperone / Heat Shock protein 40	99.9
Q38DC5	Tb927.9.12900	LEO1;<br>RNAPII associated;<br>also detected with TFIIS2-2	0-0-0-0	-	4M6T_A	Leo1 domain, Paf1 Complex - RNAPII transcription elongation regulation	99.87
Q586X9	Tb927.2.3580	TFIIS2-1;<br>also detected with CRD1, SET27 & TFIIS2-2	1-1-1-1	nuclear enriched (mass spectrometry)	1PQV_S<br>2M1H_A	TFIIS;<br>PWWP domain	100<br>99.22
D6XHJ3	Tb927.6.1090	proteasome regulatory ATPase subunit 3	0-0-1-0	-	6FVT_I	26S Proteasome subunit	99.96
Q383D0	Tb927.11.11480	Trichohyalin (involved in stumpy formation)	other	-	NRM	NRM	NRM
Q57Y42	Tb927.8.7980	Pyrophosphate-energized vacuolar membrane proton pump 2	1-1-1-1	-	6AFW_B	Vacuolar membrane proton pump	100

Enriched in YFP-HDAC1 affinity selections

UniProt ID	TriTryp ID	Gene name/description	[1]RIT-seq scoring matrix	Localization	PDB entry	HHpred domain	[2]HHpred domain probability %
Q38C74	Tb927.10.1680	HDAC1	other	nuclear/cytoplasmic (this study); nuclear enriched (mass spectrometry); nuclear in BF, nuclear/cytoplasmic in PF (Wang et al., 2010)	4A69_A	Histone Deacetylase	100
Q57WX2	Tb927.3.890	uncharacterized	1-1-1-1	-	5JXT_L	ISWI Histone H4 tail bound	35.78
Q585H7	Tb927.4.3730	uncharacterized	other	-	6R3E_B	PWWP Histone binding	45.72
Q584W0	Tb927.6.3170	uncharacterized	1-1-1-1	nuclear enriched (mass spectrometry)	6IGM_H	RuvB-like, SWR/INO80 Complexes	66.2
Q57TZ5	Tb927.7.1650	uncharacterized	1-1-1-1	nuclear enriched (mass spectrometry)	NRM	NRM	NRM
Q38FV1	Tb927.9.2070	uncharacterized; also detected with CRD1	other	nuclear enriched (mass spectrometry)	NRM	NRM	NRM

Enriched in YFP-BDF7 affinity selections (top interactors)

UniProt ID	TriTryp ID	Gene name/description	[1]RIT-seq scoring matrix	Localization	PDB entry	HHpred domain	[2]HHpred domain probability %
Q385D4	Tb927.11.6350	AAA ATPase; BDF7; similar to Yta7 (*S. cerevisiae*), ATAD2 (*H. sapiens*); also detected with SET26 & ZCW1	1-1-1-1	nuclear (this study); nuclear enriched (mass spectrometry)	6JQ0_C<br>6DNE_B	AAA+ ATPase Histone Chaperone;<br>Bromo (PFAM)	99.95<br>98.53
Q4GZ00	Tb927.1.2210	NAP1; nucleosome assembly protein	other	-	2AYU_A	Nucleosome Assembly Protein 1 histone chaperone	100
Q582P7	Tb927.3.4880	NAP2; nucleosome assembly protein	1-1-1-1	-	5DAY_B	Nucleosome Assembly Protein 1-related histone chaperone	100
Q38D21	Tb927.9.14430	Casein kinase II; also detected with BDF4, SET26 & ZCW1	other	-	5XVU_B	Casein Kinase CKII	100
Q383J7	Tb927.11.10820	Casein kinase II subunit beta; also detected with BDF4	other	-	4DGL_A	Casein Kinase II subunit beta	100
Q386Y7	Tb927.11.1670	cysteine desulfurase; also detected with ZCW1	other	nuclear (Kovarova et al., 2014; Smid et al., 2006)	4Q75_A	Cysteine Desulfurase	100
Q585P0	Tb927.6.1710	uncharacterized	other	-	NRM	NRM	NRM
Q385C7	Tb927.11.6420	uncharacterized	other	nuclear enriched (mass spectrometry)	5HDT_B	Cohesin regulator PDS5	100
Q388S2	Tb927.10.13250	uncharacterized	1-1-1-1	-	4J0W_A	Beta-Propeller, WD domain	95.19
Q388P7	Tb927.10.13510	zinc metallopeptidase	1-1-1-1	-	6NYY_F	AAA+, ATPase, protease, mitochondria	100
Q381W2	Tb927.11.14730	Metalloprotease M41 FtsH	1-1-1-0	-	6NYY_F	AAA+, ATPase, protease, mitochondria	100
Q389E2	Tb927.10.12000	iron-sulfur cluster assembly protein	other	-	6UXE_B	Mitochondrial Cysteine desulfurase	99.65
Q38DH1	Tb927.9.12340	uncharacterized	0-0-1-0	nuclear enriched (mass spectrometry)	4P5X_A	Calcium binding protein EF-Hand	99.82
Q387Z3	Tb927.10.15180	NAP3; nucleosome assembly protein	other	-	2ZD7_A	NAP family histone Chaperone (Nap1/Vps75 S. cerevisiae)	100
Q383E9	Tb927.11.11290	HSP70; also detected with BDF3, BDF5, SET26, ZCW1, TFIIS2-2 & DOT1A	1-1-1-0	nuclear enriched (mass spectrometry)	5FPN_A	Heast Shock 70 protein	100
Q38BN5	Tb927.10.3640	nuclear transmembrane protein	1-1-1-0	nuclear enriched (mass spectrometry)	NRM	NRM	NRM

Enriched in YFP-BDF7 affinity selections (top interactors) - continued

UniProt ID	TriTryp ID	Gene name/description	[1]RIT-seq scoring matrix	Localization	PDB entry	HHpred domain	[2]HHpred domain probability %
Q57WG0	Tb927.7.3450	protein I/6	-	-	3MWU_A	Calcium binding protein EF-Hand	99.59
Q38BJ3	Tb927.10.4070	uncharacterized	other	nuclear enriched (mass spectrometry)	2KMU_A	Possible ATP dependent helicase	98.03

Enriched in YFP-TFIIS2-2 affinity selections (top interactors)

UniProt ID	TriTryp ID	Gene name/description	[1]RIT-seq scoring matrix	Localization	PDB entry	HHpred domain	[2]HHpred domain probability %
Q586Y0	Tb927.2.3480	TFIIS2-2	other	nuclear (this study); nuclear enriched (mass spectrometry)	3PMI_C<br>1PQV_S	PWWP domain;<br>TFIIS	99.04;<br>98.45
Q582N0	Tb927.3.5070	uncharacterized	0-0-1-0	nuclear enriched (mass spectrometry)	NRM	NRM	NRM
Q38DC5	Tb927.9.12900	LEO1; RNAPII associated; also detected with EAF6	0-0-0-0	-	4M6T_A	Leo1 domain, Paf1 Complex - RNAPII transcription elongation regulation	99.87
Q57V64	Tb927.3.3220	CTR9; RNAPII associated	0-0-0-0	nuclear enriched (mass spectrometry)	6AF0_A	Ctr9 / TPR<br>Paf1 Complex - RNAPII transcription elongation regulation	100
Q586X9	Tb927.2.3580	TFIIS2-1;<br>also detected with CRD1, EAF6 & SET27	1-1-1-1	nuclear enriched (mass spectrometry)	1PQV_S<br>2M1H_A	TFIIS;<br>PWWP domain	100<br>99.22
Q57UN3	Tb927.7.4030	uncharacterized	0-0-1-0	nuclear enriched (mass spectrometry)	4L1P_A	RTF1 subunit PAF Complex (RNAPII associated)	96.9
Q586C8	Tb927.2.5810	SPT6; similar to Spt6 (*S. cerevisiae*)	0-0-0-0	nuclear enriched (mass spectrometry)	3PSI_A	Transcription elongation factor SPT6	100
Q583H5	Tb927.4.3810	RPB2;<br>also detected with SET26	0-0-0-0	nuclear enriched (mass spectrometry)	6GMH_B	RNAPII Rpb2	100
Q57YU2	Tb927.8.7400	RPB1;<br>also detected with CRD1 & SET26	0-0-0-0	nuclear enriched (mass spectrometry)	4XPZ_A	RNAPII Rpb1	96.21
Q57Z69	Tb927.5.3240	uncharacterized; possible Rpb1-associated protein	0-0-1-0	nuclear enriched (mass spectrometry)	3LLR_B<br>2C5Z_A<br>5IJO_N	PWWP domain;<br>Set2 Rpb1-interacting (SRI) domain;<br>Nuclear Pore Complex	83.8<br>53.92<br>78.05
Q38BC1	Tb927.10.4800	JBP3	1-1-1-1	-	2XSE_A	Thymine Dioxygenase	98.34
Q580S5	Tb927.3.5500	RPB3;<br>also detcted with CRD1 & SET26		nuclear enriched (mass spectrometry)	1TWF_C	Rpb3 RNAPII subunit	100
Q383S2	Tb927.11.10070	BDF3		nuclear enriched (mass spectrometry)	4QB3_A<br>2NCZ_A	Bromo domain;<br>ET Domain	99.11<br>98.72
Q389E6	Tb927.10.11960	PNUTS;<br>also detected with SET27		-	NRM	NRM	NRM

Enriched in YFP-DOT1A affinity selections

UniProt ID	TriTryp ID	Gene name/description	[1]RIT-seq scoring matrix	Localization	PDB entry	HHpred domain	[2]HHpred domain probability %
Q4GYF6	Tb927.1.4220	RNase H2B	1-1-1-1	nuclear enriched (mass spectrometry)	3KIO_B	Ribonuclease H2 subunit B	100
Q581Z0	Tb927.8.1920	DOT1A	1-1-1-1	nuclear (this study)	1U2Z_B	Histone lysine N-methyltransferase (H3K79-specific)	99.75
Q38B94	Tb927.10.5070	RNase H2A	other	-	3P5J_A	Ribonuclease H2 subunit A	100
Q38F34	Tb927.9.5190	proliferating cell nuclear antigen (PCNA)	0-0-0-0	-	5H0T_A	Proliferating cell nuclear antigen	100
Q4GYA5	Tb927.1.4730	RNase H2C	other	-	3PUF_L	Ribonuclease H2 subunit C	99.97
Q583S0	Tb927.6.2640	importin alpha subunit;<br>also detected with SET26, ZCW1, TFIIS2-2, NUP110 & TRF	0-0-0-0	nuclear enriched (mass spectrometry)	4TNM_A	Importin Alpha 3	100
Q387Q3	Tb927.10.16120	IMPDH1;<br>also detected with SET26 & SET15	1-1-1-0	glycosomal (Guther et al., 2014)	6RFU_A	Inosine-5'-monophosphate dehydrogenase	100
Q383E9	Tb927.11.11290	HSP70;<br>also detected with BDF3, BDF5, BDF7, SET26, ZCW1 & TFIIS2-2	1-1-1-0	nuclear enriched (mass spectrometry)	2FE0_A	Heat Shock 70 protein	100

Enriched in YFP-TRF affinity selections (top interactors)

UniProt ID	TriTryp ID	Gene name/description	[1]RIT-seq scoring matrix	Localization	PDB entry	HHpred domain	[2]HHpred domain probability %
Q388W1	Tb927.10.12850	TRF; telomere-repeat bining protein; also detected with BDF2 & HDAC3	0-0-0-0	nuclear (this study); nuclear enriched (mass spectrometry); telomeric (Jehi et al., 2014; Li et al., 2005)	1GV2_A	MYB domain DNA binding	80.11
Q586Z9	Tb927.6.4330	telomere-associated protein; also detected with BDF2 & HDAC3	0-0-0-0	nuclear enriched (mass spectrometry), telomeric	NRM	NRM	NRM
Q38FD7	Tb927.9.4000	uncharacterized; also detected with BDF2 & HDAC3	-	nuclear enriched (mass spectrometry), telomeric	NRM	NRM	NRM
Q57XY9	Tb927.3.1560	TIF2; also detected with BDF2 & HDAC3	other	nuclear enriched (mass spectrometry), telomeric (Jehi et al., 2014)	NRM	NRM	NRM
Q383U2	Tb927.11.9870	TelAP1; also detected with BDF2 & HDAC3	1-1-1-1	nuclear enriched (mass spectrometry), telomeric	4B0Z_A	Proteasome subunit	56.61
Q385L3	Tb927.11.5550	DNA polymerase theta (POLQ); also detected with BDF2 & HDAC3	1-1-1-1	nuclear enriched (mass spectrometry), telomeric	4X0Q_B	DNA polymerase theta	100
Q38BZ3	Tb927.10.2520	PrimPol-like protein 2; also detected with BDF2 & HDAC3	-	nuclear enriched (mass spectrometry)	5L2X_B	DNA Primase Polymerase	100
Q387L4	Tb927.11.370	RAP1; also detected with CRD1 & HDAC3	0-0-1-0	nuclear enriched (mass spectrometry); telomeric (Nanavaty et al., 2017)	3AL2_A	BRCT domain	94.26
Q586J9	Tb927.2.2190	HDAC3; also detected with BDF2	0-0-0-0	nuclear (this study); nuclear in BF and PF (Wang et al., 2010)	2VQM_A	HDAC Histone Deacetylase	100
Q385E2	Tb927.11.6270	PIP5Pase1; also detcted with HDAC3	-	nuclear enriched (mass spectrometry)	1I9Z_A	Phosphatidyl-Inositol phosphate 5-phosphatase	99.89

Enriched in YFP-TBP affinity selections

UniProt ID	TriTryp ID	Gene name/description	[1]RIT-seq scoring matrix	Localization	PDB entry	HHpred domain	[2]HHpred domain probability %
Q387R9	Tb927.10.15950	*TBP/TRF4	other	nuclear/cytoplasmic (this study); nuclear enriched (mass spectrometry)	1RM1_A	TATA-box binding protein	100
Q38E05	Tb927.9.9970	*SNAP50	1-1-1-1	nuclear enriched (mass spectrometry)	5N7Y_A	Zn Finger	47.94
Q599N4	Tb927.10.15570	*TFIIA-1	other	-	NRM	NRM	NRM
Q599N5	Tb927.10.7070	*SNAP3	other	nuclear enriched (mass spectrometry)	NRM	NRM	NRM
Q599N6	Tb927.5.3910	*SNAP2	1-1-0-0	nuclear enriched (mass spectrometry)	1FEX_A	Myb HTH DNA binding domain	88.16
Q387K4	Tb927.11.470	BRF1 (TFIIB)	-	-	6F40_V<br>4BBR_M	TFIIIB;<br>TFIIB	100<br>99.98
Q599N3	Tb927.10.4840	*TFIIA-2	1-1-1-1	-	5M4S_A	TFIIA	99.94
Q38DC3	Tb927.9.12950	uncharacterized	other	-	NRM	NRM	NRM
Q384L9	Tb927.11.8000	uncharacterized	other	-			

*TRF4/SNAPc/TFIIA Complex (Das et al., 2005; Schimanski et al., 2005)

Enriched in YFP-ELP3b affinity selections

UniProt ID	TriTryp ID	Gene name/description	[1]RIT-seq scoring matrix	Localization	PDB entry	HHpred domain	[2]HHpred domain probability %
Q57YY2	Tb927.8.3310	ELP3b	other	nuclear/cytoplasmic (this study); nuclear enriched (mass spectrometry)	6IA8_A	Elp3 tRNA acetyltransferase	100
Q585H2	Tb927.2.1210	retrotransposon hot spot protein 4 (RHS4)	-	-	NRM	NRM	NRM
Q57ZH2	Tb927.7.5170	60S ribosomal protein L25	0-0-0-0	-	4V8M_BX	Ribosome component	100
Q583F5	Tb927.4.4700	uncharacterized	1-1-1-1	-	5IJO_N	Nuclear pore	90.41
Q387A9	Tb927.11.1430	Component of motile flagella 2	1-1-1-1	flagellar (Baron et al., 2007)	6U42_6W	Tubulin related	100

Enriched in YFP-PHD2 affinity selections

UniProt ID	TriTryp ID	Gene name/description	[1]RIT-seq scoring matrix	Localization	PDB entry	HHpred domain	[2]HHpred domain probability %
Q387D3	Tb11.v5.0388	PHD2	other	nuclear (this study)	6GLC_A	RBR-type ubiquitin ligase (Parkin)	97.46

Enriched in YFP-PHD4 affinity selections

UniProt ID	TriTryp ID	Gene name/description	[1]RIT-seq scoring matrix	Localization	PDB entry	HHpred domain	[2]HHpred domain probability %
Q57Z97	Tb927.3.2140	PHD4	1-1-1-0	nuclear (this study)	3MWY_W<br>5XFO_A	Swi2/Snf2 ATPase remodeller (Chd1);<br>Tandem PHD Fingers	100<br>97.54
Q587E7	Tb927.2.3370	UDP-Gal or UDP-GlcNAc-dependent glycosyltransferase	other	-	2J0A_A	Beta1,3-N-acetyl-glucosaminyl-transferase	99.87

Enriched in YFP-PHD1 affinity selections

UniProt ID	TriTryp ID	Gene name/description	[1]RIT-seq scoring matrix	Localization	PDB entry	HHpred domain	[2]HHpred domain probability %
Q389Y3	Tb927.10.9930	PHD1;<br>also detected with EAF6 & HAT3	1-1-1-1	nuclear (this study)	4NN2_A<br>5J9T_K	PHD Zinc Finger domain (Nto1/NuA3);<br>Region similar to Epl1	99.45<br>97.00
Q384N0	Tb927.11.7880	ING/NUA4 HAT; similar to Yng2 (*S. cerevisiae*);<br>also detected with EAF6 & HAT3	0-0-1-0	-	5J9T_D	ING family (methyl-lysine histone),<br>Esa1 NuA4 HAT complex	96.76
Q38FN2	Tb927.9.2910	EAF6 NUA4 HAT subunit; similar to Eaf6 (*S. cerevisiae*);<br>also detected with BDF6, HAT1 & HAT3	1-1-1-1	nuclear (this study); nuclear enriched (mass spectrometry)	5J9T_B	Eaf6 subunit of NuA4 histone acetyltransferase complex	99.85
Q38AD3	Tb927.10.8310	HAT3;<br>also detected with EAF6	0-0-0-0	nuclear (this study)	3TO7_A	Esa1 MYST histone acetyltransferase	100

Enriched in YFP-PHD5 affinity selections

UniProt ID	TriTryp ID	Gene name/description	[1]RIT-seq scoring matrix	Localization	PDB entry	HHpred domain	[2]HHpred domain probability %
Q385I1	Tb927.11.5870	PHD5	1-1-1-1	nuclear/cytoplasmic (this study)	3TT2_A<br>3KQI_A	GCN5-related N-acetyltransferase;<br>PHD finger	99.03<br>90.64

Enriched in YFP-HAT3 affinity selections

UniProt ID	TriTryp ID	Gene name/description	[1]RIT-seq scoring matrix	Localization	PDB entry	HHpred domain	[2]HHpred domain probability %
Q38AD3	Tb927.10.8310	HAT3; also detected with EAF6 and PHD1	0-0-0-0	nuclear (this study)	3TO7_A	Esa1 MYST histone acetyltransferase	100
Q389Y3	Tb927.10.9930	PHD1; also detected with EAF6	1-1-1-1	nuclear (this study)	4NN2_A<br>5J9T_K	PHD Zinc Finger domain (Nto1/NuA3);<br>Region similar to Epl1	99.45<br>97.0
Q384N0	Tb927.11.7880	ING/NUA4 HAT; similar to Yng2 (*S. cerevisiae*); also detected with EAF6 and PHD1	0-0-1-0	-	5J9T_D	ING family (methyl-lysine histone)<br>Esa1 NuA4 HAT complex	96.76
Q38FN2	Tb927.9.2910	EAF6 NUA4 HAT subunit; similar to Eaf6 (*S. cerevisiae*); also detected with BDF6, HAT1, & PHD1	1-1-1-1	nuclear (this study) nuclear enriched (mass spectrometry)	5J9T_B	Eaf6 subunit of NuA4 histone acetyltransferase complex	99.85
Q57YG1	Tb927.8.690	PIN1	1-1-1-1	-	2LJ4_A	Peptidyl-prolyl cis-trans isomerase	99.68
B3GVR6	Tb427.BES126.2	Transferrin-binding	-	-	6SOY_B	ESAG6, subunit of heterodimeric transferrin	100

Enriched in YFP-AGO1 affinity selections

UniProt ID	TriTryp ID	Gene name/description	[1]RIT-seq scoring matrix	Localization	PDB entry	HHpred domain	[2]HHpred domain probability %
Q389P5	Tb927.10.10850	AGO1	1-1-1-1	cytoplasmic (this study)	4Z4D_A	Argonaute protein	100
Q38A86	Tb927.10.8880	RIF4	1-1-1-1	-	4YBG_A	Ribonuclease, Maelstrom, silencing	99.95
Q389B0	Tb927.10.12330	ZC3H34	1-1-0-0	-	6EOJ_B<br>5GUH_A	3' end processing Zinc finger;<br>Endonuclease	92.87<br>65.86
Q38E63	Tb927.9.9290	PABP1	0-0-0-0	cytoplasmic (Kramer et al., 2013)	6R5K_H	Polyadenylate-binding protein (Pab1)	100
Q585M4	Tb927.6.1870	EIF4E4	1-1-1-0	cytoplasmic (Kramer et al., 2013)	5T46_A	Eukaryotic translation initiation factor 4E	100

Enriched in YFP-NUP110 affinity selections

UniProt ID	TriTryp ID	Gene name/description	[1]RIT-seq scoring matrix	Localization	PDB entry	HHpred domain	[2]HHpred domain probability %
Q387L8	Tb927.11.330	NUP110 (MLP1)	0-0-1-0	nuclear pores/ cytoplasmic (this study); nuclear enriched (mass spectrometry)	6FSA_A	Myosin light chain	95.16
		24 nucleoporins (see Supplemental Table S4)					

Frequent non-specific contaminants

UniProt ID	TriTryp ID	Gene name/description	[1]RIT-seq scoring matrix	Localization	PDB entry	HHpred domain	[2]HHpred domain probability %
Q586Q9	Tb927.2.4580	UNC119	1-1-1-1	lipidated protein intraflagellar transport (Fritz et al., 2015; Ohshima et al., 2010; Pandey et al., 2020)	3GQQ_E	human retinal protein 4 (unc-119)	100

Supplemental Table S6

Supplemental Table S6. *T. brucei* homologs of SWR1-C/SRCAP-C/EP400 subunits

S. cerevisiae SWR1-C	*H. sapiens* SRCAP-C or EP400	*T. brucei* SWR1-C	Description
Swr1	SRCAP	Tb927.11.10730	Swi2/Snf2-related ATPase motor
Rvb1	RuvBL1	Tb927.4.2000/ Tb927.4.1270	ATP-dependent DNA helicase
Rvb2	RuvBL2	Tb927.4.2000/ Tb927.4.1270	ATP-dependent DNA helicase
Actin (Act1)	Actl6a/Arp4	Tb927.4.980	Actin
Arp4	Cfdp1/Swc5	Tb927.10.2000	Actin-related
Arp6	Arp6/Actr6	Tb927.3.3020	Actin-related
Swc2	YL1	Tb927.11.5830	H2A.Z Chaperone
Swc3	-	-	
Swc4	DMAP1	Tb927.7.4040	
Swc6	ZNHIT1	Tb927.11. 6290	
Swc7	-	-	
Yaf9	Gas41	Tb927.10.11690 (YEA1)	YEATS domain; binds acetyl/crotonyl-lysine
Bdf1	BRD8	Tb927.10.7420 (BDF2)	Bromodomain protein

Supplemental Table S7

Supplemental Table S7. *T. brucei* homologs of NuA4 subunits

S. cerevisiae **NuA4 Complex**	***H. sapiens*** **NuA4**	***T. brucei*** **NuA4 Complex**	**Description**
Eaf1	[EP400]	-	
-	BRD8	Tb927.1.3400 (BDF6)	Bromodomain
Epl1	EPC1/EPC-like	Tb927.10.14190	EPL1 domain
Esa1	TIP60	Tb927.7.4560 (HAT1)<br>Tb927.10.8310 (HAT3)	MYST Histone acetyltransferase
Eaf3	MRG15	Tb927.1.650	MRG domain
Eaf6	EAF6	Tb927.9.2910 (EAF6)	NuA4 domain
Yng2	ING3	Tb927.11.7880 (YNG2)	ING family
Yaf9	GAS41	Tb927.7.5310 (YEA2)	YEATS domain binds acetyl/crotonyl-lysine

Supplemental Table S8

Supplemental Table S8. Overlap of tRNA and snRNA genes annotated in the assembled Lister 427 genome with TTRs and RNAPIII/TTR-associated factors

Chromosome	Start position	End position	tRNA/ snRNA	ID	Overlap with TTR regions	Overlap with TTR factors
Chr2_core	742379	742527	snRNA	Tb427v9_000695200:snRNA (U2)	sTTR_56	yes
Chr3_core	1100184	1100266	tRNA	Tb427v9_000761300:tRNA;aa=Ser;anticodon=aga	sTTR_55	yes
Chr3_core	1100470	1100552	tRNA	Tb427v9_000761400:tRNA;aa=Leu;anticodon=caa	sTTR_55	yes
Chr3_core	1100611	1100693	tRNA	Tb427v9_000761500:tRNA;aa=Ser;anticodon=cga	sTTR_55	yes
Chr4_core	238353	238424	tRNA	Tb427v9_000825700:tRNA;aa=Pro;anticodon=cgg	no	only TBP
Chr4_core	246708	246806	snRNA	Tb427v9_000826000:snRNA (U6)	cTTR_69	yes
Chr4_core	246907	246978	tRNA	Tb427v9_000826100:tRNA;aa=Thr;anticodon=cgt	cTTR_69	yes
Chr4_core	247045	247129	tRNA	Tb427v9_000826200:tRNA;aa=Tyr;anticodon=gta	cTTR_69	yes
Chr4_core	767118	767191	tRNA	Tb427v9_000846700:tRNA;aa=Val;anticodon=aac	no	yes
Chr4_core	767254	767335	tRNA	Tb427v9_000846800:tRNA;aa=Leu;anticodon=tag	no	yes
Chr4_core	767393	767466	tRNA	Tb427v9_000846900:tRNA;aa=Met;anticodon=cat	no	yes
Chr4_core	780197	780268	tRNA	Tb427v9_000847600:tRNA;aa=Glu;anticodon=ctc	cTTR_70	yes
Chr4_core	780356	780429	tRNA	Tb427v9_000847700:tRNA;aa=Met;anticodon=cat	cTTR_70	yes
Chr4_core	780471	780553	tRNA	Tb427v9_000847800:tRNA;aa=Ser;anticodon=tga	cTTR_70	yes
Chr4_core	780669	780742	tRNA	Tb427v9_000847900:tRNA;aa=Val;anticodon=aac	cTTR_70	yes
Chr4_core	1212837	1212909	tRNA	Tb427v9_000862100:tRNA;aa=Ser;anticodon=cga	sTTR_52	yes
Chr4_core	1212978	1213053	tRNA	Tb427v9_000862200:tRNA;aa=Val;anticodon=cac	sTTR_52	yes
Chr5_core	147572	147644	tRNA	Tb427v9_000901600:tRNA;aa=Arg;anticodon=acg	no	only PHD2 and TBP
Chr7_core	284886	284957	tRNA	Tb427v9_001104600:tRNA;aa=Gly;anticodon=gcc	no	only PHD2 and TBP
Chr7_core	1885443	1885516	tRNA	Tb427v9_001164700:tRNA;aa=Ala;anticodon=agc	no	yes
Chr7_core	1885585	1885667	tRNA	Tb427v9_001164800:tRNA;aa=Ser;anticodon=gct	no	yes
Chr7_core	1885743	1885815	tRNA	Tb427v9_001164900:tRNA;aa=Asp;anticodon=gtc	no	yes
Chr7_core	1888962	1889034	tRNA	Tb427v9_001165000:tRNA;aa=Asp;anticodon=gtc	sTTR_41	yes
Chr7_core	1889110	1889192	tRNA	Tb427v9_001165100:tRNA;aa=Ser;anticodon=gct	sTTR_41	yes
Chr7_core	1889261	1889334	tRNA	Tb427v9_001165200:tRNA;aa=Ala;anticodon=agc	sTTR_41	yes
Chr7_core	1905849	1905923	tRNA	Tb427v9_001165500:tRNA;aa=Lys;anticodon=ctt	cTTR_48	all except DOT1A and TRF
Chr7_core	2057285	2057357	tRNA	Tb427v9_001169700:tRNA;aa=Ala;anticodon=cgc	no	no

Chromosome	Start position	End position	tRNA/ snRNA	ID	Overlap with TTR regions	Overlap with TTR factors
Chr8_core	657296	657369	tRNA	Tb427v9_001263400:tRNA;aa=Asn;anticodon=gtt	sTTR_30	yes
Chr8_core	657453	657526	tRNA	Tb427v9_001263500:tRNA;aa=Trp;anticodon=cca	sTTR_30	yes
Chr8_core	717533	717608	tRNA	Tb427v9_001265400:tRNA;aa=Ile;anticodon=tat	cTTR_34	yes
Chr8_core	717702	717773	tRNA	Tb427v9_001265500:tRNA;aa=Gln;anticodon=ttg	cTTR_34	yes
Chr8_core	717830	717905	tRNA	Tb427v9_001265600:tRNA;aa=Val;anticodon=tac	cTTR_34	yes
Chr8_core	717974	718047	tRNA	Tb427v9_001265700:tRNA;aa=Lys;anticodon=ctt	cTTR_34	yes
Chr8_core	721366	721438	tRNA	Tb427v9_001265800:tRNA;aa=Gly;anticodon=tcc	cTTR_34	yes
Chr8_core	721524	721607	tRNA	Tb427v9_001265900:tRNA;aa=Leu;anticodon=cag	cTTR_34	yes
Chr8_core	721690	721761	tRNA	Tb427v9_001266000:tRNA;aa=Thr;anticodon=tgt	cTTR_34	yes
Chr8_core	721859	721931	tRNA	Tb427v9_001266100:tRNA;aa=Arg;anticodon=tcg	cTTR_34	yes
Chr8_core	724624	724697	tRNA	Tb427v9_001266200:tRNA;aa=Lys;anticodon=ctt	cTTR_34	yes
Chr8_core	724788	724861	tRNA	Tb427v9_001266300:tRNA;aa=Arg;anticodon=acg	cTTR_34	yes
Chr8_core	916520	916591	tRNA	Tb427v9_001273200:tRNA;aa=Cys;anticodon=gca	cTTR_35	all except TFIIS2-2
Chr8_core	916732	916803	tRNA	Tb427v9_001273300:tRNA;aa=Cys;anticodon=gca	cTTR_35	all except TFIIS2-2
Chr8_core	1474462	1474534	tRNA	Tb427v9_001293300:tRNA;aa=Arg;anticodon=acg	cTTR_36	yes
Chr8_core	1777934	1778008	tRNA	Tb427v9_001305900:tRNA;aa=Ile;anticodon=aat	cTTR_37	yes
Chr8_core	1778055	1778139	tRNA	Tb427v9_001306000:tRNA;aa=Leu;anticodon=taa	cTTR_37	yes
Chr8_core	1778232	1778304	tRNA	Tb427v9_001306100:tRNA;aa=Gln;anticodon=ctg	cTTR_37	yes
Chr8_core	1778390	1778463	tRNA	Tb427v9_001306200:tRNA;aa=Gln;anticodon=ctg	cTTR_37	yes
Chr8_core	1778542	1778616	tRNA	Tb427v9_001306300:tRNA;aa=Ile;anticodon=aat	cTTR_37	yes
Chr9_core	236288	236373	tRNA	Tb427v9_001409300:tRNA;aa=seC;anticodon=tca	no	no
Chr9_core	240125	240210	tRNA	Tb427v9_001409500:tRNA;aa=seC;anticodon=tca	no	no
Chr9_core	1795249	1795319	tRNA	Tb427v9_001488600:tRNA;aa=Gly;anticodon=gcc	cTTR_25	all except BDF7 and DOT1A
Chr10_core	1888402	1888472	tRNA	Tb427v9_000188600:tRNA;aa=Gly;anticodon=gcc	sTTR_13	yes
Chr10_core	1924887	1924958	tRNA	Tb427v9_000190100:tRNA;aa=Pro;anticodon=agg	sTTR_14	all except TFIIS2-2
Chr10_core	1925019	1925091	tRNA	Tb427v9_000190200:tRNA;aa=Thr;anticodon=agt	sTTR_14	all except TFIIS2-2
Chr10_core	1928536	1928620	tRNA	Tb427v9_000190400:tRNA;aa=Leu;anticodon=aag	sTTR_14	yes
Chr10_core	1928681	1928752	tRNA	Tb427v9_000190500:tRNA;aa=Met;anticodon=cat	sTTR_14	yes
Chr10_core	1928825	1928896	tRNA	Tb427v9_000190600:tRNA;aa=Pro;anticodon=tgg	sTTR_14	yes

Chromosome	Start position	End position	tRNA/ snRNA	ID	Overlap with TTR regions	Overlap with TTR factors
Chr10_core	2645688	2645762	tRNA	Tb427v9_000223600:tRNA;aa=Asn;anticodon=gtt	sTTR_16	yes
Chr10_core	2645841	2645913	tRNA	Tb427v9_000223700:tRNA;aa=Ala;anticodon=tgc	sTTR_16	yes
Chr10_core	2646016	2646089	tRNA	Tb427v9_000223800:tRNA;aa=Arg;anticodon=tct	sTTR_16	yes
Chr10_core	2646149	2646221	tRNA	Tb427v9_000223900:tRNA;aa=Lys;anticodon=ttt	sTTR_16	yes
Chr10_core	2646305	2646379	tRNA	Tb427v9_000224000:tRNA;aa=Arg;anticodon=cct	sTTR_16	yes
Chr11_3B	397659	397755	tRNA	Tb427v9_000324300:tRNA;aa=Ile;anticodon=aat	no	no
Chr11_3B	559600	559683	tRNA	Tb427v9_000329900:tRNA;aa=Gly;anticodon=ccc	no	no
Chr11_core	1863938	1864008	tRNA	Tb427v9_000410900:tRNA;aa=Gly;anticodon=ccc	sTTR_4	only ELP3b, TRF and TBP
Chr11_core	2213880	2213954	tRNA	Tb427v9_000425500:tRNA;aa=Lys;anticodon=ctt	cTTR_7	all except DOT1A
Chr11_core	3580246	3580317	tRNA	Tb427v9_000484400:tRNA;aa=His;anticodon=gtg	sTTR_7	yes
Chr11_core	3580378	3580449	tRNA	Tb427v9_000484500:tRNA;aa=Glu;anticodon=ttc	sTTR_7	yes
Chr11_core	3580509	3580581	tRNA	Tb427v9_000484600:tRNA;aa=Phe;anticodon=gaa	sTTR_7	yes
Chr11_core	3603481	3603552	tRNA	Tb427v9_000485500:tRNA;aa=Ala;anticodon=cgc	sTTR_8	yes
Chr11_core	3603618	3603691	tRNA	Tb427v9_000485600:tRNA;aa=Arg;anticodon=ccg	sTTR_8	yes
Chr11_core	3603801	3603873	tRNA	Tb427v9_000485700:tRNA;aa=Phe;anticodon=gaa	sTTR_8	yes

Supplemental Table S9

Supplemental Table S9. Primers used for N-terminal tagging

Sequences in lower case align to pPOTv4 plasmid DNA, whereas sequences in upper case align to *T. brucei* DNA. F - forward primers, R - reverse primers.

Protein name	TriTryp ID	Primer name	Primer sequence
BDF1	Tb927.10.8150	5′ UTR F	ACGGTAATGTGTGGTACGCT
		5′ UTR R	gcagcaggtctgcattatacCGTTATTCACGCCACCACC
		CDS F	ggatcaggatcgggtagtATGTCCGATACTACTAGAAGAGTG
		CDS R	AGTTGTCGACCGTGAGACTT
BDF2	Tb927.10.7420	5′ UTR F	TGTGGATTCAGTGGGTTGGG
		5′ UTR R	gcagcaggtctgcattatacTGCTCTAACGCAGATGTTCG
		CDS F	ggatcaggatcgggtagtATGAGCAAGAACGAGCGAGATAC
		CDS R	CGCAGTGTTGATTCGTCGTC
BDF3	Tb927.11.10070	5′ UTR F	GCTGTTATTGGCTACGCACTTG
		5′ UTR R	gcagcaggtctgcattatacCGATGCGCGCAATACTTACTGG
		CDS F	ggatcaggatcgggtagtATGGTCCTCACAGGGGTCAA
		CDS R	GTTCCCGGTGCTTCTCAAGT
BDF4	Tb927.7.4380	5′ UTR F	TTACTGGGTTGTGTGAGCGG
		5′ UTR R	gcagcaggtctgcattatacTGTGGAGGGACAACGAAGAG
		CDS F	ggatcaggatcgggtagtATGTCGGGGGGCACCTC
		CDS R	AAAACCTCCTCCATCAGCCG
BDF5	Tb927.11.13400	5′ UTR F	GGCACGCAGACGCATTTACT
		5′ UTR R	gcagcaggtctgcattatacGGGCACGATCGGTGTTGCT
		CDS F	ggatcaggatcgggtagtATGAGTCAAAACCGGCAGC
		CDS R	ATTGCTATCGTCCTCGCTGG
BDF6	Tb927.1.3400	5′ UTR F	GTGTAGATGCGAACGCCATT
		5′ UTR R	gcagcaggtctgcattatacATACCCCTTCTTTTTCTTTTCTTTTGAG
		CDS F	ggatcaggatcgggtagtATGCGACATGTCGATGTGTTAC
		CDS R	CGATGTTGCGACAGTTGGTG
BDF7	Tb927.11.6350	5′ UTR F	GCAAGTCGCTTCAACCAGTG
		5′ UTR R	gcagcaggtctgcattatacTCCCCCCCCTCAGGCC
		CDS F	ggatcaggatcgggtagtATGGTTGTGACCCGCTCC
		CDS R	ACCCGGAGTACCATTACACCT
PHD1	Tb927.10.9930	5′ UTR F	GTTGGAAGGAAGGGTGGGAA
		5′ UTR R	gcagcaggtctgcattatacGAATGGACTCTAGCACTGTCTCAC
		CDS F	ggatcaggatcgggtagtATGCGTATTGTAATCGATGTCGATG
		CDS R	ACGGTGTATCCCCACGAAAC
PHD2	Tb11.v5.0388	5′ UTR F	GTTGCGGTTATTCCCTCCCT
		5′ UTR R	gcagcaggtctgcattatacGCAACAAATATGACTCACTATACTTGC
		CDS F	ggatcaggatcgggtagtATGAGCACCTCAATTGAGGGA
		CDS R	CCTCACCCTCACTTTCCGAATC

Protein name	TriTryp ID	Primer number	Primer sequence
PHD3	Tb11.v5.0794	5′ UTR F	GCGGAAAGGAATTCACATGAGG
		5′ UTR R	gcagcaggtctgcattatacCACACCACCCTTTCGGCGA
		CDS F	ggatcaggatcgggtagtATGCTACCGTACATGTCGGGT
		CDS R	ACCGCTTGTGGTGACACATT
PHD4	Tb927.3.2140	5′ UTR F	ACGAGCGGGTTTAGTGAAGG
		5′ UTR R	gcagcaggtctgcattatacCGGTGTGCCTGTGTGATAAA
		CDS F	ggatcaggatcgggtagtATGAAGTTTGAGAACCCCGTTG
		CDS R	TTCTCGGTCGGCATTGGAAA
PHD5	Tb927.11.5870	5′ UTR F	ACCCTCTTCAATGCACTCCG
		5′ UTR R	gcagcaggtctgcattatacTGCAGACTACGGCAAAAGGA
		CDS F	ggatcaggatcgggtagtATGAGCACATCCCTCACGTC
		CDS R	GCACGACTTTCACCACGTTC
TDR1	Tb927.11.14190	5′ UTR F	CTTATCGGCTGCTTTCGCAC
		5′ UTR R	gcagcaggtctgcattatacTTGAGTATGTCCTCTGTTACCCC
		CDS F	ggatcaggatcgggtagtATGTCTTTTCTTGTGTATGCCG
		CDS R	AGTAACCTGTGCATCGCCAA
CRD1	Tb11.v5.0267	5′ UTR F	GATGTATGGCGCAGAAGGGT
		5′ UTR R	gcagcaggtctgcattatacGAGTTCCGGTTGCAACTGATTG
		CDS F	ggatcaggatcgggtagtATGTCCATCCACCAAACAAAAGTC
		CDS R	TGACCACTCTCCCAAGCCTA
TFIIS2-2	Tb927.2.3480	5′ UTR F	GGGCGTAATAACGAGGTGGA
		5′ UTR R	gcagcaggtctgcattatacTTTTTTTCTTTTCCCCCCGTGG
		CDS F	ggatcaggatcgggtagtATGATTCCATCATTTGCTCCAGG
		CDS R	CCTTCGTCTTCTTTCCGCCT
ZCW1	Tb927.10.11720	5′ UTR F	TAACGACACTGCAAATCCCT
		5′ UTR R	gcagcaggtctgcattatacATATATATATATAACAAATATGCACAAC
		CDS F	ggatcaggatcgggtagtATGCTAACTCGAAGAGCCGC
		CDS R	TCACCTCAGGTTCGCTTTCC
EAF6	Tb927.9.2910	5′ UTR F	AGCGTGAGACAGGATGATTTCGAC
		5′ UTR R	gcagcaggtctgcattatacGCAACCAGCTCGCGCCTCTAAA
		CDS F	ggatcaggatcgggtagtATGTCCGGTAGGGGTCATGG
		CDS R	TTTCACCCGCTCAACAGTCC
HAT1	Tb927.7.4560	5′ UTR F	CGATGTGAGGTGGGTGGAAA
		5′ UTR R	gcagcaggtctgcattatacGTCCAAAGGTGTGACACCTGG
		CDS F	ggatcaggatcgggtagtATGGTAATGTTTGAGGTGCGG
		CDS R	CGACTATCTTGGCGCAGTTG

Protein name	TriTryp ID	Primer number	Primer sequence
HAT2	Tb927.11.11530	5′ UTR F	GGCCGCTTATTCTGTCGGTT
		5′ UTR R	gcagcaggtctgcattatacTGCGCAACCTCTCGCATC
		CDS F	ggatcaggatcgggtagtATGGCGTCGTTAGCAGCAA
		CDS R	TGAAGTATCGCACTGTGGTGG
HAT3	Tb927.10.8310	5′ UTR F	GGAAACCGTGGGATTTTGCC
		5′ UTR R	gcagcaggtctgcattatacTCCACCTATACGGAAGGGAATTAATC
		CDS F	ggatcaggatcgggtagtATGAAACGACAGAGGTCGGG
		CDS R	GTGGCAACACCATTAGGCAC
ELP3a	Tb927.8.5770	5′ UTR F	TAGCGTGCTCAATGTGGGAG
		5′ UTR R	gcagcaggtctgcattatacTCTAACCTCAGTCACCTGAACAAC
		CDS F	ggatcaggatcgggtagtATGCTACACGCTAGTGGTAATGG
		CDS R	CGGGCTCGTTGGGACAATA
ELP3b	Tb927.8.3310	5′ UTR F	ACCGAGATTACTGGAGGACCA
		5′ UTR R	gcagcaggtctgcattatacTCTTCTACGGTGTTCAAAAGGCC
		CDS F	ggatcaggatcgggtagtATGTCGCTAAAGCGCCCTC
		CDS R	TCTTCCACAGCCGCAGATTC
ELP3c	Tb11.v5.0520	5′ UTR F	CTTTCGCCTTGTCTCTCCGT
		5′ UTR R	gcagcaggtctgcattatacAAACACCGTTACAACAAGTACACC
		CDS F	ggatcaggatcgggtagtATGGGACCCGGCCAGTTT
		CDS R	GAAGTTGGGCAGTCACGGTT
NAT2	Tb927.1.4490	5′ UTR F	GAAATCGGTGTTGGCAGGATG
		5′ UTR R	gcagcaggtctgcattatacGCCTGTGATGAGATGTTATCCCT
		CDS F	ggatcaggatcgggtagtATGACTTCCGCGTACCGCA
		CDS R	CATGCGACCCATCAACTGGT
NAT3	Tb927.10.12830	5′ UTR F	GAAGGGGCGTTTAATAGGAAGG
		5′ UTR R	gcagcaggtctgcattatacTTACACCTCATGGATGAATAAAAAAGAG
		CDS F	ggatcaggatcgggtagtATGAGCGCAGTGGAGCTTG
		CDS R	CCACCATTTCCTGCCCGAAT
LPLAT1	Tb927.5.2280	5′ UTR F	ACGTTGCTGCGATGGAAAGA
		5′ UTR R	gcagcaggtctgcattatacCGCAACCGAAAACACCAAGATC
		CDS F	ggatcaggatcgggtagtATGGCACTCACTGCACGT
		CDS R	CTGCATCGACCACCCGAATA
SET1	Tb11.v5.0422	5′ UTR F	ACAGTACCCCGTTTGCTGAG
		5′ UTR R	gcagcaggtctgcattatacGCAACCAAAAAAAAAAAGGAGCTAC
		CDS F	ggatcaggatcgggtagtATGCAAACAGAAGCCCTGC
		CDS R	AGTAGACCGTGGTAACGGGA

Protein name	TriTryp ID	Primer number	Primer sequence
SET3	Tb927.1.4720	5′ UTR F	ATTACATGGAGGGGATGCCG
		5′ UTR R	gcagcaggtctgcattatacGGCAACTGTTGTGCCCAC
		CDS F	ggatcaggatcgggtagtATGGAGGAGTTGCGTAAACG
		CDS R	TGGCGGCAAAATCTCAGCTA
SET4	Tb927.10.11130	5′ UTR F	CCACTTTCCCTTAGCCCGAT
		5′ UTR R	gcagcaggtctgcattatacCCCAGGTGGGTTGGCAAC
		CDS F	ggatcaggatcgggtagtATGTCATGGGTGGAAGACAAGTC
		CDS R	TAAGCGTTACGTGAGCCTCG
SET5	Tb927.10.12880	5′ UTR F	GAGTGTGAGGAACGGAAGCA
		5′ UTR R	gcagcaggtctgcattatacCCTTTTCCACTTTCTCCCCC
		CDS F	ggatcaggatcgggtagtATGCCGACTCAGAGCCCC
		CDS R	GCCACAAAAGAGCTTCCACG
SET6	Tb927.10.3730	5′ UTR F	TCAAAGCGGAGAAGGAAGGG
		5′ UTR R	gcagcaggtctgcattatacCAAAAGCACAACAAGAGCCG
		CDS F	ggatcaggatcgggtagtATGCGTCCGTATTTCCCAGG
		CDS R	GAGTTGCGTTGTTGGTGGTG
SET7	Tb927.10.4600	5′ UTR F	GCGCTCACGGTTCTACTTGT
		5′ UTR R	gcagcaggtctgcattatacCCTGTGTTGCAGGGCTTCT
		CDS F	ggatcaggatcgggtagtATGAGCAAGGCAGATCTTGTTG
		CDS R	GCATTCCGCAGTTTTACGGG
SET8	Tb927.10.8060	5′ UTR F	ATTGCCTCCCTTCACCCATC
		5′ UTR R	gcagcaggtctgcattatacACCAACAAACCCAGCCGAC
		CDS F	ggatcaggatcgggtagtATGGTTCTCCCCTCACCACA
		CDS R	TCATACGCACCCTTCTGCTC
SET9	Tb927.10.9680	5′ UTR F	GGAGGTCAGGTGTGTTCGTT
		5′ UTR R	gcagcaggtctgcattatacTTCACAACTAGGCTAGAGTGTG
		CDS F	ggatcaggatcgggtagtATGAGTGTTTGCCTTGGCAC
		CDS R	TCGAGCAGTTGTTGCGAAAG
SET10	Tb927.11.13560	5′ UTR F	ACGCTCGTATGTGTACCTCG
		5′ UTR R	gcagcaggtctgcattatacCTTCACACGACGGCTCAG
		CDS F	ggatcaggatcgggtagtATGCGATGTTGTCGGACAATG
		CDS R	GCAGACGCCCTTTTCTCTTG
SET11	Tb927.11.5120	5′ UTR F	TGTTTAGGATGCTTGCTCCA
		5′ UTR R	gcagcaggtctgcattatacATACTTCAACCTTTGTATTCTCAAT
		CDS F	ggatcaggatcgggtagtATGCAGTTAGCTGAGGAAC
		CDS R	CCTCCACATCCTCACTTGTTT

Protein name	TriTryp ID	Primer number	Primer sequence
SET12	Tb927.3.750	5′ UTR F	AACAAGGAGGGCTACGAGTG
		5′ UTR R	gcagcaggtctgcattatacGCTTTATGAAAAACAGCCAGTTC
		CDS F	ggatcaggatcgggtagtATGCGGCTGCGGGGTCAA
		CDS R	CCCAGGCACACGAACCTCTT
SET13	Tb927.4.2440	5′ UTR F	ATTGTGGCGGTCTCCTCTTG
		5′ UTR R	gcagcaggtctgcattatacGGTGGCGAATGTTCCTTCC
		CDS F	ggatcaggatcgggtagtATGTTTTCACAAACGCGCTG
		CDS R	TGATGCGCTCCATCTCTTCC
SET14	Tb927.4.3310	5′ UTR F	CGGGGAGAAGTGAAAAGGCT
		5′ UTR R	gcagcaggtctgcattatacTTCACTAAGCTTCCCGCTACTG
		CDS F	ggatcaggatcgggtagtATGAGTGGTGGTGTGTCCC
		CDS R	GTAAAGGCGGCATGAGCATC
SET15	Tb927.5.2770	5′ UTR F	ATTTGCCTTCGATCCCCTCC
		5′ UTR R	gcagcaggtctgcattatacACCTTTTAATTTCCTTTCCCCCT
		CDS F	ggatcaggatcgggtagtATGACGGGGGCGGACGA
		CDS R	TTCGCACAAGACAAGGTGCT
SET16	Tb927.5.3500	5′ UTR F	TCCCTCCCCTCTCTTTTCCC
		5′ UTR R	gcagcaggtctgcattatacCTTTCCAGAAACGGATATGAACGAG
		CDS F	ggatcaggatcgggtagtATGGAGAGCGTGTCGCTTATC
		CDS R	AATCTGCATTTCCGCCTCCA
SET17	Tb927.6.3610	5′ UTR F	TGTCACAAGCGTACTGCACA
		5′ UTR R	gcagcaggtctgcattatacGCCGTAACTTGCGGTAAAAGG
		CDS F	ggatcaggatcgggtagtATGACGCTGCTTCACATGTTG
		CDS R	TTTGACCCACACCCACCTTC
SET18	Tb927.6.910	5′ UTR F	ACCTCGCTGAGTTGTTACGG
		5′ UTR R	gcagcaggtctgcattatacATTAGGTCCACCTGCCCCA
		CDS F	ggatcaggatcgggtagtATGGTACTTGGTGGTGTAGTTGG
		CDS R	CCGATGTTGAGTTGCCTCCT
SET19	Tb927.7.5620	5′ UTR F	TTGGTTTCCCGATTGCCTGT
		5′ UTR R	gcagcaggtctgcattatacGCAAGGGCTAACCGACCCT
		CDS F	ggatcaggatcgggtagtATGGTGAGGCTTCGCTCG
		CDS R	ACGACCGTAGATGCTTTGCT
SET20	Tb927.8.2490	5′ UTR F	CGGGGGAAACAACTGACGAT
		5′ UTR R	gcagcaggtctgcattatacATAAGTACAGTGCAGGTGGCG
		CDS F	ggatcaggatcgggtagtATGCCAGTGTTTGAGAACAAGC
		CDS R	CTTGGCCCTCTGCATTCTCA

Protein name	TriTryp ID	Primer number	Primer sequence
SET21	Tb927.8.2690	5′ UTR F	GATGTGGTCATGCGTGGTTG
		5′ UTR R	gcagcaggtctgcattatacAGCCCAATGATACTGGGCC
		CDS F	ggatcaggatcgggtagtATGCTCCAGCAATTATCCGC
		CDS R	GCGTTGTTTCTCTGGCGAAG
SET22	Tb927.8.2710	5′ UTR F	CAGCACGCTTCATCCCTACA
		5′ UTR R	gcagcaggtctgcattatacTTTTGTTGCCAAATTACGTATGTAC
		CDS F	ggatcaggatcgggtagtATGGCCACTGTCACGGAG
		CDS R	GAGCCAGTTCGTTGGTGAGA
SET23	Tb927.8.6470	5′ UTR F	TCACTACAAGGCCGGTTGTC
		5′ UTR R	gcagcaggtctgcattatacTATCCGGTCATATATATATATATAT
		CDS F	ggatcaggatcgggtagtATGCCTGGGTGTCTTGAGG
		CDS R	TTCGACAGCACATTCCGTGA
SET24	Tb927.8.6530	5′ UTR F	GAGGGGAAAGTGCTGCTCAT
		5′ UTR R	gcagcaggtctgcattatacTCGCTACGCCTTTCCACC
		CDS F	ggatcaggatcgggtagtATGAACAGGGGGTCGGTG
		CDS R	TCTTGAGGTTTTCCCTCGCC
SET25	Tb927.9.1510	5′ UTR F	GGTGCAAAGGAATGACGGTG
		5′ UTR R	gcagcaggtctgcattatacCGCTATCGGTCGTGAAAACTAC
		CDS F	ggatcaggatcgggtagtATGCCGGCCAAACCCAC
		CDS R	CCAACACTGCTGTAACCGGA
SET26	Tb927.10.8100	5′ UTR F	GGTTACCCGAAAGCAGTGGA
		5′ UTR R	gcagcaggtctgcattatacGCACTGTGGCAACTAATCACG
		CDS F	ggatcaggatcgggtagtATGATATGCAAGGTTTTGGAGCG
		CDS R	TACTCACACATCACGGTGCC
SET27	Tb927.9.13470	5′ UTR F	TCTCGACTATGCGGTTCGTG
		5′ UTR R	gcagcaggtctgcattatacTCCACACGAGGAAACACTGC
		CDS F	ggatcaggatcgggtagtATGGATGTGCATTCCCCGTTAC
		CDS R	TCGTTCGCAAACCGACCATA
SET28	Tb927.9.10100	5′ UTR F	AATGGTTTGCCGGGACATCT
		5′ UTR R	gcagcaggtctgcattatacTTCTCCTCGCTCTTAGTTTATCCG
		CDS F	ggatcaggatcgggtagtATGGTAATGAAAGCCAATTTCTGC
		CDS R	TTTGGTTGGCAAGAATGCCG
SET29	Tb927.3.3370	5′ UTR F	TGAGGCGCATGATCCACTTT
		5′ UTR R	gcagcaggtctgcattatacACCAACTTGGAGTACAAAGAAGAC
		CDS F	ggatcaggatcgggtagtATGTCTGAGGTGGTACCTACTCG
		CDS R	AAAGCGGCAGGGACTTCTAC

Protein name	TriTryp ID	Primer number	Primer sequence
SET30	Tb927.3.1860	5′ UTR F	CACAATAAGTGCCATGCGGT
		5′ UTR R	gcagcaggtctgcattatacCCCTTTTCTTATACTCCTTCACG
		CDS F	ggatcaggatcgggtagtATGTACAGAATAGCAATTCGACG
		CDS R	TTACCTCCAGGATGAGCGGA
DOT1A	Tb927.8.1920	5′ UTR F	GATGCGCCAAAACCATCTAC
		5′ UTR R	gcagcaggtctgcattatacAGATGTATGTAGTTTATAACTATTCTCAC
		CDS F	ggatcaggatcgggtagtATGCCTGGATTGCTAATATCCCG
		CDS R	TTCCAACACAACGAGCACCA
DOT1B	Tb927.1.570	5′ UTR F	AGAACTTACGCCGCACACTT
		5′ UTR R	gcagcaggtctgcattatacTAGTGTCACTTCAGAACCGTAAATCG
		CDS F	ggatcaggatcgggtagtATGGACGCACGTGTTCATCG
		CDS R	CATCCAACTTCCGACCACGG
DOT1	Tb927.11.3200	5′ UTR F	ACCTTCTACCCGTTTGCAGT
		5′ UTR R	gcagcaggtctgcattatacCCACTAGTCAACAACGTCCG
		CDS F	ggatcaggatcgggtagtATGACCTTTTTCTACGCTGCAG
		CDS R	CACACATTTCCAACGCCACT
HDAC1	Tb927.10.1680	5′ UTR F	TCTTGTTTCCCTCTGCGTCC
		5′ UTR R	gcagcaggtctgcattatacTTGAGAAACTGTACGAACCTTCTAGG
		CDS F	ggatcaggatcgggtagtATGAATGAGGATGGTGATTGTCGG
		CDS R	GATGGCCTGTACCCGGAAAA
HDAC2	Tb927.11.15600	5′ UTR F	GCTACTGGTGGAGCATGTGT
		5′ UTR R	gcagcaggtctgcattatacAGCTGGGCATGCAATCAGT
		CDS F	ggatcaggatcgggtagtATGGTTGTGGAGCCTCCTG
		CDS R	TGACTCGTCGTGGAACACAG
HDAC3	Tb927.2.2190	5′ UTR F	CCTGCCATATTGTGGGGGTT
		5′ UTR R	gcagcaggtctgcattatacTGCGGTTGCGGTTCAAC
		CDS F	ggatcaggatcgggtagtATGGGCAAAGAAACGCGTG
		CDS R	TTGCGGTGATGTCCAGAGTG
HDAC4	Tb927.5.2900	5′ UTR F	ATAGACACATCGGCGGAGTT
		5′ UTR R	gcagcaggtctgcattatacACGATAGAGTTGACTAAGTAAACAC
		CDS F	ggatcaggatcgggtagtATGTCTGTGAGGGAACGG
		CDS R	TGACACGTCCGGTAGAAACA
Sir2rp1	Tb927.7.1690	5′ UTR F	GCTGAAGAGGTGCCTACGTT
		5′ UTR R	gcagcaggtctgcattatacTTCACGTTTCCCTATACTAGTTGG
		CDS F	ggatcaggatcgggtagtATGACAGAACCGAAGTTAGCAAC
		CDS R	CATGCCCTTCTTGGCGAGTA

Protein name	TriTryp ID	Primer number	Primer sequence
Sir2rp2	Tb927.8.3140	5′ UTR F	TGCGCCCATGAGTTGTAGTT
		5′ UTR R	gcagcaggtctgcattatacATCACTCAACTGCACTAGCTTAAAC
		CDS F	ggatcaggatcgggtagtATGGCTGACCGCCTTGCT
		CDS R	CTCGTAGTCGCCATCAGGTC
Sir2rp3	Tb927.4.2520	5′ UTR F	TGGATGAACCGACTGCACAA
		5′ UTR R	gcagcaggtctgcattatacTGTTGAACCTACAAACAGTCCTTC
		CDS F	ggatcaggatcgggtagtATGAGGCGGCCCAATGGT
		CDS R	TCTACGACTCCGCAGCATTC
JMJ1	Tb927.11.1760	5′ UTR F	ACCAGCATTGGGATCGGTTT
		5′ UTR R	gcagcaggtctgcattatacAGCCAACAGAAACTTGCAAAAATATATAC
		CDS F	ggatcaggatcgggtagtATGCTGGAACTGGACGGAC
		CDS R	CCCTCCAGCGTTCAATGACT
JMJ2	Tb927.11.2000	5′ UTR F	TTGGCTGAAGAGCATGCAGA
		5′ UTR R	gcagcaggtctgcattatacGTGTGAAACACTTCAAAATAAAAATAAGAC
		CDS F	ggatcaggatcgggtagtATGGCGCAGAGCTGGTTC
		CDS R	CGGACGATGGGACAAGTTCA
LCM1	Tb927.9.12780	5′ UTR F	ATCGAAGACACGACGGCTTT
		5′ UTR R	gcagcaggtctgcattatacTAAGGTGTTTTGTGAGGTAGCAAC
		CDS F	ggatcaggatcgggtagtATGTCCATTGCGGTAGAAGAGAG
		CDS R	CTTGGATTGCATAACGGCGG
CLD1	Tb927.7.660	5′ UTR F	GATTTGCTTGGGTGGTGCTG
		5′ UTR R	gcagcaggtctgcattatacCGCATGAACTTCCTGCAGTTTTTG
		CDS F	ggatcaggatcgggtagtATGCCAAATGAAAAGGCGCAG
		CDS R	TGCATCCGATTCCGTCACAA
AGO1	Tb927.10.10850	5′ UTR F	TAGCAGCAAAATGATGGCAC
		5′ UTR R	gcagcaggtctgcattatacTTTATTTAAATCCTTTTTATTAGGTTGC
		CDS F	ggatcaggatcgggtagtATGTCTGACTGGGAACGTGG
		CDS R	GGTTTGTCCATGCCTCCACT
DMT	Tb927.3.1360	5′ UTR F	TAAAAGTCCCGCAGGCTCAA
		5′ UTR R	gcagcaggtctgcattatacCAACGGTCCGTAACTGTGAC
		CDS F	ggatcaggatcgggtagtATGTGCTCTGTTGCACCGTTAG
		CDS R	TGCGTAACACTGCTGCTCTT
KKT2	Tb927.11.10520	5′ UTR F	TTACGAGGGGTGGTAGTGGT
		5′ UTR R	gcagcaggtctgcattatacGACTGTGCCCCGTCCCTT
		CDS F	ggatcaggatcgggtagtATGTTCAATGTCTCACCAGCG
		CDS R	CACGTACACTCGACCAAACC

Protein name	TriTryp ID	Primer number	Primer sequence
NOC1	Tb927.11.2120	5′ UTR F	CATACGCGTGCGATTTGGT
		5′ UTR R	gcagcaggtctgcattatacTTTTTAAGGGATGTTTATATGCCTATTTTAGG
		CDS F	ggatcaggatcgggtagtATGACTCGAGAAGAGGAGGTATTG
		CDS R	TGTGCGCTTCAGGTAATCCT
NUP110/MLP1	Tb927.11.330	5′ UTR F	TCTTGTCGTGAGAGGCAACC
		5′ UTR R	gcagcaggtctgcattatacGATTCCAACCTTTGCACAGTGG
		CDS F	ggatcaggatcgggtagtATGTTCCCTGGGTCAAAAAGG
		CDS R	TTTCTGCCGAGAGTCATCCG
TRF	Tb927.10.12850	5′ UTR F	GGCTGAAGCGAAACTTCGTG
		5′ UTR R	gcagcaggtctgcattatacTGCCCCCCTGGAATTACGT
		CDS F	ggatcaggatcgggtagtATGTACTGTCACGCTGGCG
		CDS R	CTCCCATGCAAGGGGAAGAG
TBP/TRF4	Tb927.10.15950	5′ UTR F	AGAAGGGTGTTTCTCAGCGG
		5′ UTR R	gcagcaggtctgcattatacCGTTCTTTTTGTTTCCTCCTCAGG
		CDS F	ggatcaggatcgggtagtATGGACAATGACTTCACTGGAAAC
		CDS R	TGCCAAGAAGACCAGAGGTG

References

Alsford S, Kawahara T, Isamah C, Horn D. 2007. A sirtuin in the African trypanosome is involved in both DNA repair and telomeric gene silencing but is not required for antigenic variation. *Mol Microbiol* **63**: 724-736.

Alsford S, Turner DJ, Obado SO, Sanchez-Flores A, Glover L, Berriman M, Hertz-Fowler C, Horn D. 2011. High-throughput phenotyping using parallel sequencing of RNA interference targets in the African trypanosome. *Genome Res* **21**: 915-924.

Baron DM, Ralston KS, Kabututu ZP, Hill KL. 2007. Functional genomics in Trypanosoma brucei identifies evolutionarily conserved components of motile flagella. *J Cell Sci* **120**: 478-491.

Branche C, Kohl L, Toutirais G, Buisson J, Cosson J, Bastin P. 2006. Conserved and specific functions of axoneme components in trypanosome motility. *J Cell Sci* **119**: 3443-3455.

D'Archivio S, Wickstead B. 2017. Trypanosome outer kinetochore proteins suggest conservation of chromosome segregation machinery across eukaryotes. *J Cell Biol* **216**: 379-391.

Dean S, Moreira-Leite F, Varga V, Gull K. 2016. Cilium transition zone proteome reveals compartmentalization and differential dynamics of ciliopathy complexes. *Proc Natl Acad Sci U S A* **113**: E5135-5143.

ElBashir R, Vanselow JT, Kraus A, Janzen CJ, Siegel TN, Schlosser A. 2015. Fragment ion patchwork quantification for measuring site-specific acetylation degrees. *Anal Chem* **87**: 9939-9945.

Fritz M, Vanselow J, Sauer N, Lamer S, Goos C, Siegel TN, Subota I, Schlosser A, Carrington M, Kramer S. 2015. Novel insights into RNP granules by employing the trypanosome's microtubule skeleton as a molecular sieve. *Nucleic Acids Res* **43**: 8013-8032.

Gheiratmand L, Brasseur A, Zhou Q, He CY. 2013. Biochemical characterization of the bi-lobe reveals a continuous structural network linking the bi-lobe to other single-copied organelles in Trypanosoma brucei. *J Biol Chem* **288**: 3489-3499.

Goos C, Dejung M, Janzen CJ, Butter F, Kramer S. 2017. The nuclear proteome of Trypanosoma brucei. *PLoS One* **12**: e0181884.

Guther ML, Urbaniak MD, Tavendale A, Prescott A, Ferguson MA. 2014. High-confidence glycosome proteome for procyclic form Trypanosoma brucei by epitope-tag organelle enrichment and SILAC proteomics. *J Proteome Res* **13**: 2796-2806.

Halliday C, Billington K, Wang Z, Madden R, Dean S, Sunter JD, Wheeler RJ. 2019. Cellular landmarks of Trypanosoma brucei and Leishmania mexicana. *Mol Biochem Parasitol* **230**: 24-36.

Ingram AK, Horn D. 2002. Histone deacetylases in Trypanosoma brucei: two are essential and another is required for normal cell cycle progression. *Mol Microbiol* **45**: 89-97.

Jehi SE, Li X, Sandhu R, Ye F, Benmerzouga I, Zhang M, Zhao Y, Li B. 2014. Suppression of subtelomeric VSG switching by Trypanosoma brucei TRF requires its TTAGGG repeat-binding activity. *Nucleic Acids Res* **42**: 12899-12911.

Kovarova J, Horakova E, Changmai P, Vancova M, Lukes J. 2014. Mitochondrial and nucleolar localization of cysteine desulfurase Nfs and the scaffold protein Isu in Trypanosoma brucei. *Eukaryot Cell* **13**: 353-362.

Kramer S, Bannerman-Chukualim B, Ellis L, Boulden EA, Kelly S, Field MC, Carrington M. 2013. Differential localization of the two T. brucei poly(A) binding proteins to the nucleus and RNP granules suggests binding to distinct mRNA pools. *PLoS One* **8**: e54004.

Mony BM, MacGregor P, Ivens A, Rojas F, Cowton A, Young J, Horn D, Matthews K. 2014. Genome-wide dissection of the quorum sensing signalling pathway in Trypanosoma brucei. *Nature* **505**: 681-685.

Nanavaty V, Sandhu R, Jehi SE, Pandya UM, Li B. 2017. Trypanosoma brucei RAP1 maintains telomere and subtelomere integrity by suppressing TERRA and telomeric RNA:DNA hybrids. *Nucleic Acids Res* **45**: 5785-5796.

Ohshima S, Ohashi-Suzuki M, Miura Y, Yabu Y, Okada N, Ohta N, Suzuki T. 2010. TbUNC119 and its binding protein complex are essential for propagation, motility, and morphogenesis of Trypanosoma brucei procyclic form cells. *PLoS One* **5**: e15577.

Pandey M, Huang Y, Lim TK, Lin Q, He CY. 2020. Flagellar targeting of an arginine kinase requires a conserved lipidated protein intraflagellar transport (LIFT) pathway in Trypanosoma brucei. *J Biol Chem* **295**: 11326-11336.

Ralston KS, Lerner AG, Diener DR, Hill KL. 2006. Flagellar motility contributes to cytokinesis in Trypanosoma brucei and is modulated by an evolutionarily conserved dynein regulatory system. *Eukaryot Cell* **5**: 696-711.

Smid O, Horakova E, Vilimova V, Hrdy I, Cammack R, Horvath A, Lukes J, Tachezy J. 2006. Knock-downs of iron-sulfur cluster assembly proteins IscS and IscU down-regulate the active mitochondrion of procyclic Trypanosoma brucei. *J Biol Chem* **281**: 28679-28686.

Wang R, Gao J, Zhang J, Zhang X, Xu C, Liao S, Tu X. 2019. Solution structure of TbTFIIS2-2 PWWP domain from Trypanosoma brucei and its binding to H4K17me3 and H3K32me3. *Biochem J* **476**: 421-431.
