# Supplementary material for: A systematic analysis of Trypanosoma brucei chromatin factors identifies novel protein interaction networks associated with sites of transcription initiation and termination
Source: Genome Res. 2021 Nov;31(11):2138–54. doi: 10.1101/gr.275368.121 (PMC8559703; doi:10.1101/gr.275368.121)
Supplement: Supplemental Material [file supp_gr.275368.121_Supplemental_Figures.pdf]

Supplemental Figures for

A systematic analysis of *Trypanosoma brucei* chromatin factors identifies novel protein interaction networks associated with sites of transcription initiation and termination

Desislava P. Staneva*, Roberta Carloni*, Tatsiana Auchynnikava Pin Tong, Juri Rappsilber, A. Arockia Jeyaprakash, Keith R. Matthews[†] and Robin C. Allshire[†]

* These authors contributed equally to this work.

[†] Co-corresponding authors:

Robin Allshire: robin.allshire@ed.ac.uk

Keith Matthews: keith.matthews@ed.ac.uk

Contents

Page

1 Supplemental Fig. S1. Domain architecture of the candidate proteins.

8 Supplemental Fig. S2. Cellular localization of *T. brucei* proteins with no specific genomic enrichment by ChIP-seq.

11 Supplemental Fig. S3. Comparison of different imaging procedures.

13 Supplemental Fig. S4. Localization of YFP-tagged proteins at different stages of the cell cycle.

29 Supplemental Fig. S5. YFP-tagged proteins with no specific genomic enrichment by ChIP-seq.

31 Supplemental Fig. S6. Examples of ChIP-seq enrichment of TSR- and RNAPIII/TTR-associated factors.

33 Supplemental Fig. S7. Average metagene plots.

35 Supplemental Fig. S8. Proteomic analysis of affinity selections for several non-chromatin associated proteins.

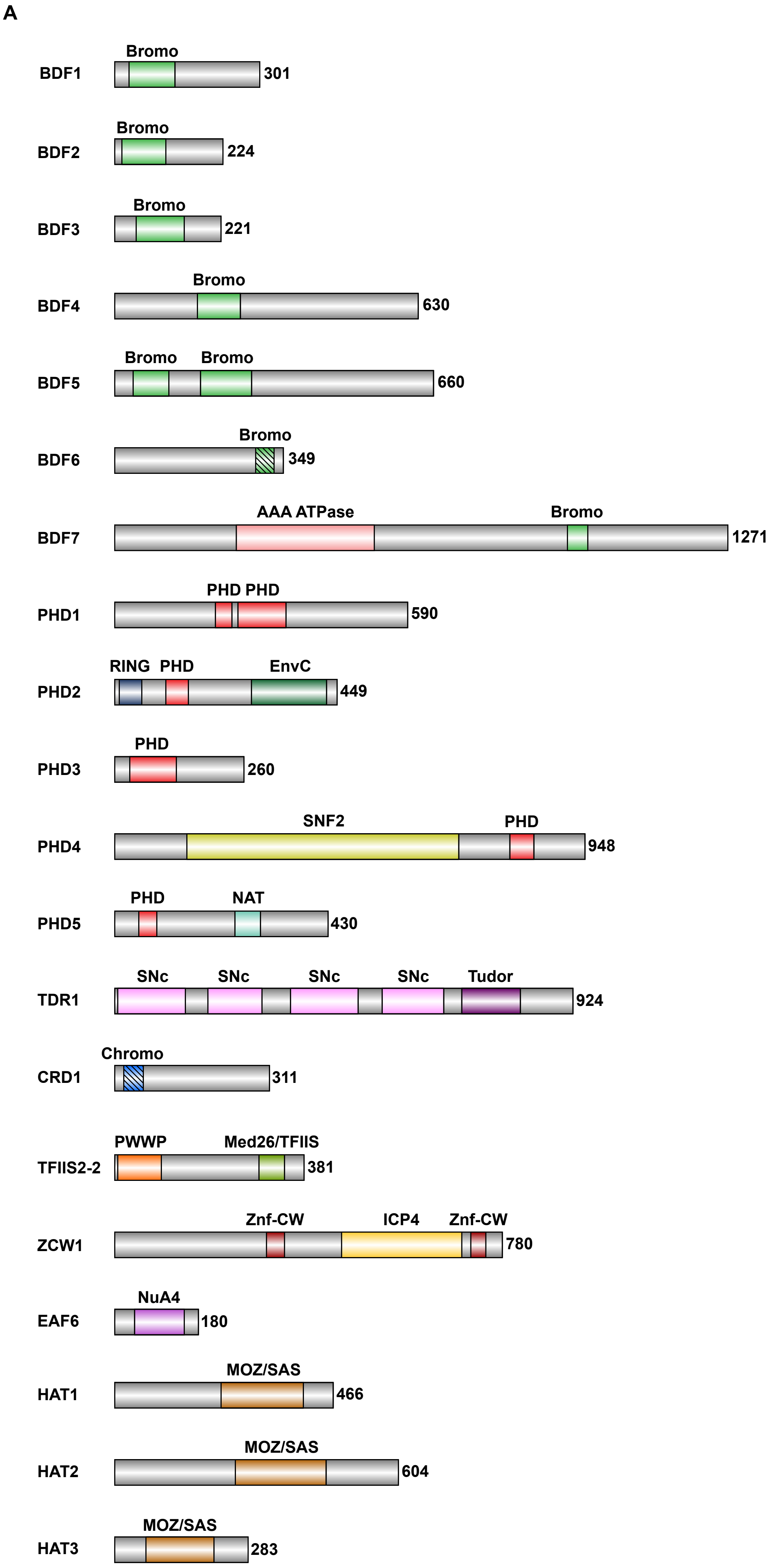
A
BDF1
Bromo
301
BDF2
Bromo
224
BDF3
Bromo
221
BDF4
Bromo
630
BDF5
Bromo
Bromo
660
BDF6
Bromo
349
BDF7
AAA ATPase
Bromo
1271
PHD1
PHD PHD
590
PHD2
RING
PHD
EnvC
449
PHD3
PHD
260
PHD4
SNF2
PHD
948
PHD5
PHD
NAT
430
TDR1
SNc
SNc
SNc
SNc
Tudor
924
CRD1
Chromo
311
TFIIS2-2
PWWP
Med26/TFIIS
381
ZCW1
Znf-CW
ICP4
Znf-CW
780
EAF6
NuA4
180
HAT1
MOZ/SAS
466
HAT2
MOZ/SAS
604
HAT3
MOZ/SAS
283

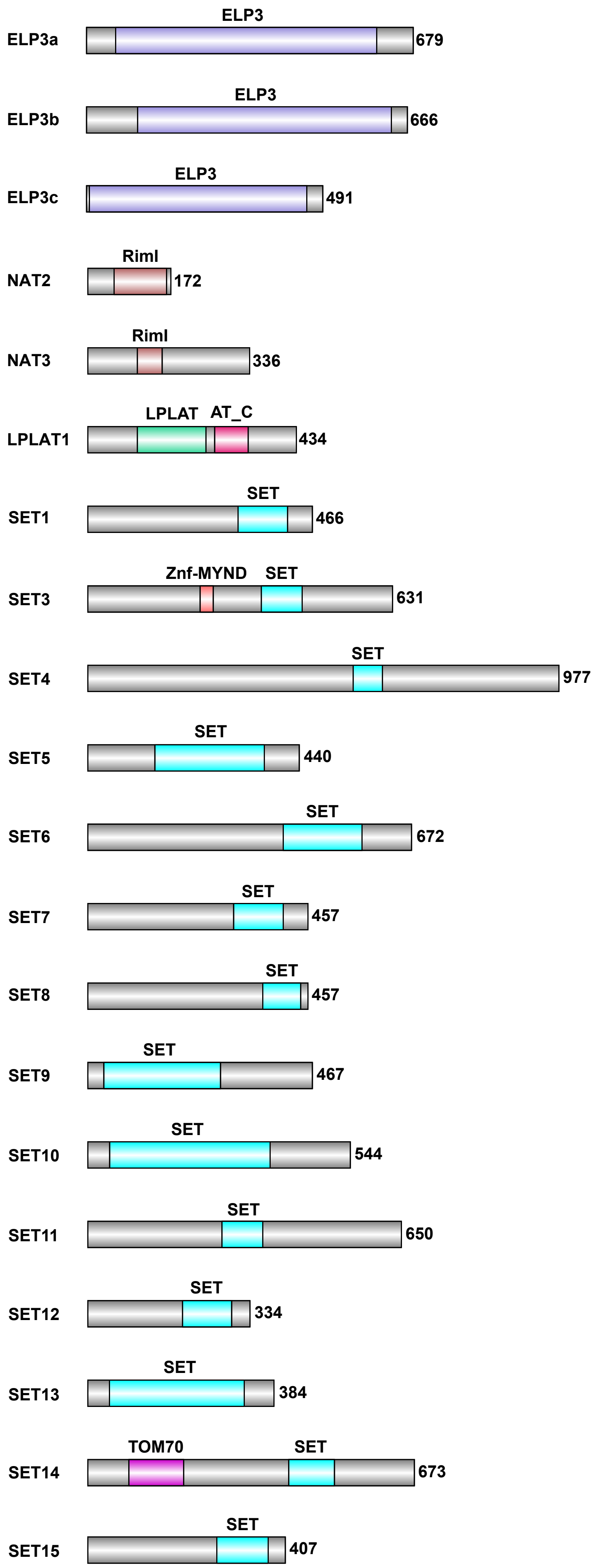
ELP3a
ELP3
679
ELP3b
ELP3
666
ELP3c
ELP3
491
NAT2
RimI
172
NAT3
RimI
336
LPLAT1
LPLAT
AT_C
434
SET1
SET
466
SET3
Znf-MYND
SET
631
SET4
SET
977
SET5
SET
440
SET6
SET
672
SET7
SET
457
SET8
SET
457
SET9
SET
467
SET10
SET
544
SET11
SET
650
SET12
SET
334
SET13
SET
384
SET14
TOM70
SET
673
SET15
SET
407

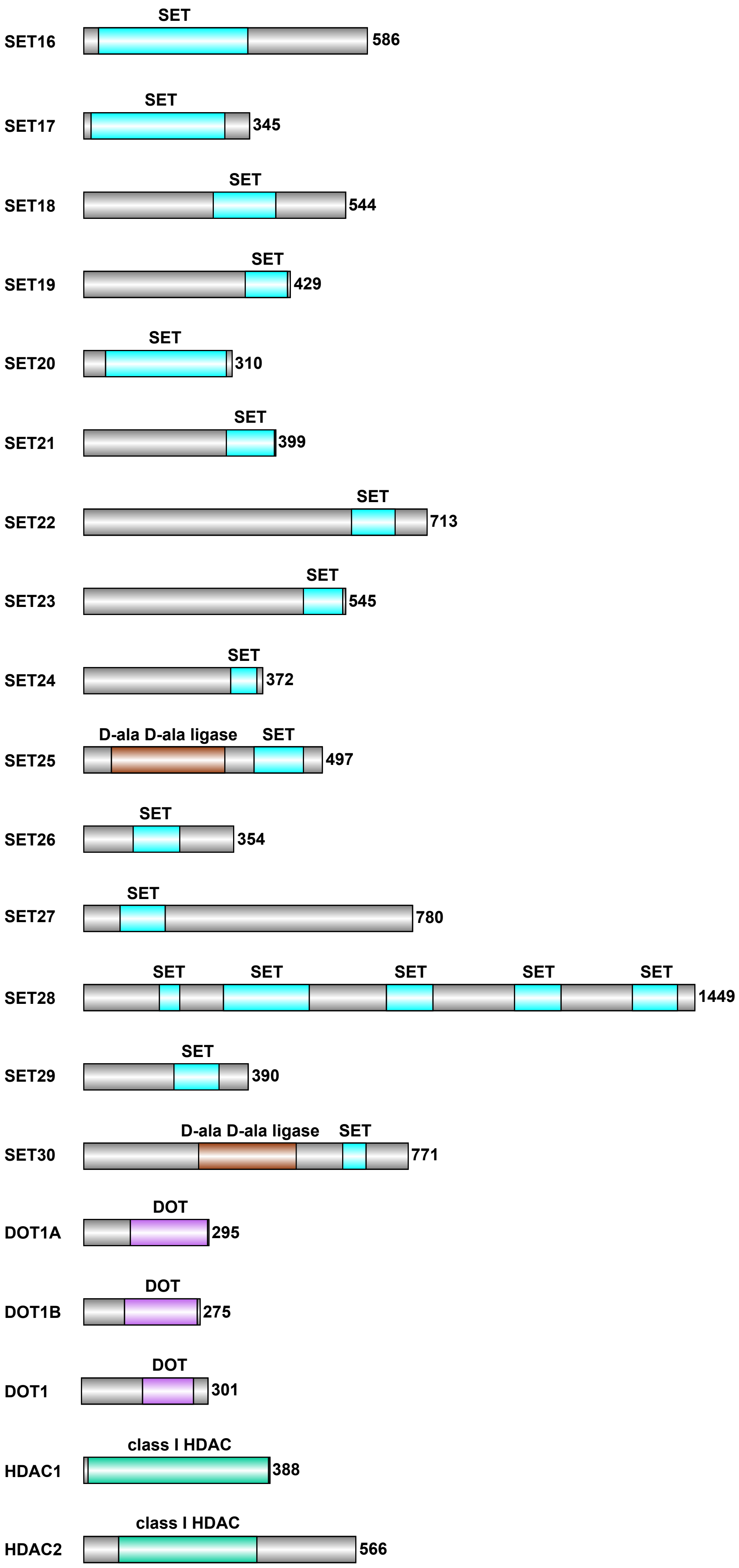

Supplemental Fig. S1 part 3

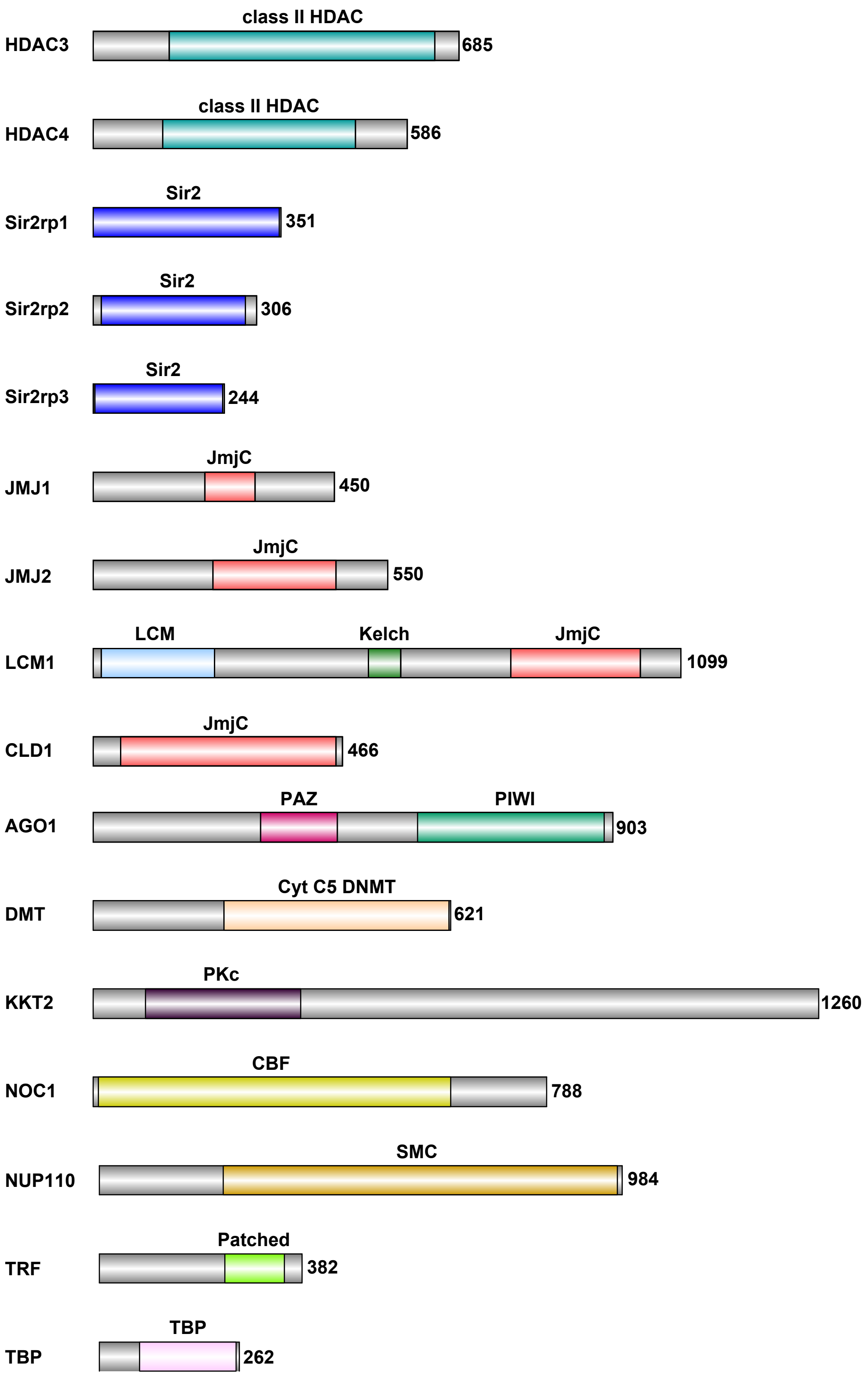
HDAC3
class II HDAC
685
HDAC4
class II HDAC
586
Sir2rp1
Sir2
351
Sir2rp2
Sir2
306
Sir2rp3
Sir2
244
JMJ1
JmjC
450
JMJ2
JmjC
550
LCM1
LCM
Kelch
JmjC
1099
CLD1
JmjC
466
AGO1
PAZ
PIWI
903
DMT
Cyt C5 DNMT
621
KKT2
PKc
1260
NOC1
CBF
788
NUP110
SMC
984
TRF
Patched
382
TBP
TBP
262

B

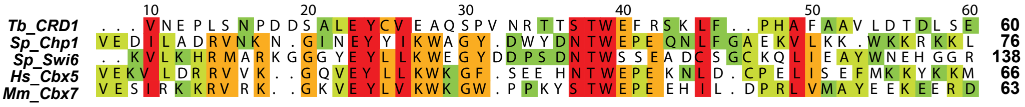
Tb_CRD1 60
Sp_Chp1 76
Sp_Swi6 138
Hs_Cbx5 66
Mm_Cbx7 63

C

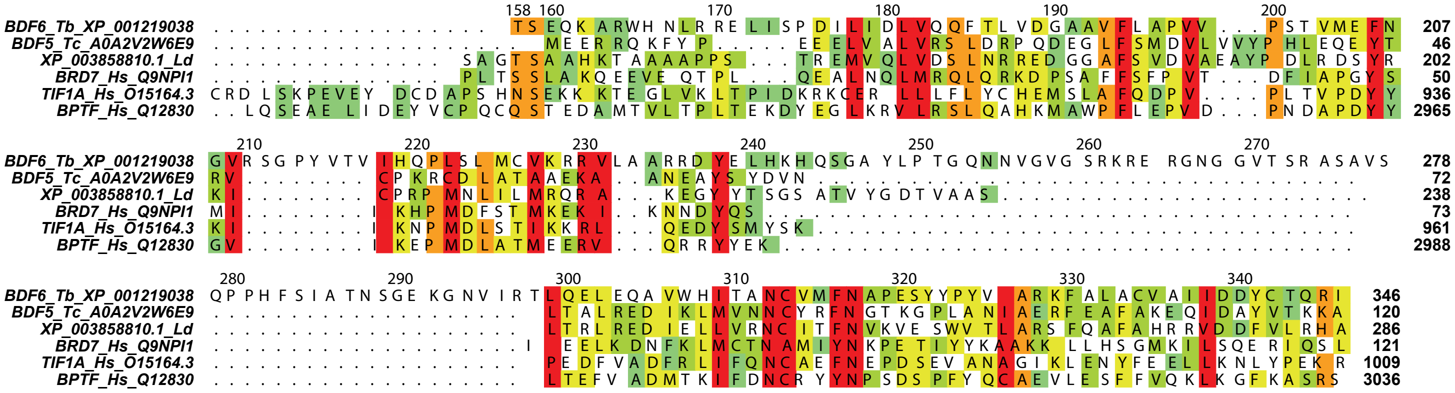
BDF6_Tb_XP_001219038
BDF5_Tc_A0A2V2W6E9
XP_003858810.1_Ld
BRD7_Hs_Q9NPI1
TIF1A_Hs_O15164.3
BPTF_Hs_Q12830

Supplemental Fig. S1. Domain architecture of the candidate proteins.

A. Conserved sequences and domains in the candidate proteins (described briefly below) were identified using NCBI's Conserved Domains tool (https://www.ncbi.nlm.nih.gov/cdd). The shaded domains of BDF6 and CRD1 are weakly predicted.

Bromo – domain which binds acetylated lysines; found in chromatin-associated proteins and in histone acetyltransferases;

AAA ATPase – has chaperone-like functions that aid assembly, function or disassembly of protein complexes;

PHD (plant homeodomain) - a Cys_4-His-Cys_3 zinc finger motif found in nuclear proteins involved in chromatin-mediated transcriptional regulation; some PHD fingers bind methylated histones;

RING – a specialised type of zinc finger often found in ubiquitin protein ligases;

EnvC – bacterial protein which activates cell wall hydrolases and is required for daughter cell separation following cell division;

SNF2 – domain found in proteins involved in a variety of processes including transcription regulation, DNA repair, DNA recombination and chromatin unwinding;

NAT – N-acetyltransferase;

SNc - Staphylococcal nuclease fold;

Tudor – domain which can recognise methylated histone lysines and arginines; present in several RNA-binding proteins;

Chromo (chromatin organisation modifier) – domain which binds methylated histones; involved in chromatin organisation, specifically heterochromatin formation;

PWWP – domain which contains a Pro-Trp-Trp-Pro motif; binds methylated histone lysines; found in DNA-binding proteins that function as transcription factors;

Med26/TFIIS - TFIIS helical bundle-like domain; component of the mediator complex involved in the regulation of RNAPII-transcribed genes;

Znf-CW – a zinc finger domain containing conserved Cys and Trp residues; implicated in DNA binding and protein-protein interactions, particularly recognition of methylated histones;

ICP4 – Herpesvirus protein required for transcription of viral genes; binds DNA in a sequence-specific manner;

NuA4 – histone acetyltransferase subunit;

MOZ/SAS - suggested to be homologous to acetyltransferases;

ELP3 - radical SAM enzyme/protein acetyltransferase; this family includes elongator complex protein 3 (ELP3) which is a component of the RNAPII holoenzyme;

RimI – ribosomal protein acetyltransferase found in bacteria; mediates acetylation of N-terminal residues;

LPLAT - lysophospholipid acyltransferase;
AT_C – domain found at the C-terminus of several acyltransferases;
SET - Su(var)3-9, Enhancer-of-zeste, Trithorax; catalytic domain of lysine methyltransferases;
Znf-MYND - MYND-type zinc finger; protein-protein interaction domain;
TOM70 – component of the translocase of outer membrane (TOM) complex involved in mitochondrial import;
D-ala D-ala ligase – bacterial enzyme involved in peptidoglycan synthesis and cell wall biogenesis;
DOT - Disruptor of telomeric silencing; domain which regulates gene expression via histone methylation;
Class I HDACs - Zn-dependent histone deacetylases;
Class II HDACs- Zn-dependent histone deacetylases;
Sir2 HDACs – sirtuins; NAD-dependent histone deacetylases;
JmjC – found in metalloenzymes that adopt the cupin fold; function in histone demethylation
LCM - leucine carboxyl methyltransferase;
Kelch – sequence motif present in proteins with diverse functions including cytoskeletal support and oxidation;
SMC (structural maintenance of chromosomes) – chromosome segregation protein;
Patched – transmembrane receptor for Sonic Hedgehog;
TBP (TATA-binding protein) – part of the DNA-binding transcription factor complex TFIID;

B. Multiple sequence alignment of the putative TbCRD1 chromodomain with *S. pombe* Chp2 and Swi6; Human Cbx5 and Mouse Cbx7. Level of sequence similarity is indicated as a color gradient from cyan (low similarity) to red (high similarity).
C. Multiple sequence alignments of the TbBDF6 Bromo domain with indicated protein accession numbers including Human BRD7, TIF1A and BPTF. Level of sequence similarity is indicated as a color gradient from cyan (low similarity) to red (high similarity).

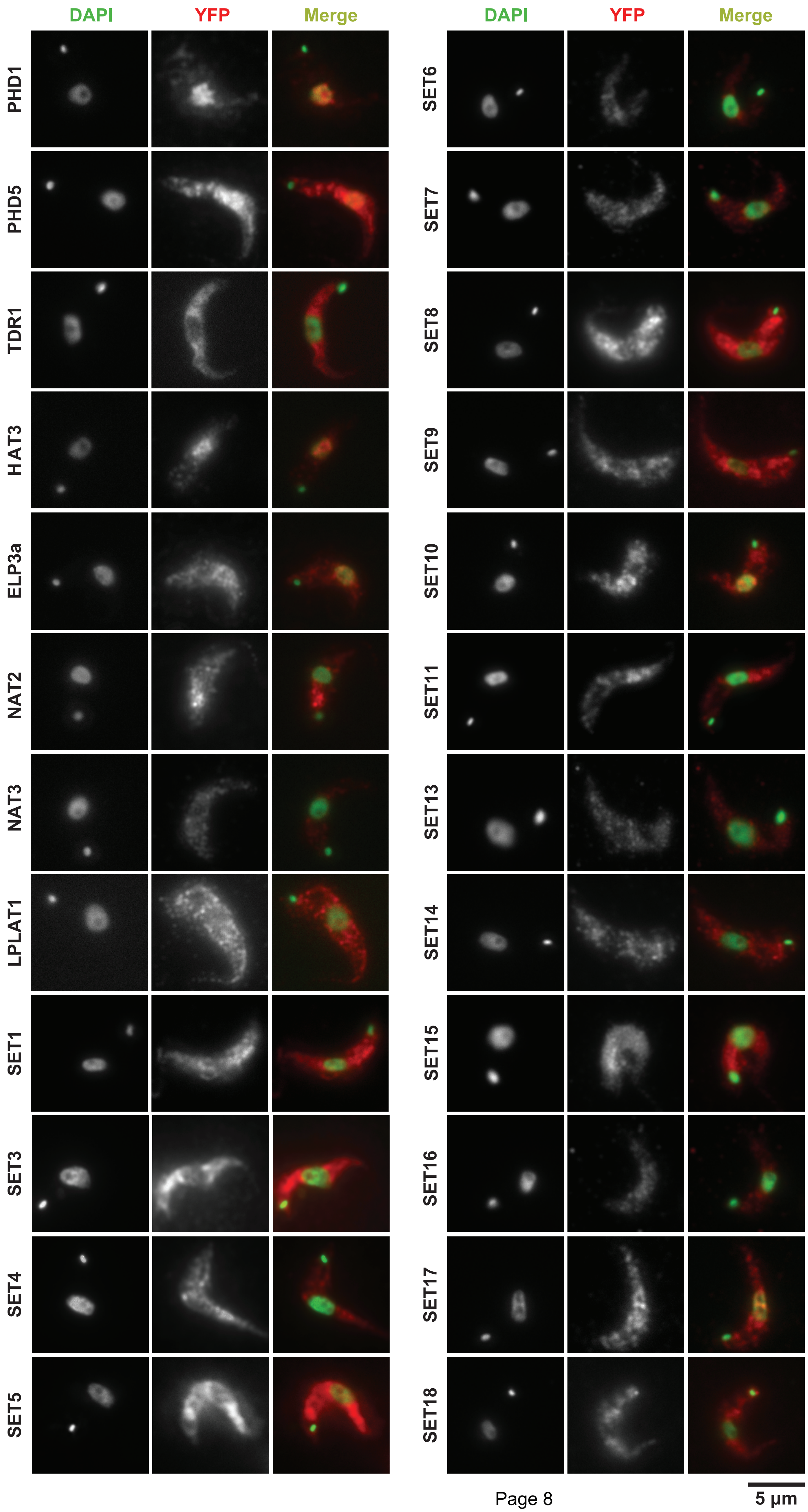

Supplemental Fig. S2 part 1

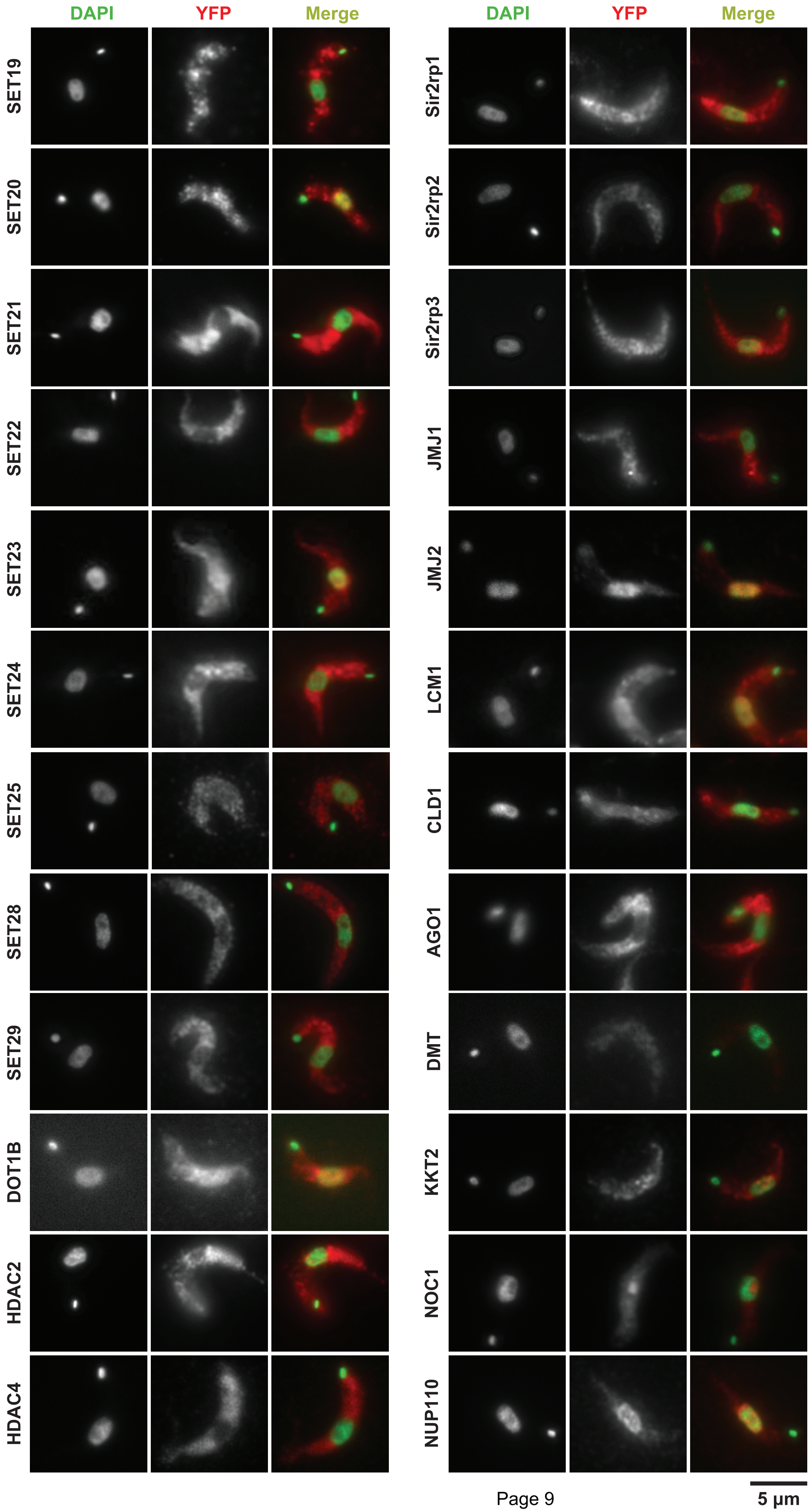

Supplemental Fig. S2 part 2

Supplemental Fig. S2. Cellular localization of *T. brucei* proteins with no specific genomic enrichment by ChIP-seq.

The indicated YFP-tagged proteins expressed in bloodstream Lister 427 cells from their endogenous genomic loci were detected with an anti-GFP primary antibody and an Alexa Fluor 568 labelled secondary antibody (red). Nuclear and kinetoplast (mitochondrial) DNA were stained with DAPI (green). Representative images are shown for each candidate protein for which no specific ChIP-seq pattern was detected. Images of KKT2, NOC1 and NUP110 are included as controls for nuclear localization patterns. AGO1 serves as a control for cytoplasmic localization. Scale bar = 5 μm.

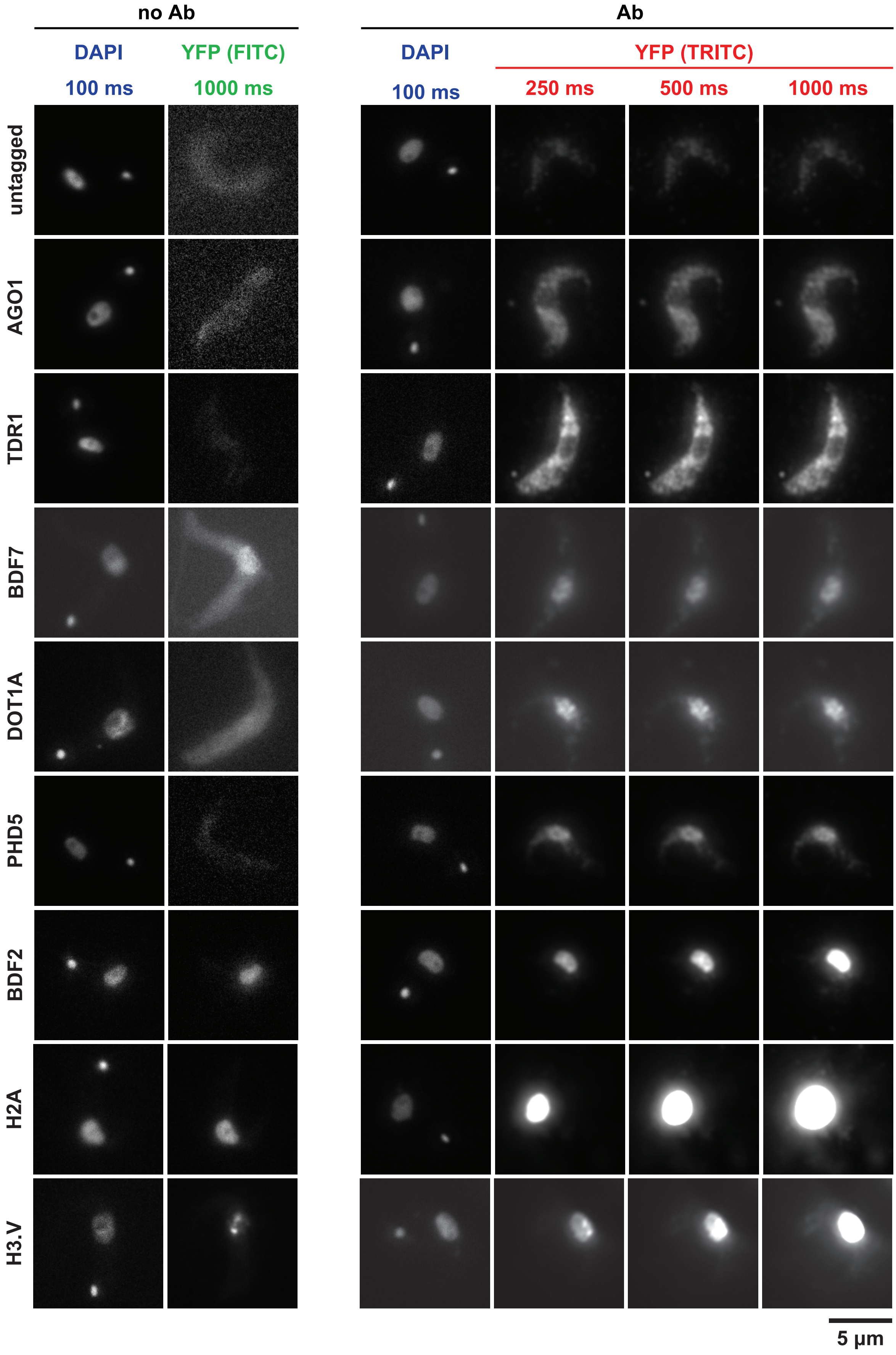

no Ab
Ab
DAPI
YFP (FITC)
100 ms
1000 ms
DAPI
YFP (TRITC)
100 ms
250 ms
500 ms
1000 ms
untagged
AGO1
TDR1
BDF7
DOT1A
PHD5
BDF2
H2A
H3.V
5 µm

Supplemental Fig. S3. Comparison of different imaging procedures.

Selected YFP-tagged proteins and the untagged control were imaged with and without antibody staining. Cells stained only with DAPI (no Ab) were imaged at 100 ms (DAPI channel; blue) and 1000 ms (FITC channel where YFP is detected; green). Cells stained with DAPI, primary and secondary antibody (Ab) were imaged at 100 ms (DAPI channel) and 250, 500 or 1000 ms (TRITC channel where antibody-labelled YFP-tagged proteins are detected; red). AGO1 is used as a control for cytoplasmic localization whereas histones H2A and H3.V are controls for nuclear enrichment. Note that in some cases the inherent fluorescence of the expressed YFP fusion protein generates a signal even in the absence of secondary antibody. Scale bar = 5 μm.

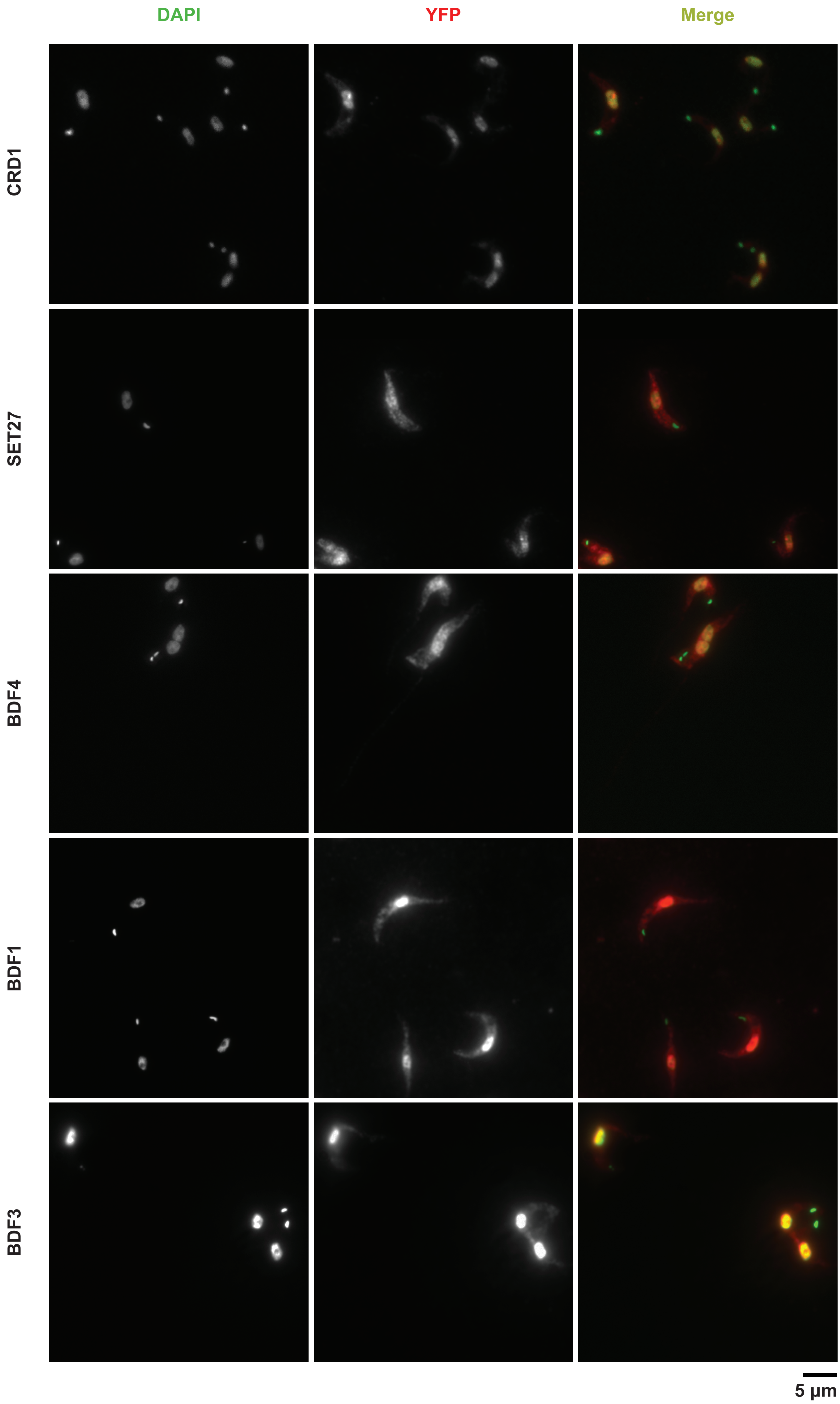

Supplemental Fig. S4 part 1

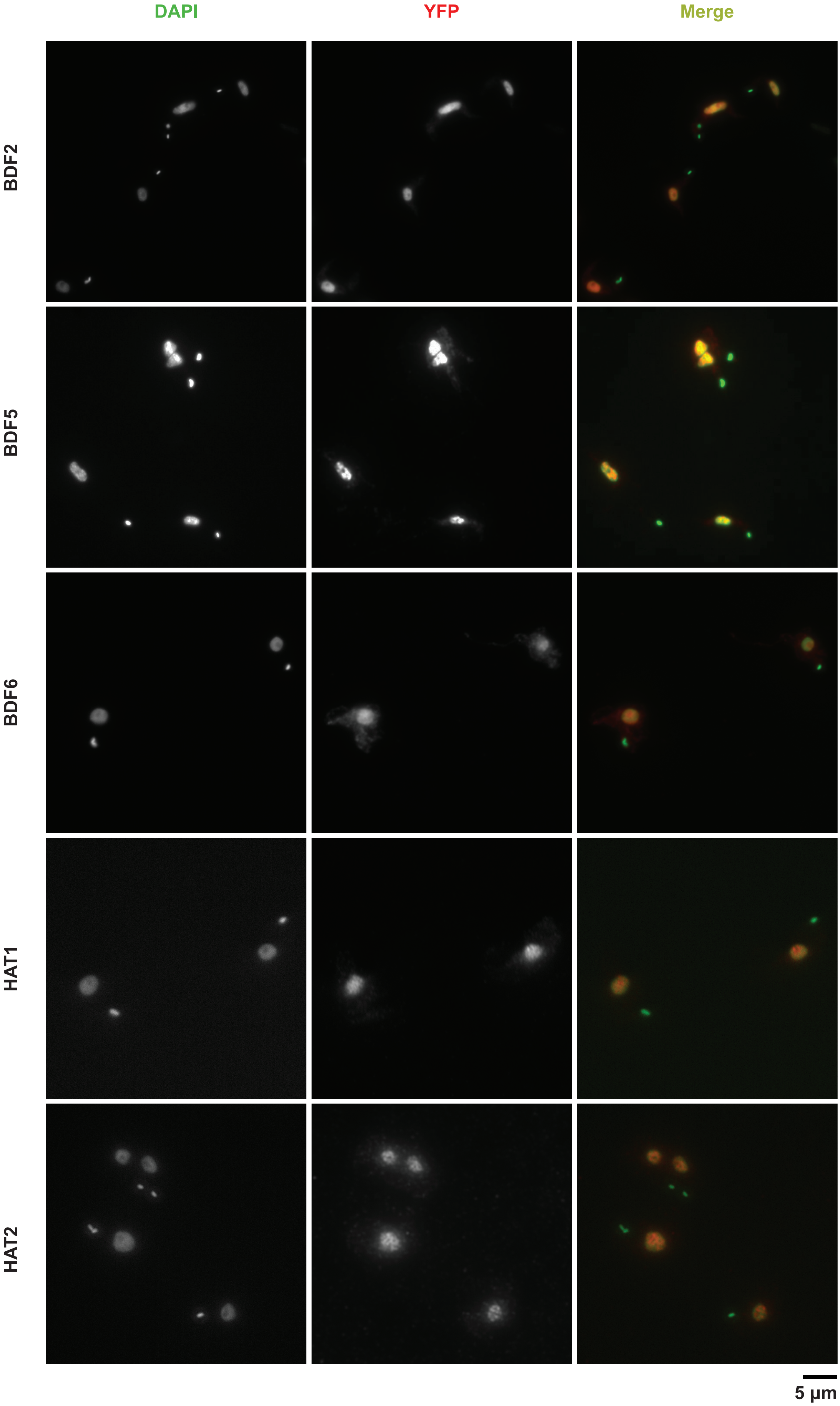
DAPI
YFP
Merge
BDF2
BDF5
BDF6
HAT1
HAT2
5 µm

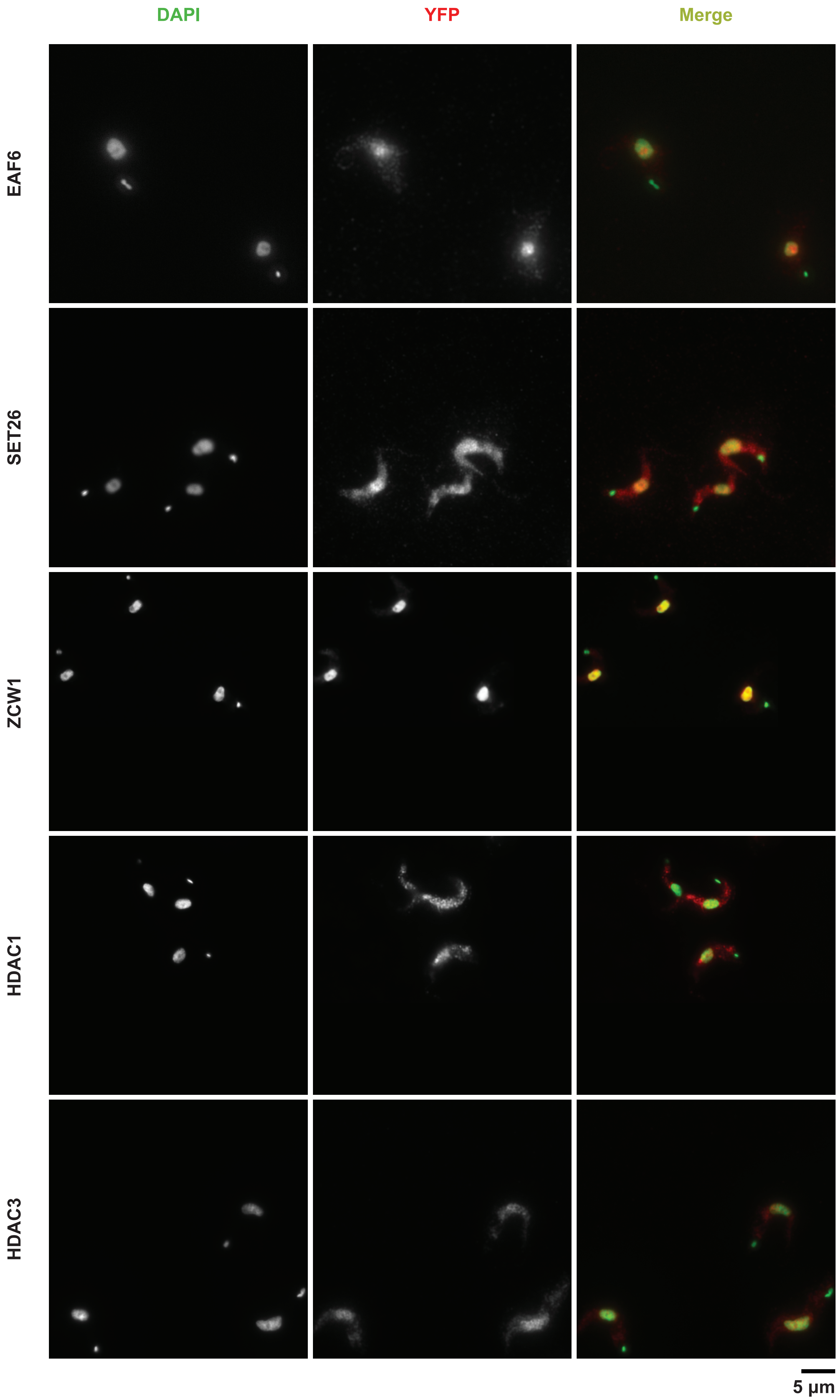

Supplemental Fig. S4 part 3

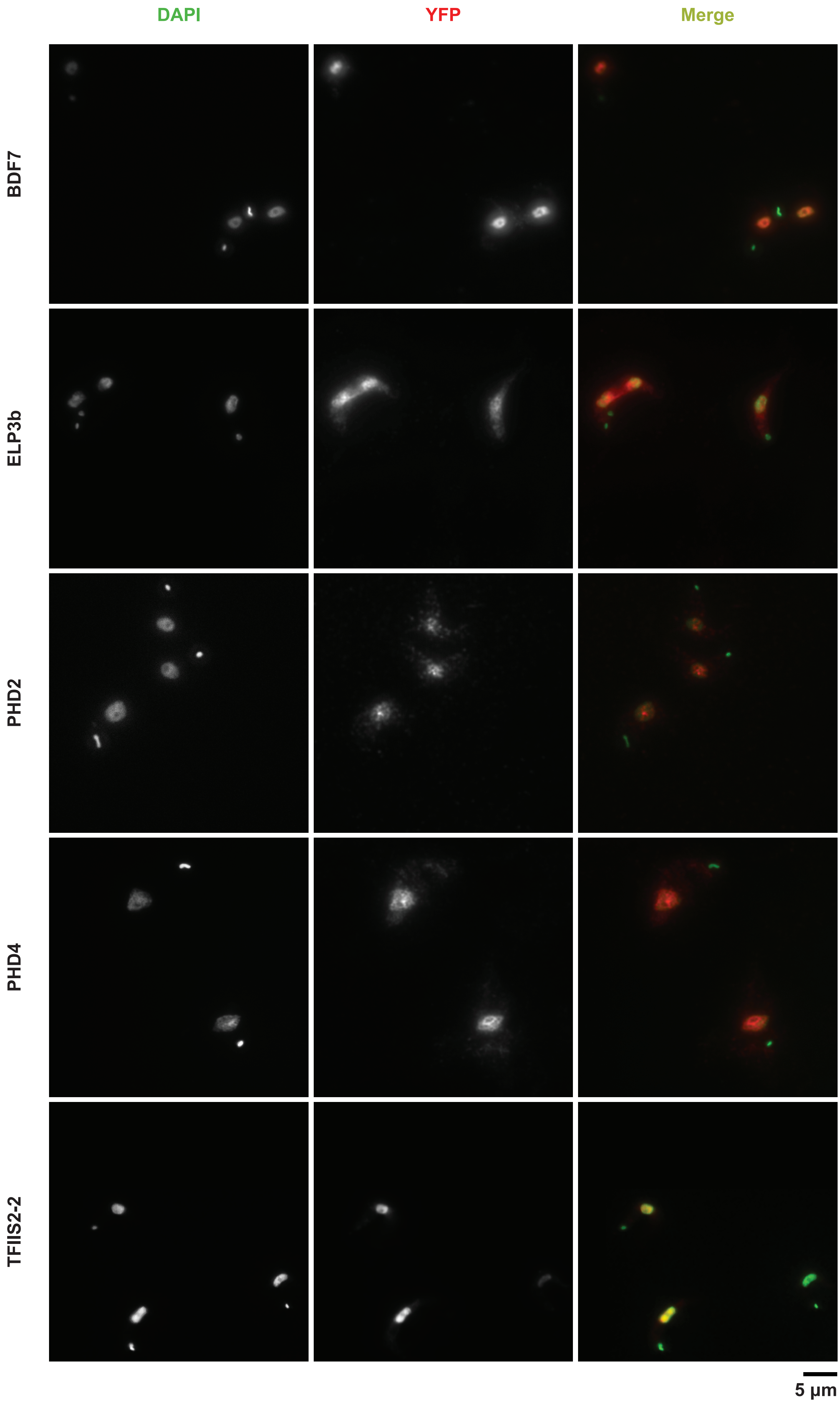

DAPI
YFP
Merge
BDF7
ELP3b
PHD2
PHD4
TFIIS2-2
5 µm

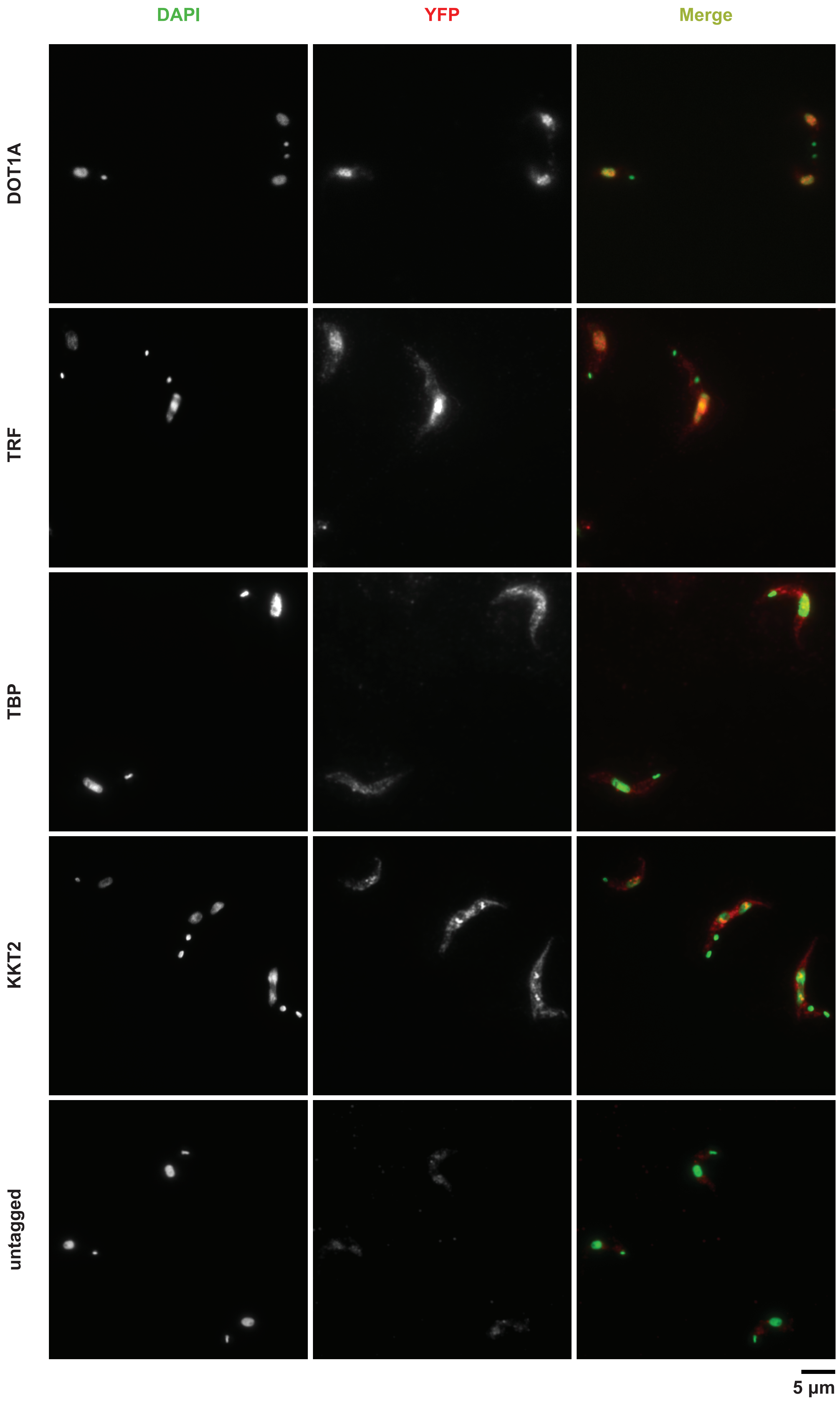

DAPI
YFP
Merge
DOT1A
TRF
TBP
KKT2
untagged
5 µm

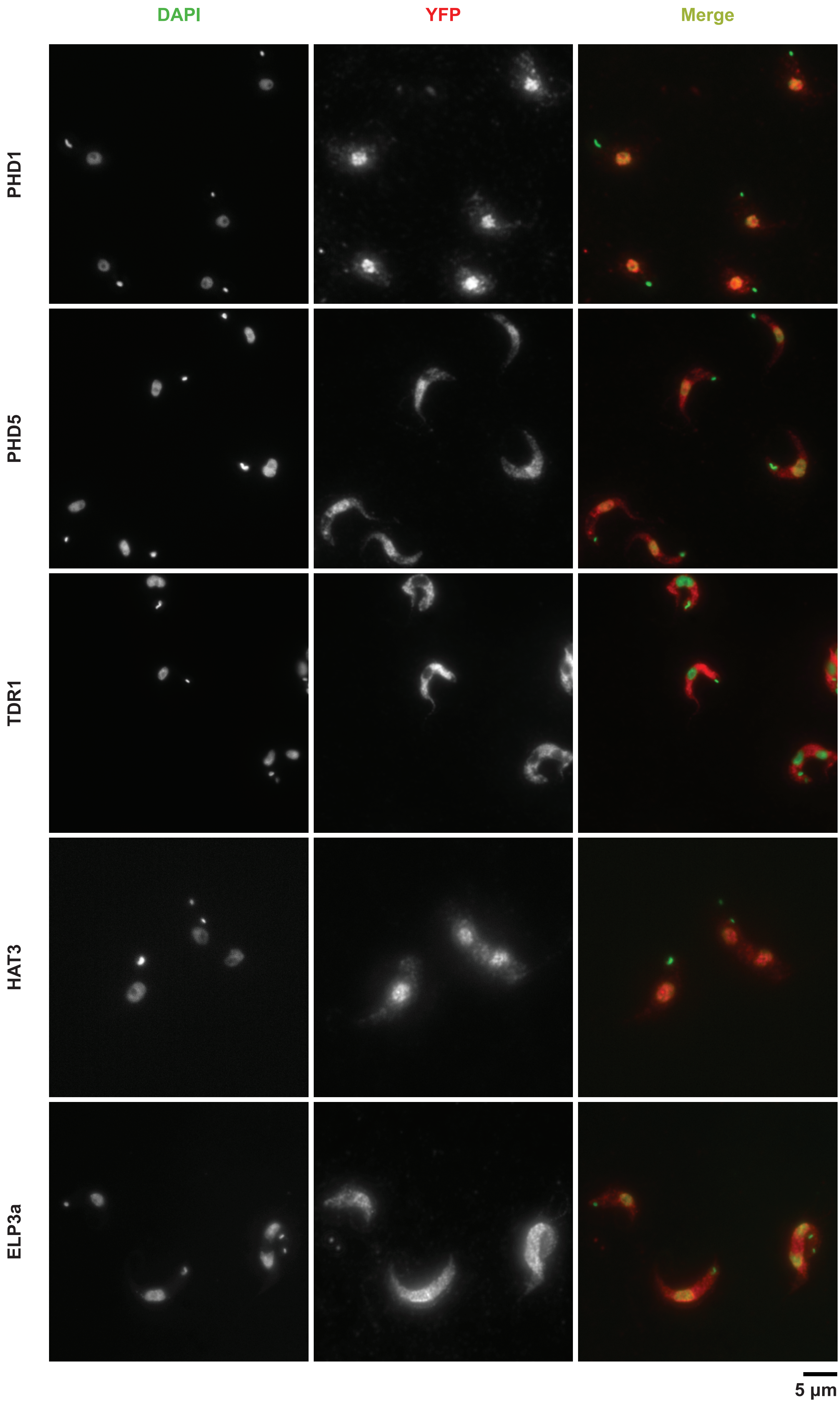

Supplemental Fig. S4 part 6

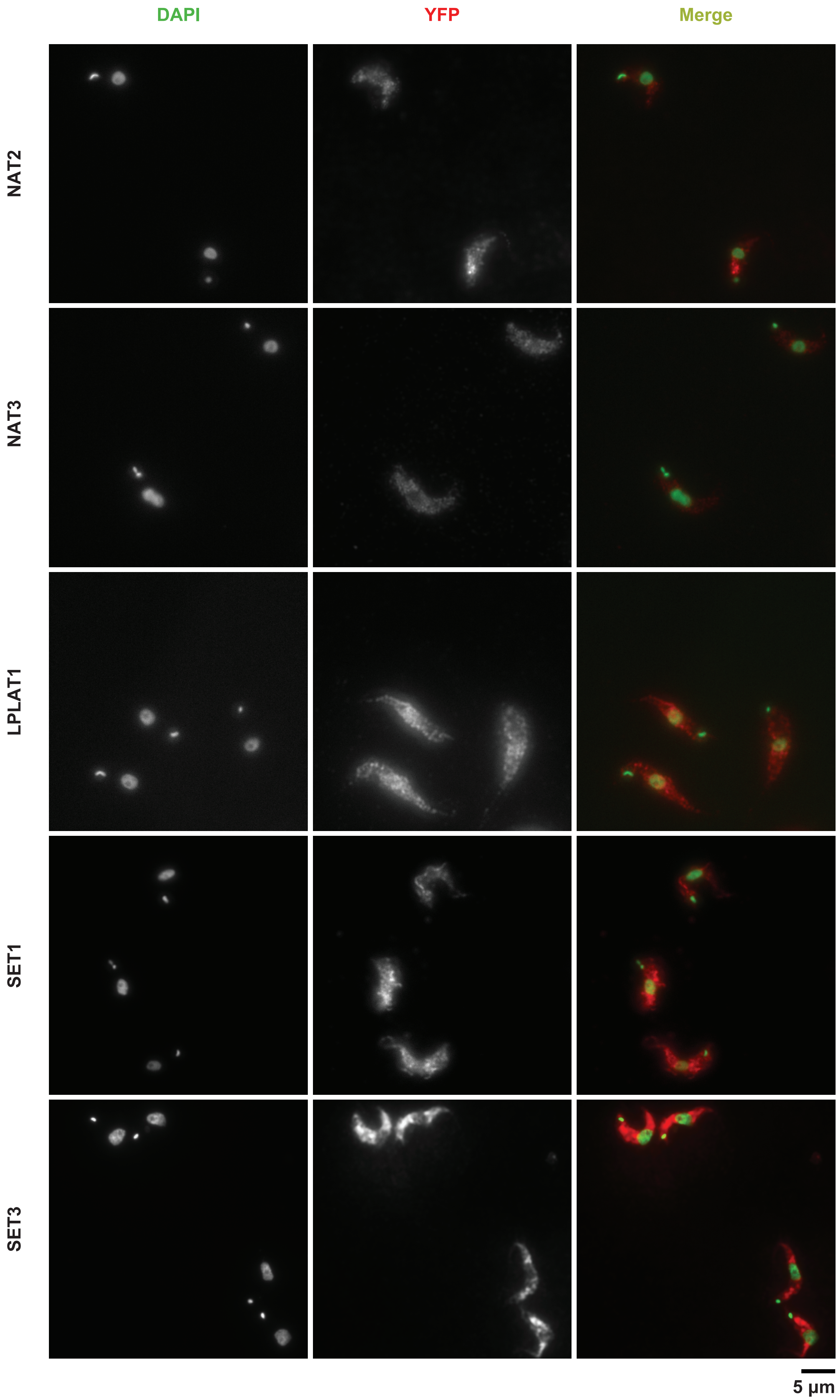
DAPI
YFP
Merge
NAT2
NAT3
LPLAT1
SET1
SET3
5 µm

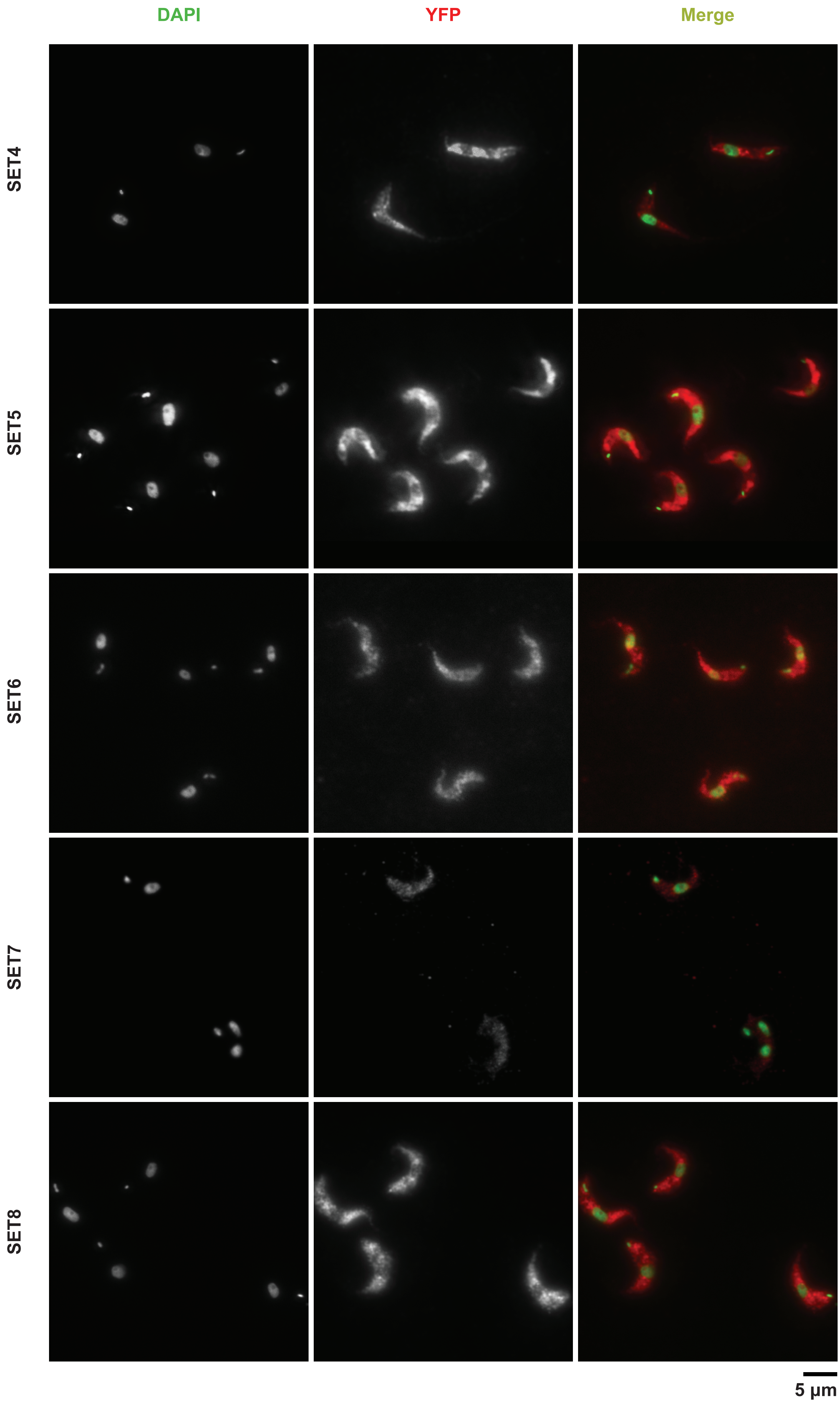

DAPI
YFP
Merge
SET4
SET5
SET6
SET7
SET8
5 µm

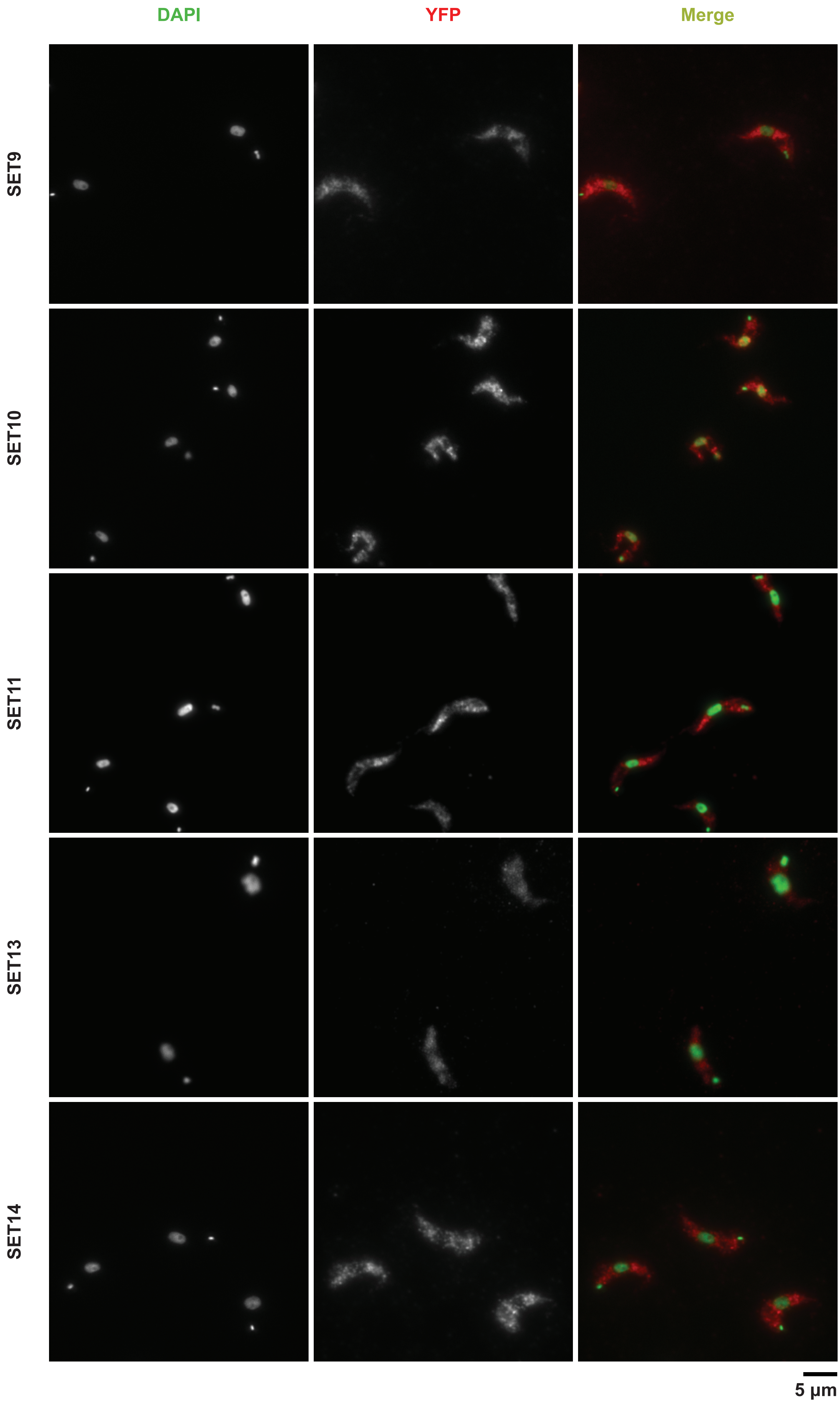
DAPI
YFP
Merge
SET9
SET10
SET11
SET13
SET14
5 µm

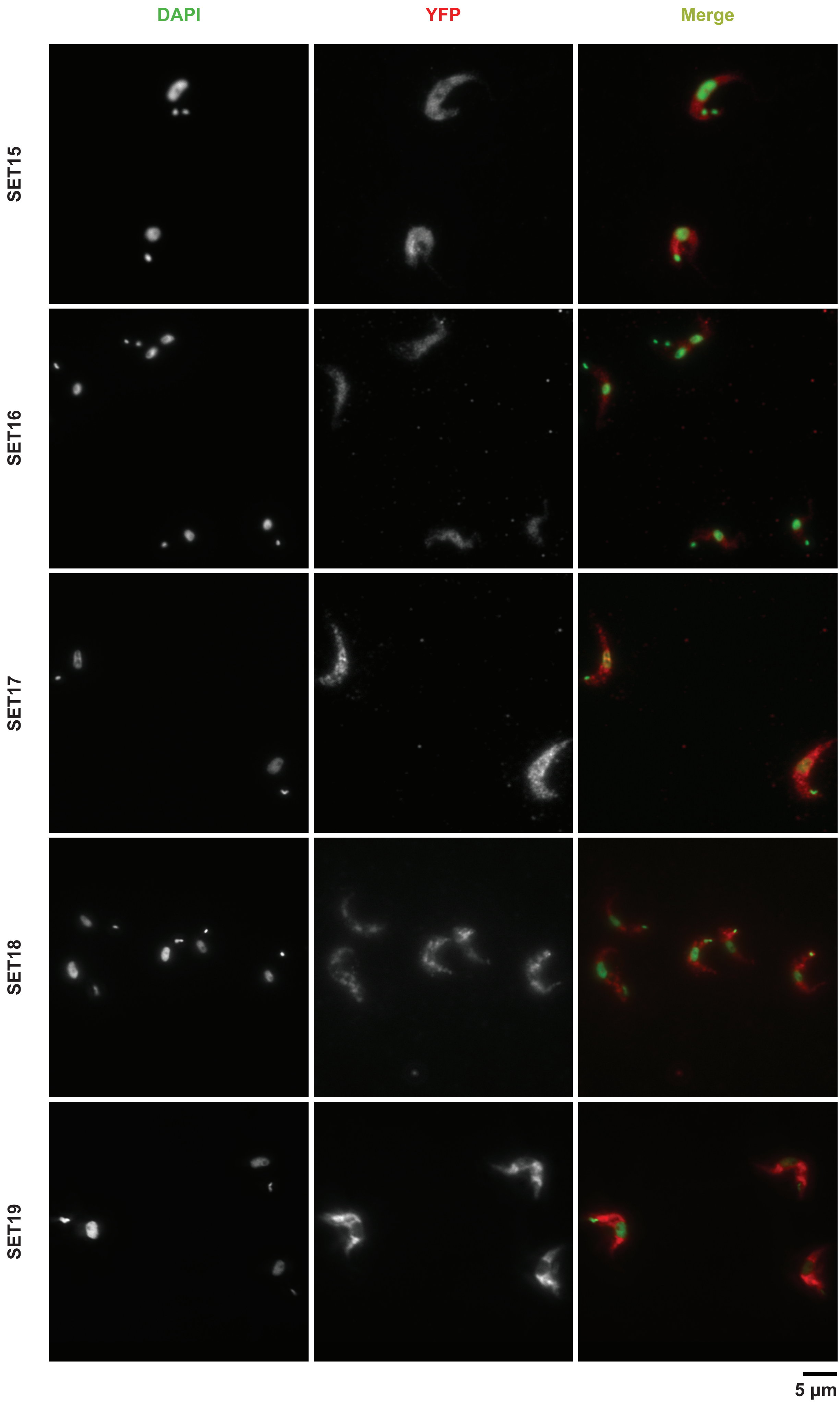

DAPI
YFP
Merge
SET15
SET16
SET17
SET18
SET19
5 µm

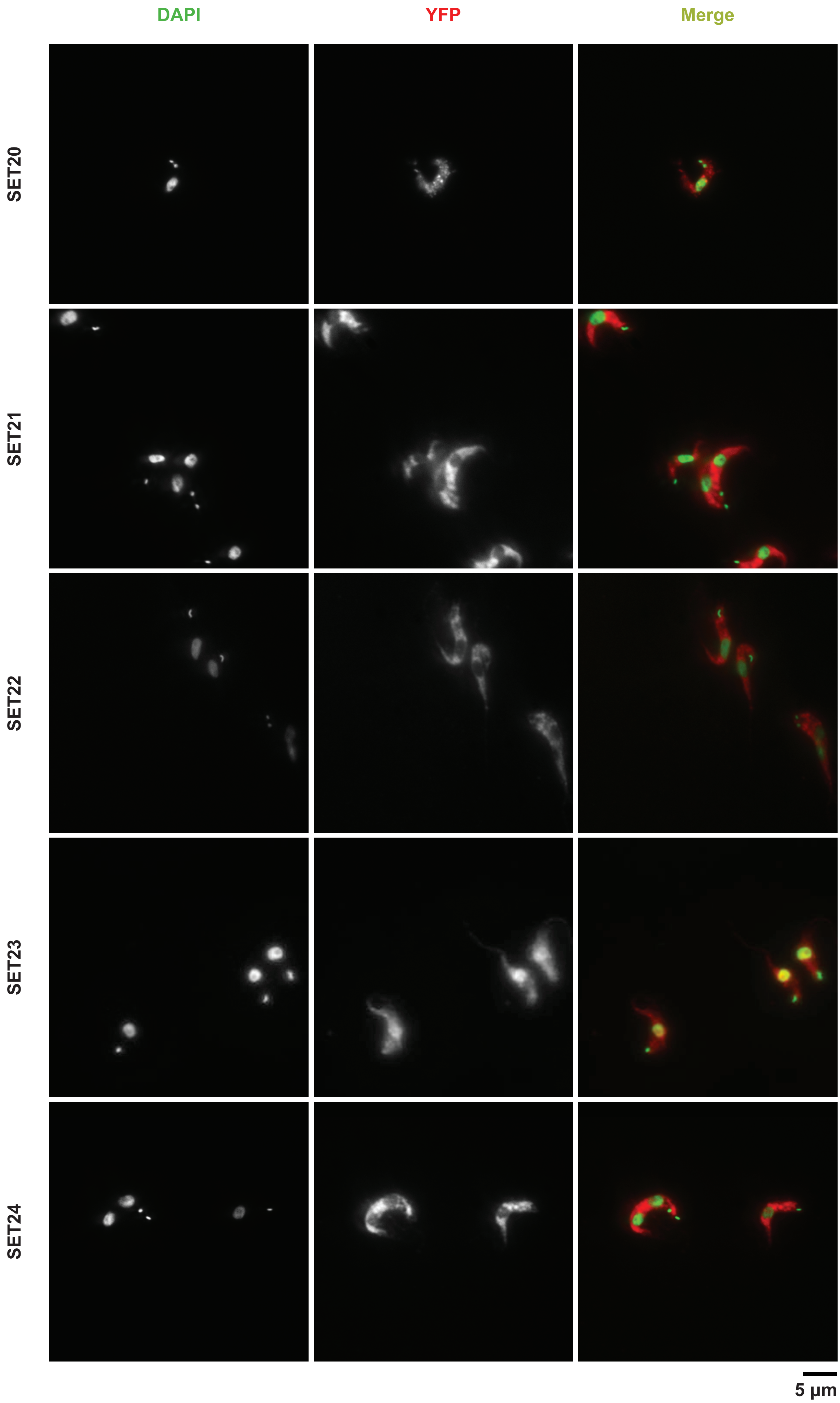

Supplemental Fig. S4 part 11

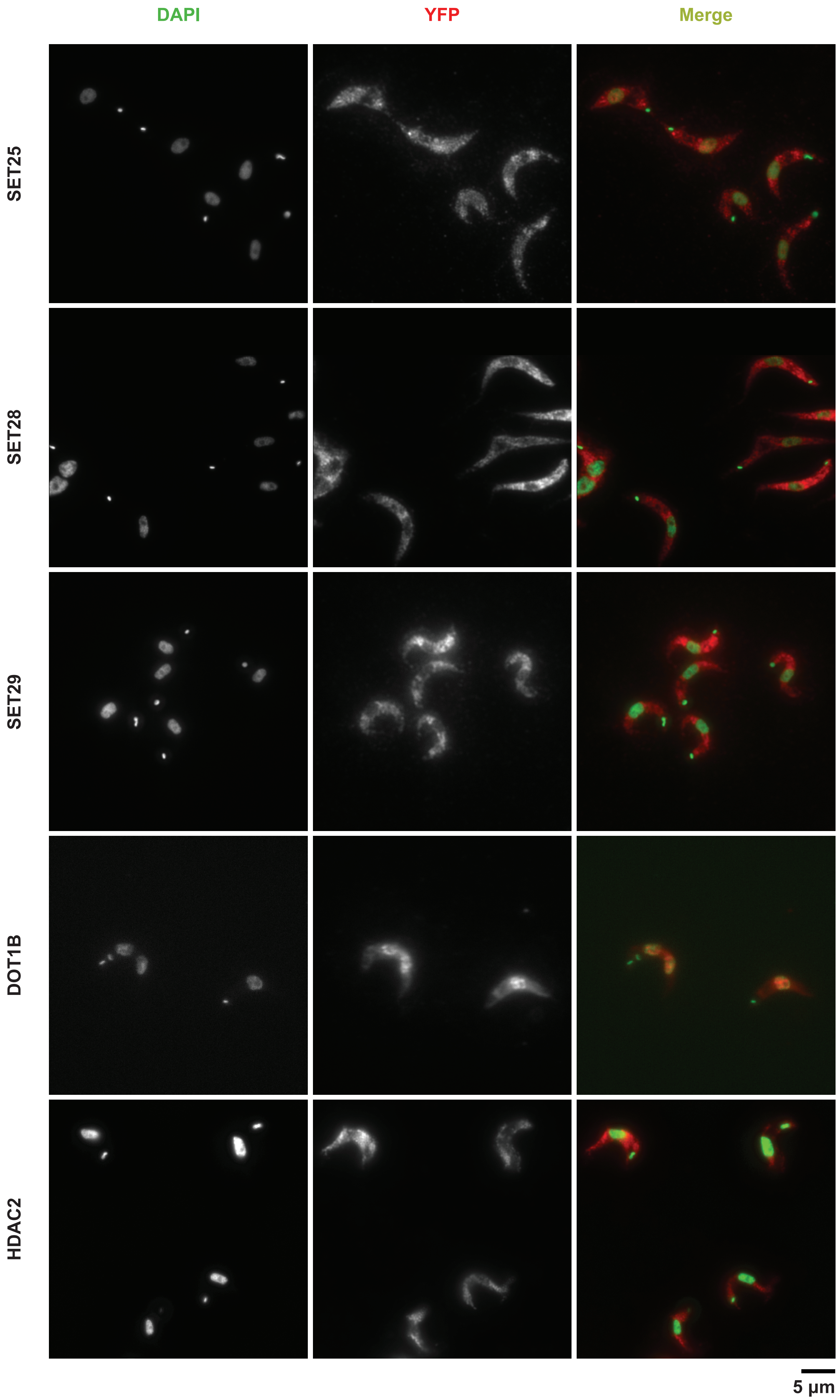

Supplemental Fig. S4 part 12

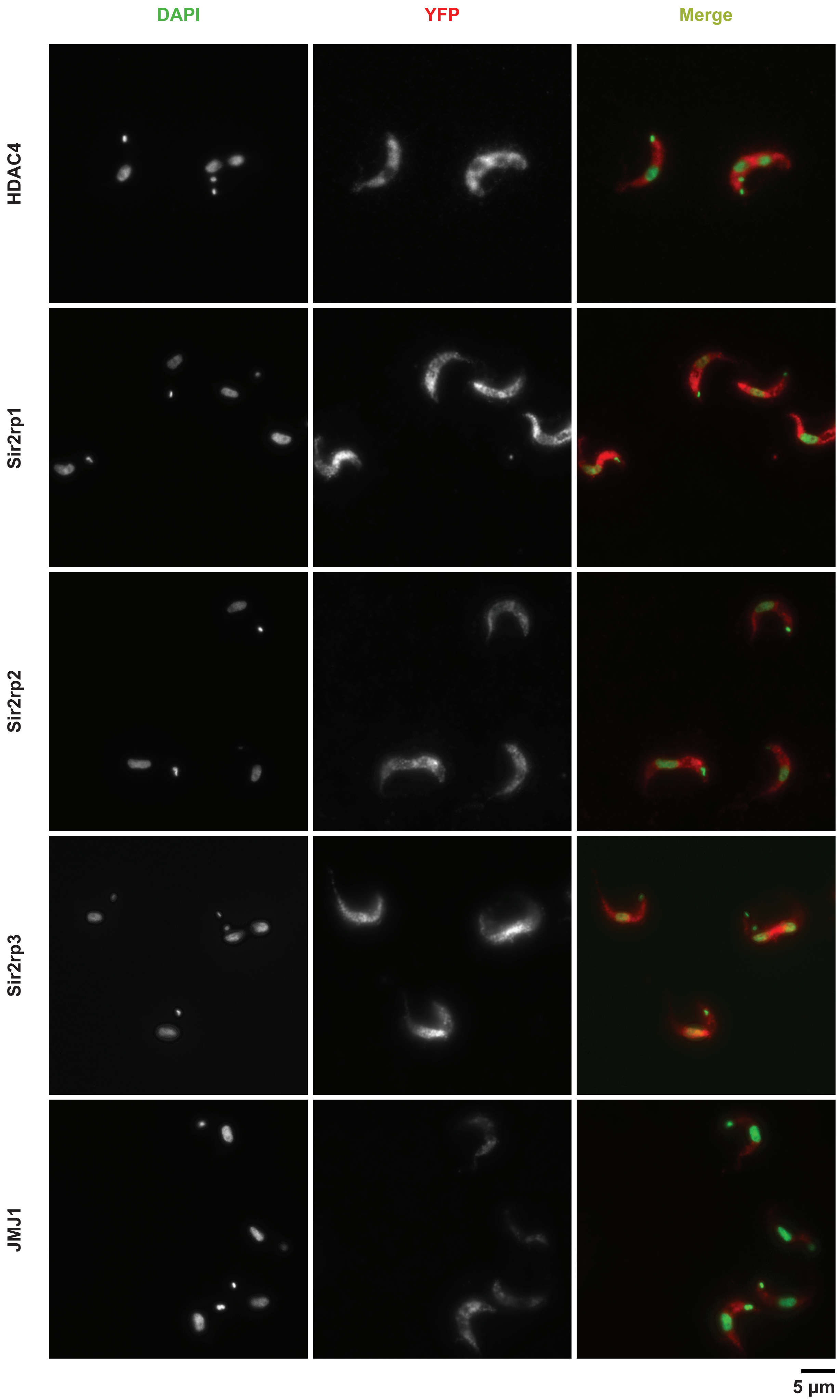
DAPI
YFP
Merge
HDAC4
Sir2rp1
Sir2rp2
Sir2rp3
JMJ1
5 µm

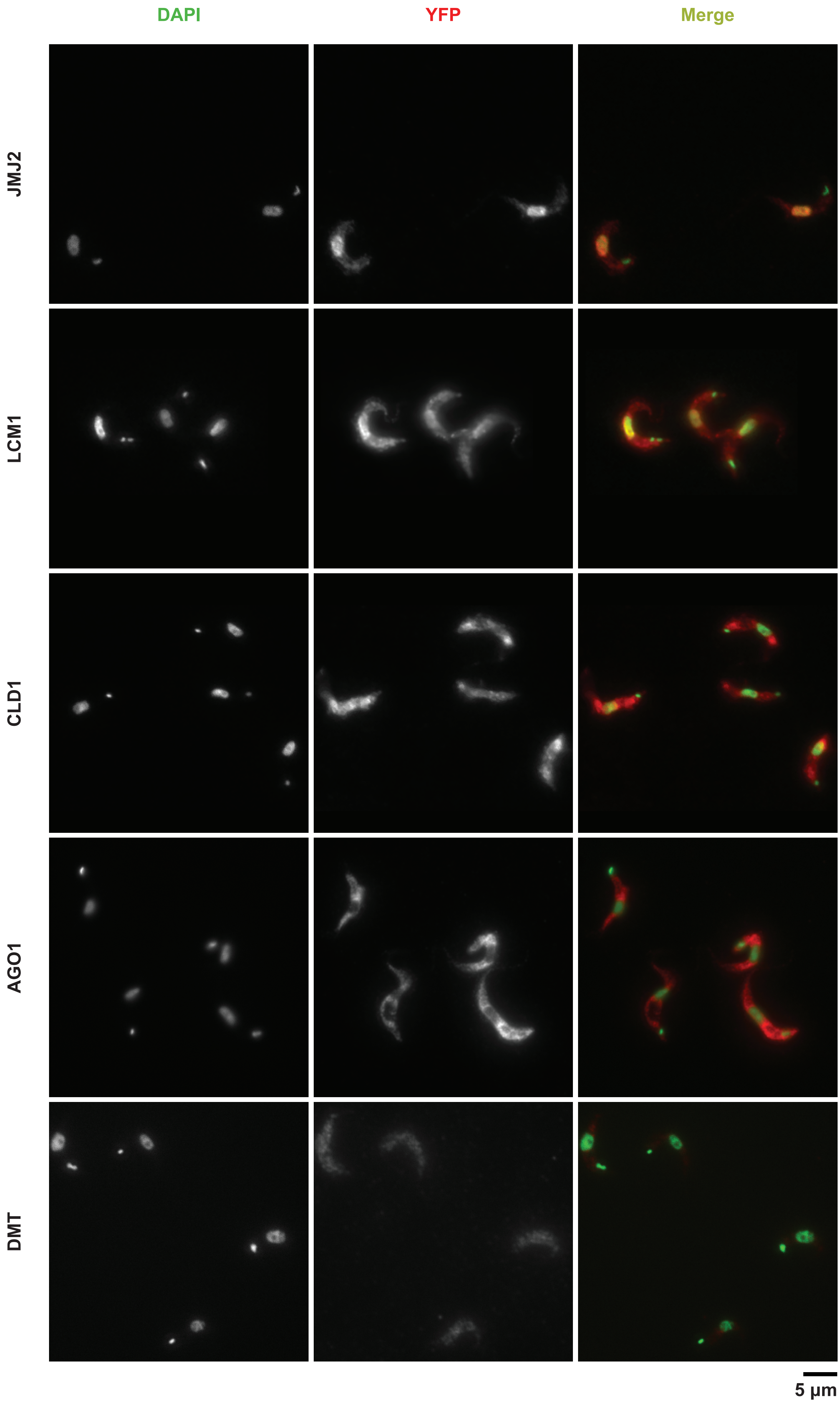

Supplemental Fig. S4 part 14

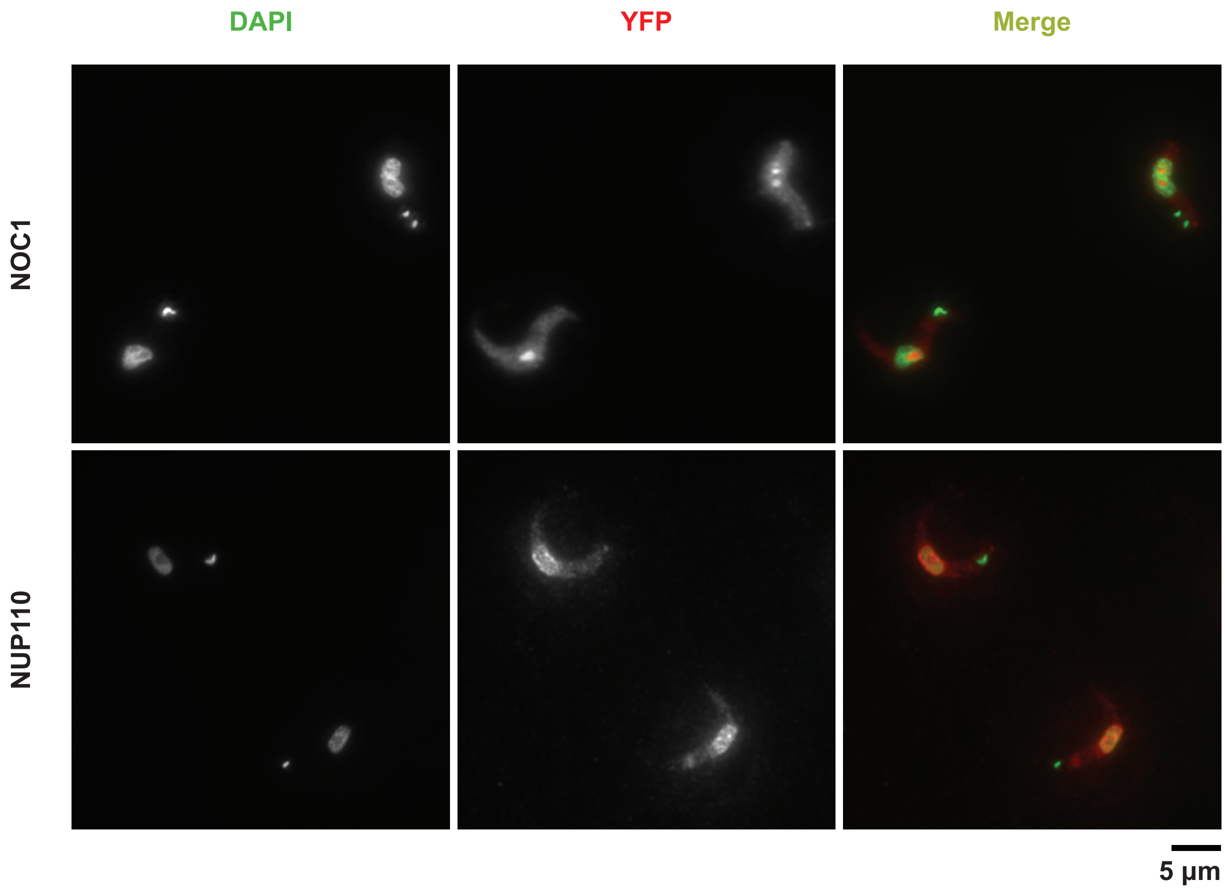

Supplemental Fig. S4 part 15

Supplemental Fig. S4. Localization of YFP-tagged proteins at different stages of the cell cycle.

The indicated YFP-tagged proteins expressed in bloodstream Lister 427 cells from their endogenous genomic loci were detected with an anti-GFP primary antibody and an Alexa Fluor 568 labelled secondary antibody (red). Nuclear and kinetoplast (mitochondrial) DNA were stained with DAPI (green). Scale bar = 5 μm.

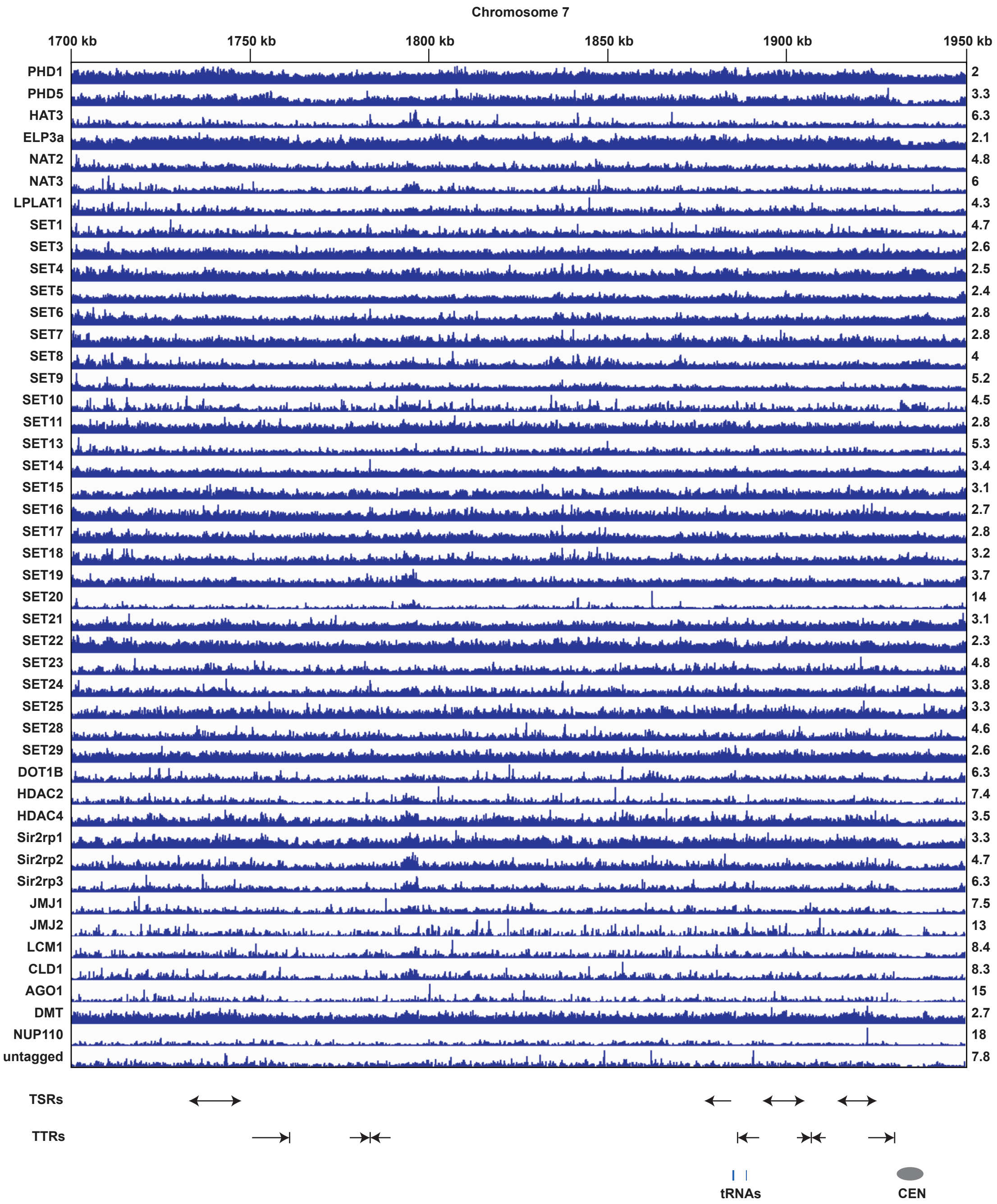
Chromosome 7
1700 kb
1750 kb
1800 kb
1850 kb
1900 kb
1950 kb
PHD1 2
PHD5 3.3
HAT3 6.3
ELP3a 2.1
NAT2 4.8
NAT3 6
LPLAT1 4.3
SET1 4.7
SET3 2.6
SET4 2.5
SET5 2.4
SET6 2.8
SET7 2.8
SET8 4
SET9 5.2
SET10 4.5
SET11 2.8
SET13 5.3
SET14 3.4
SET15 3.1
SET16 2.7
SET17 2.8
SET18 3.2
SET19 3.7
SET20 14
SET21 3.1
SET22 2.3
SET23 4.8
SET24 3.8
SET25 3.3
SET28 4.6
SET29 2.6
DOT1B 6.3
HDAC2 7.4
HDAC4 3.5
Sir2rp1 3.3
Sir2rp2 4.7
Sir2rp3 6.3
JMJ1 7.5
JMJ2 13
LCM1 8.4
CLD1 8.3
AGO1 15
DMT 2.7
NUP110 18
untagged 7.8
TSRs
TTRs
tRNAs
CEN

Supplemental Fig. S5. YFP-tagged proteins with no specific genomic enrichment by ChIP-seq.

Tracks show ChIP-seq reads of the indicated proteins for the region of Chromosome 7 shown in Fig. 2A and are scaled separately as reads per million (values shown at the end of each track). One replicate is included for each protein.

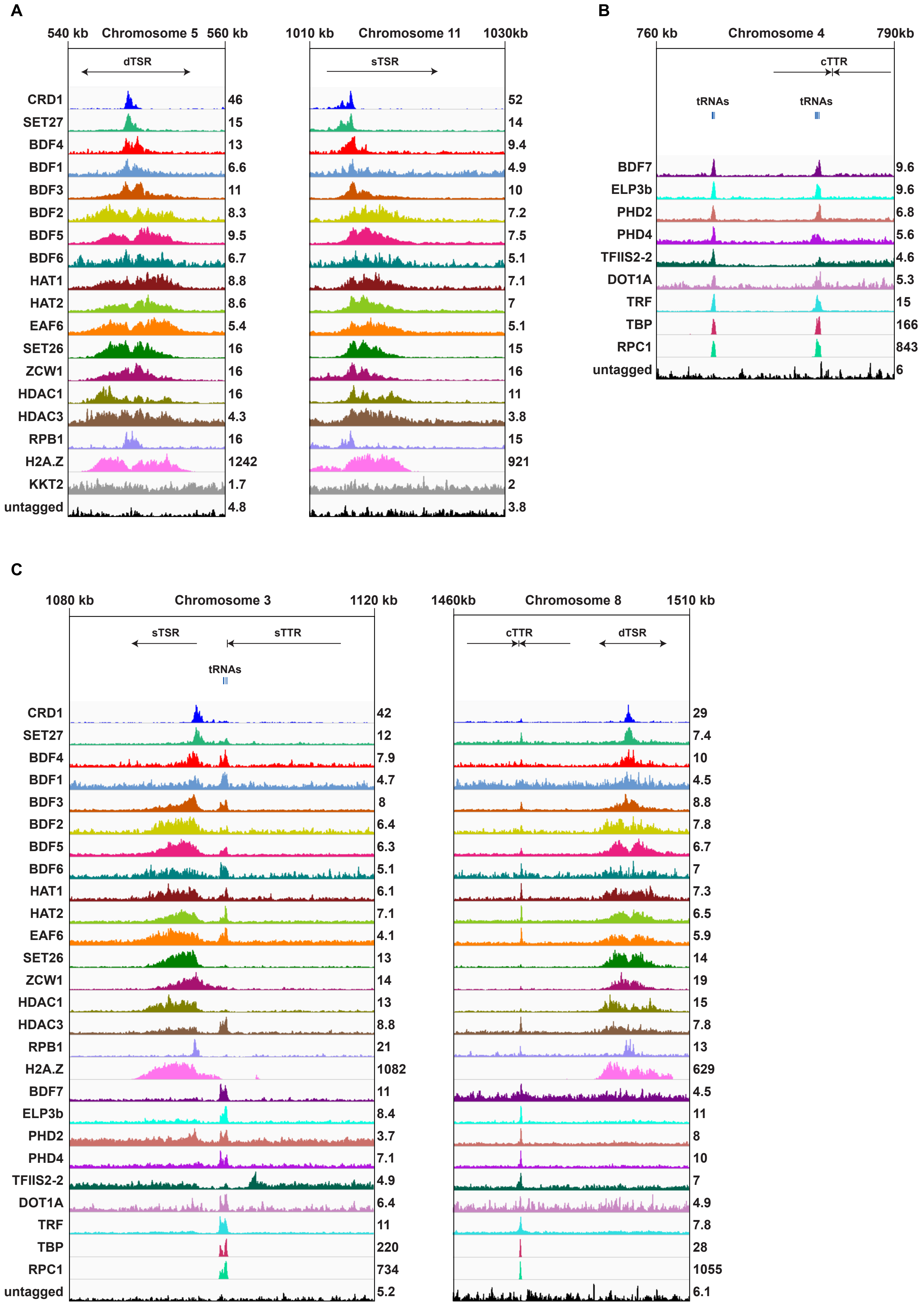
A
540 kb
Chromosome 5
560 kb
dTSR
1010 kb
Chromosome 11
1030kb
sTSR
CRD1
SET27
BDF4
BDF1
BDF3
BDF2
BDF5
BDF6
HAT1
HAT2
EAF6
SET26
ZCW1
HDAC1
HDAC3
RPB1
H2A.Z
KKT2
untagged
46
15
13
6.6
11
8.3
9.5
6.7
8.8
8.6
5.4
16
16
16
4.3
16
1242
1.7
4.8
52
14
9.4
4.9
10
7.2
7.5
5.1
7.1
7
5.1
15
16
11
3.8
15
921
2
3.8
B
760 kb
Chromosome 4
790kb
cTTR
tRNAs
tRNAs
BDF7
ELP3b
PHD2
PHD4
TFIIS2-2
DOT1A
TRF
TBP
RPC1
untagged
9.6
9.6
6.8
5.6
4.6
5.3
15
166
843
6
C
1080 kb
Chromosome 3
1120 kb
sTSR
sTTR
tRNAs
1460kb
Chromosome 8
1510 kb
cTTR
dTSR
CRD1
SET27
BDF4
BDF1
BDF3
BDF2
BDF5
BDF6
HAT1
HAT2
EAF6
SET26
ZCW1
HDAC1
HDAC3
RPB1
H2A.Z
BDF7
ELP3b
PHD2
PHD4
TFIIS2-2
DOT1A
TRF
TBP
RPC1
untagged
42
12
7.9
4.7
8
6.4
6.3
5.1
6.1
7.1
4.1
13
14
13
8.8
21
1082
11
8.4
3.7
7.1
4.9
6.4
11
220
734
5.2
29
7.4
10
4.5
8.8
7.8
6.7
7
7.3
6.5
5.9
14
19
15
7.8
13
629
4.5
11
8
10
7
4.9
7.8
28
1055
6.1

Supplemental Fig. S6. Examples of ChIP-seq enrichment of TSR- and RNAPIII/TTR-associated factors.

Tracks show one ChIP-seq replicate for each protein and are scaled separately as reads per million (values shown at the end of each track).

A. Left - enrichment at a bidirectional/divergent TSR.
Right - enrichment at a unidirectional/single TSR.

B. Enrichment at two tRNA clusters, one of which overlaps a convergent TTR.

C. Coincidence of peaks of the TSR- and RNAPIII/TTR-associated factors.

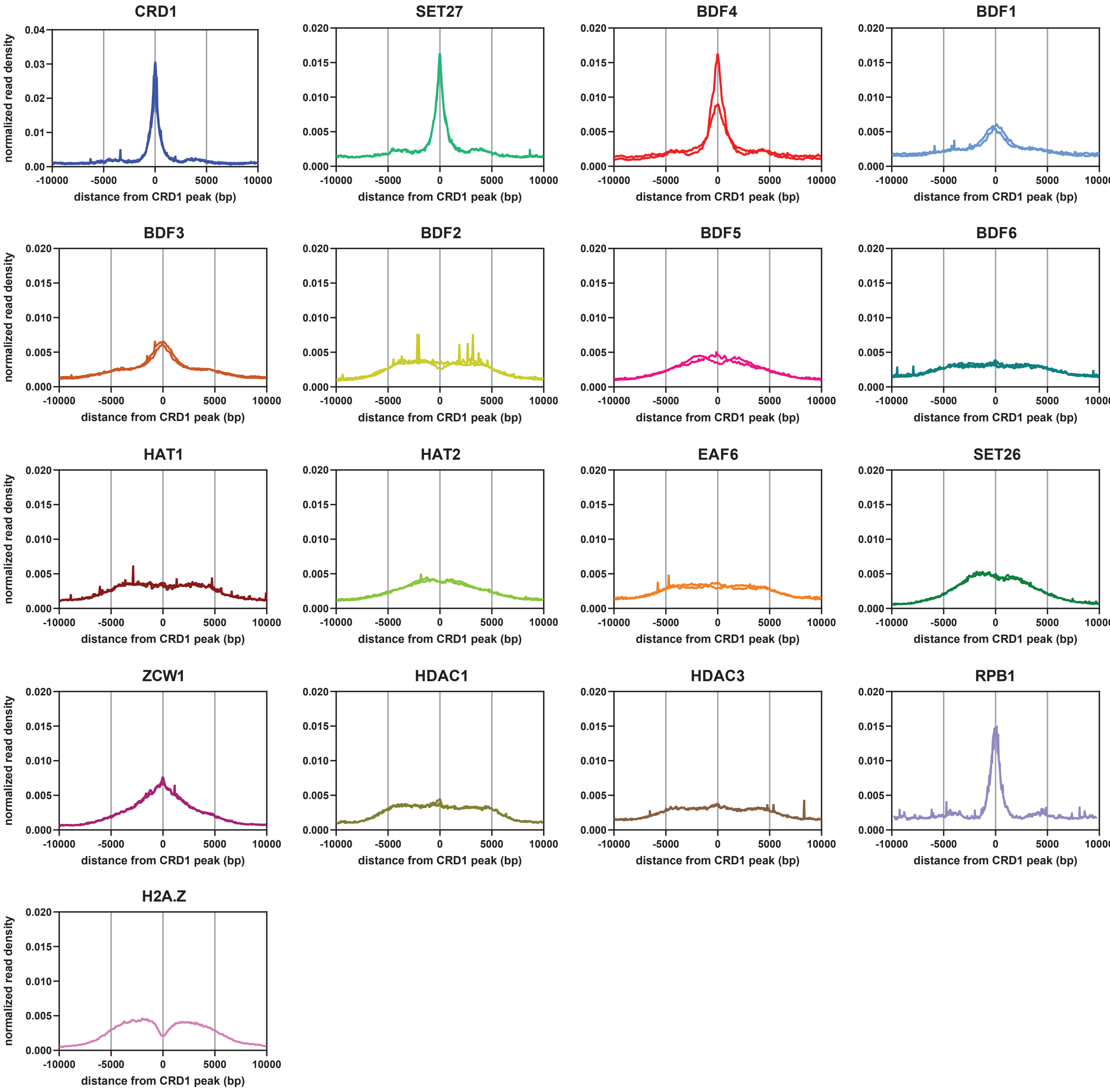
CRD1
SET27
BDF4
BDF1
BDF3
BDF2
BDF5
BDF6
HAT1
HAT2
EAF6
SET26
ZCW1
HDAC1
HDAC3
RPB1
H2A.Z
normalized read density
distance from CRD1 peak (bp)
0.00
0.01
0.02
0.03
0.04
0.000
0.005
0.010
0.015
0.020
-10000
-5000
0
5000
10000

Supplemental Fig. S7. Average metagene plots.

For each protein, normalized reads around the CRD1 peak summits were averaged and plotted as density. Plots show separately data from individual ChIP-seq replicates. Note the different scale for CRD1.

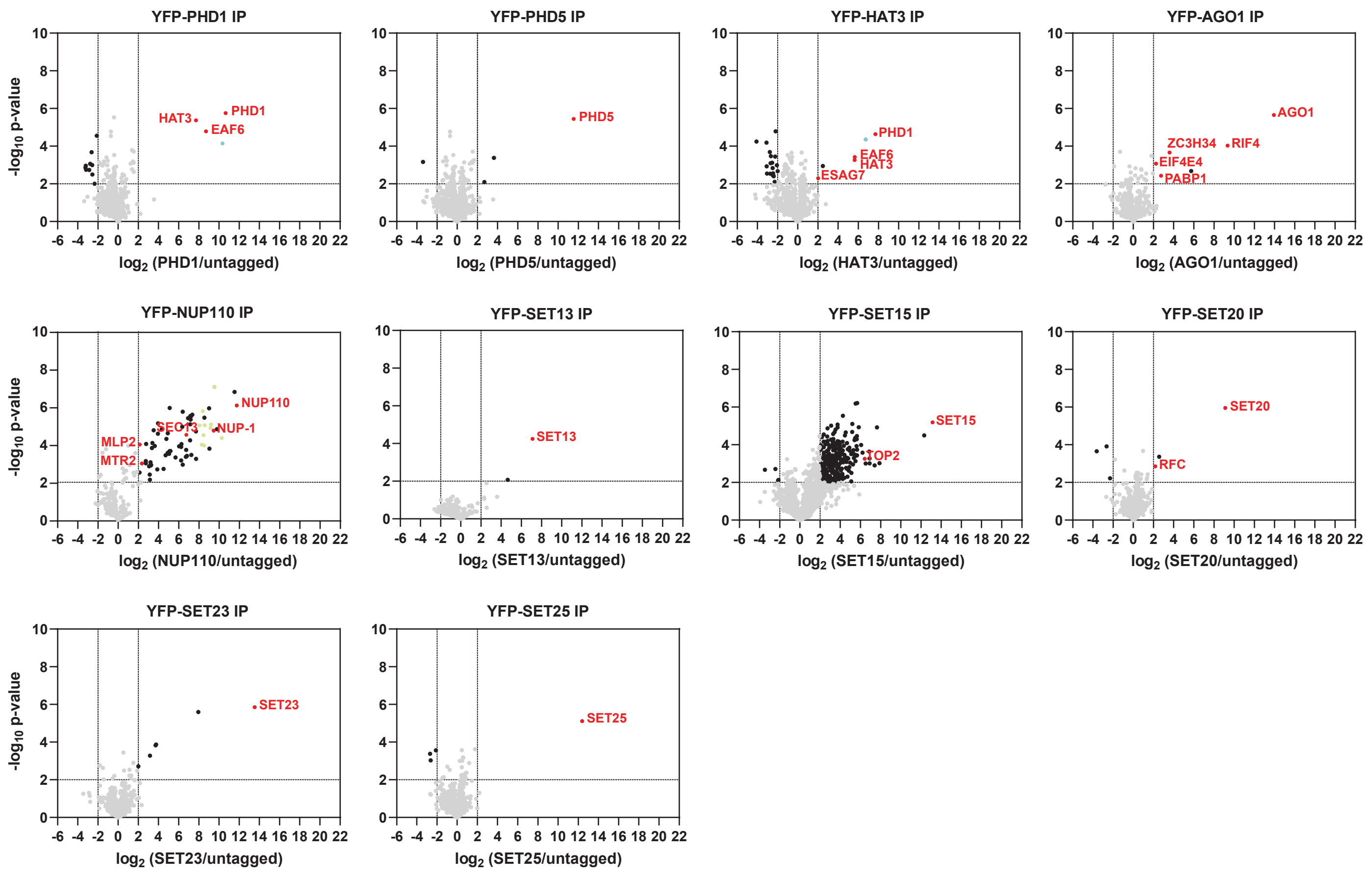

Supplemental Fig. S8

Supplemental Fig. S8. Proteomic analysis of affinity selections for several non-chromatin associated proteins.

Data for each plot is based on three biological replicates. Cut-offs used for significance: $\log_2$ (tagged/untagged) > 2 or < -2 and $p < 0.01$ (Student's *t*-test). Enrichment scores for proteins identified in each affinity selection are presented in Supplemental Table S4. Significantly enriched proteins are indicated by black or coloured dots. Proteins of interest are indicated by red font. Blue dots correspond to some proteins identified also in the BDF6, HAT1 and EAF6 affinity purifications (see Fig. 4A). The ten most significant nucleoporins in the YFP-NUP110 affinity selection are marked in pale green.
